# Incremental and unifying modelling formalism for biological interaction networks

**DOI:** 10.1186/1471-2105-8-433

**Published:** 2007-11-08

**Authors:** Anastasia Yartseva, Hanna Klaudel, Raymond Devillers, François Képès

**Affiliations:** 1 IBISC – Université d'Évry Val d'Essonne, Tour Evry 2, 523 place des Terrasses de l'Agora, F-91000 Evry, France; 2 Département' d'Informatique, Université Libre de Bruxelles, CP212, B-1050 Bruxelles, Belgium; 3 Epigenomics Project, Genopole^®^, CNRS & Université d'Evry Val d'Essonne, France

## Abstract

**Background:**

An appropriate choice of the modeling formalism from the broad range of existing ones may be crucial for efficiently describing and analyzing biological systems.

**Results:**

We propose a new unifying and incremental formalism for the representation and modeling of biological interaction networks. This formalism allows automated translations into other formalisms, thus enabling a thorough study of the dynamic properties of a biological system. As a first illustration, we propose a translation into the R. Thomas' multivalued logical formalism which provides a possible semantics; a methodology for constructing such models is presented on a classical benchmark: the *λ *phage genetic switch. We also show how to extract from our model a classical ODE description of the dynamics of a system.

**Conclusion:**

This approach provides an additional level of description between the biological and mathematical ones. It yields, on the one hand, a knowledge expression in a form which is intuitive for biologists and, on the other hand, its representation in a formal and structured way.

## Background

Often, modeling approaches in biology try to fit the data into the Procrustean bed of a particular modeling formalism [1-5]. However, if the area of interest changes, the modeling process has to be continued (or even restarted) using a different modeling language, more adapted to the new area. An appropriate choice of the modeling formalism may be crucial for efficiently describing biological systems, avoiding to change the description language and permitting to reuse the previous work.

In this paper, we propose a modeling formalism for the biologists that enables the expression of various types of biological knowledge in a formal manner and its translation into target formalisms for analysis or simulation. It aims at satisfying the following requirements:

• universality: the integration of various kinds of biological data available today;

• parsimony: the simplest possible representation of the data;

• incrementality: the construction of more complex models from simpler ones;

• precision: expression of relations in a non-ambiguous (mathematical) way;

• transposability: formal rules for the translation of the information contained in the model into commonly used (target) modeling formalisms.

In such a formalism, the model can be seen rather as a well-organised knowledge base of information about the biological system. Every unit of information (which has no biological sense when divided) inside the model can be called a *data*. In this approach, we assume that there is neither contradictory nor "bad" data. In other words, every measurement, every observation may be true in some context.

Our approach, called Modular Interaction Network (MIN), is a formalism designed to represent biological data, having a bipartite network structure and admitting a graphical representation, even if not focused on it. MIN enables the integration of microscopic (molecular interactions) and macroscopic (system states) data, thus allowing to provide the desired level of abstraction. This abstraction allows to avoid the rather common problem of explosion of the model complexity [6]. MIN has a limited number of node and edges types, which enables to represent biological networks in a simple way, even if more detailed information can also be stored and recovered. MIN suits for the representation of genetic regulation as well as of metabolism with multi-molecular biological processes, in a natural and incremental manner. MIN is also provided with algorithms enabling a translation to two classical modeling formalisms: multi-level logical modeling [7] and differential equations. These translations can be performed at any stage of the modeling process.

The paper is structured as follows. After recalling the biology of the *λ *phage, which will be used as a running example, the formal MIN model is introduced. Next, the multi-level logical approach is first recalled and then used as a semantics of MIN. In Results section, the translation from MIN into multi-level logical approach is presented and extensively illustrated on the *λ *phage example. A translation to ordinary differential equations is then sketched. Finally, comparison with previous work, perspectives and some concluding remarks are presented.

### Biology of *λ *phage

In order to illustrate our approach, we shall use as a running example a classical biological benchmark: the genetic switch of the *λ *phage, which will be presented first.

The *λ *phage is a virus which infects the *Escherichia coli *bacteria. It turns out that a lot of quantitative and qualitative information is now available on it, so that it has become a benchmark organism and plays a central role in modeling [8,1,5,3,4,10].

**Figure 1:**
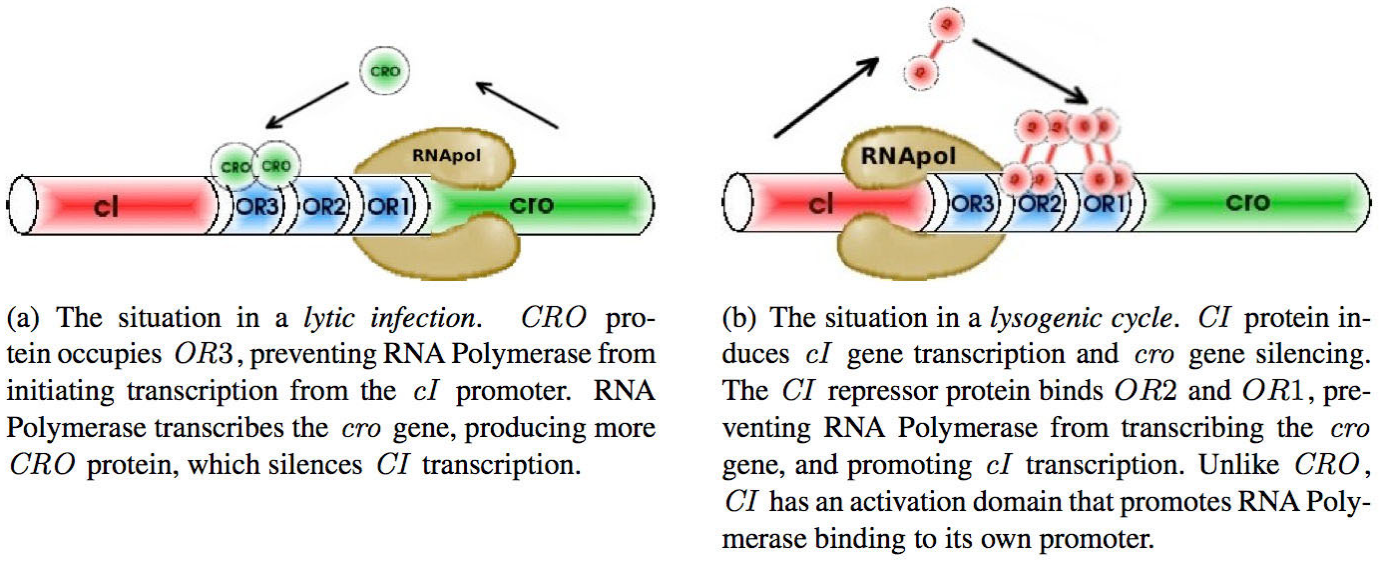
The genetic switch of the *λ *phage. The *cI *and *cro *genes lie on opposite sides of the operator region, containing three operators (*OR*1, *OR*2, *OR*3). The two genes are transcribed in opposite directions from their respective promoters, which overlap in the middle operator, *OR*2.

When a *λ *phage encounters a bacterium, it can attach itself to specific receptors on the bacterial membrane. At this moment, the virus genome enters the bacterium. Then, two alternative pathways are possible:

• *lytic pathway*: the virus uses the host machinery in order to replicate its genetic material and create new viruses. This phase takes about 45 minutes, then the bacterium is destroyed and about one hundred viruses are released in the external media (Figure 1(a)).

• *lysogenic pathway*: the virus integrates its genetic material in the bacterial genome. There is no production of viruses. The bacterium is said to be lysogenised. The virus can stay indefinitely in the genome of its host. But there exists an escape mechanism: in some cases, the virus can extract itself from the bacterial genome and enter a lytic phase as a response to some stimuli (Figure 1(b)).

A small region of the viral genome controls the decision between lytic or lyso-genic pathway. This region is composed of two genes and their two promoters (sites of regulation of the gene expression) and is referred to as the *genetic switch region *(see Figure 1). The decision results from the competition between two major proteins:

• the first one is referred to as *CRO*, encoded by gene *cro*, and expressed during lytic phase.

• the second one is called *λ *repressor, referred to as *CI*. It is encoded by gene *cI*, and it can activate other genes, including itself, and repress others. *cI *is expressed during lysogenic phase.

Note that the competition between *CI *and *CRO *is also influenced by the host environment. The host environment is captured through *CI *and *CRO *and their influence on the regulator region, i.e., the *genetic switch*.

## Methods

### Modular Interaction Network (MIN)

Modular Interaction Network (MIN) formalism considers two types of entities: variables (chemical species and regulatory sites) and influences (IRCs and ICRs). Every model entity (site, species, influence) is characterised by its *attributes *which can be any data concerning the biological object or interaction represented by this entity; for example:

• physical attributes: size and shape for a protein, position in DNA for a genomic sequence;

• localization in space (cell compartments: nucleus, cytosol);

• expression pattern (cell types, tissues etc.);

• observable values of the activity level for the biological object;

• velocity, force, speed, amplification factor, cooperativity increase, energy of the interaction.

From the very beginning, for any bit of information added to the model, the link to the source (the set of *references *to papers, databases, etc.) of it should be specified. This will be important in later steps of the modeling, for example in order to estimate the data quality. We assume that all the data in the model has a representation which allows it to be compared (it may be, for instance, a textual "string" representation).

#### Variables

Both species and regulatory sites may represent biological objects of some abstraction level (molecules or parts of them, complex processes like regulatory pathways, complex systems like sensors, or even an entire organism). As our knowledge about biological systems is based on *observations *and *experiments*, the *observable level of activity *of biological objects can change in different states of the biological system. These objects can influence the levels of activity of the other biological objects. So, every species and site in MIN will be assumed to have a set of *observable values*, corresponding to the observable levels of activity of the corresponding biological objects.

The formal definition of a MIN variable reflects the presence of various features (attributes) in biological objects. Also, in different sources a biological object can have different names (hence the name set of a variable). Moreover, the measurement methods used to observe the activity level of this object yield a set of possible values for the variable, usually (partially) ordered.

##### Definition 1

*A *variable *V is an entity characterized by a tuple *(*N*, *W*, *P*, *L*) *where:*

• *N is a non-empty set of known *names *of the variable*;

• *W is a partially ordered (by ≺_*V*_) set of *observable values *representing the activity level of the biological object associated to the variable. We shall assume that this set has at least the default value *undef, *unordered with respect to the other values, and two defined values, meaning that the variable is not a constant;*

• *P is a set of *attributes, *having a *type, *a *value *and the boolean *unique *field. unique *= 1 *indicates that this attribute can not be present in P more than once. Otherwise, several attributes of the same type can have different values;*

• *L is a non-empty set of *links *to (bibliographic) sources of the information about the variable. This set of attributes will always include the kind of the variable (which is unique and can be either "regulatory site" or "chemical species")*.

##### Chemical species

A species represents a biological object with catalytic or binding capabilities, which influence one or more regulatory sites. These influences have a chemical nature: association/dissociation reactions, electron transfers, etc. A species may have one or more influence capabilities, that will be called *affinities*.

An affinity is the ability of a biological object to interact with (potentially) a set of other biological objects through a particular regulatory site. Thus, an affinity may correspond to a protein domain for a protein or a surface molecule (receptor) for a cell.

##### Definition 2

*An *affinity *a is a tuple *(*l*_*a*_, *P*_*a*_, *L*_*a*_) *where*:

• *l*_*a *_*is a *label *representing the affinity name (which is indeed the label of the binding regulatory site);*

• *P*_*a *_*is a set of *attributes *of the affinity, having a *type *and a *value *(not necessarily unique);*

• *L*_*a *_*is a non-empty set of *links *on sources of the information about this affinity (bibliographic references)*.

Now we are able to formally introduce chemical species:

##### Definition 3

*A *chemical species *C is a variable *(*N*_*C*_, *W*_*C*_, *P*_*C*_, *L*_*C*_) *whose set of attributes P*_*C *_*contains *(*Kind*, "chemical species", 1) *and one or more data *(*Affinity*, *a*, 0), *where different a's enumerate the influence abilities of the species C*.

**Figure 2:**
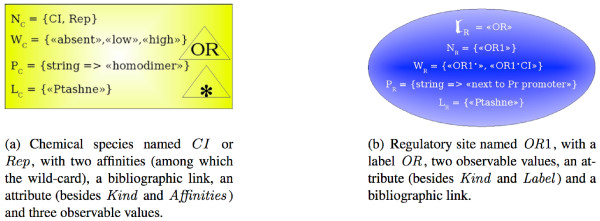
Representation of a chemical species and of a regulatory site.

Chemical species are graphically represented by rectangular boxes. Various affinities can be represented inside the species (by named triangles) omitting all the details except for their label. The nature of the interaction between two biological entities can be unknown. So, a wild-card affinity, labeled "*", may be defined for every species, standing for an unknown mechanism of regulation (see Figure 2 for an example of a chemical species).

##### Regulatory sites

A *regulatory site *regulates species activity in a manner which cannot be represented by a chemical reaction, like for example by three-dimensional conformation changes in a molecule or cooperativity effects. A regulatory site may represent a genome region or a protein domain that changes its state after a chemical reaction.

A regulatory site has a *label *which characterizes its capabilities of being influenced through *affinities*. If a regulatory site and an affinity of a species have the same label, it means that the interaction is possible between the biological objects corresponding to the site and the species. A regulatory site represents an "input" for a species and regulates its activity through integration of several influences on it.

##### Definition 4

*A *regulatory site *R is a variable *(*N*_*R*_, *W*_*R*_, *P*_*R*_, *L*_*R*_) *with the attributes *(*Kind*, "regulatory site", 1) *and *(*Label*, *l*_*R*_, 1) *in the set P*_*R*_, *where l*_*R *_*is a label representing the site type*.

Regulatory sites are graphically represented by ellipses containing the label *l*_*R *_inside a triangle. An example of a regulatory site is given on the Figure 2. The presented site has two different states: free (*OR*1·) and regulated ((*OR*1·*CI*)). This means that the corresponding biological object can participate in binding with another object. The label of this site is *OR*, so it can be influenced by a species having an affinity labeled *OR*, like the one represented on Figure 2.

In the MIN representation, different biological objects are associated to different entities in the model. The attributes of sites and species may have types like "position", "size", "location" etc. expressing a knowledge about these biological objects. For example, if a gene has more than one regulatory site of the same type in its regulatory region, several sites will be present in the model, having the same label but with different positions (mentioned in the attribute set); clearly, in this case, the corresponding variables will not be compatible. All these sites will influence the species corresponding to the gene. However, several species with the same name may be present in MIN, if they have attributes with different values. So, we can represent a molecule of the same protein in free or dimerised state, or the same gene at its natural location and translocated in a different place in the genome.

#### Influences

Biological objects, represented by species and sites in MIN, may interact and play specific roles in these interactions. For example, they can take part in a chemical reaction, one object modifying, creating or destroying another one. We assume that every interaction happens through an affinity and a regulatory site. More formally, a chemical species *C*_1 _having an affinity *a *with a label *l*_*a *_can influence a chemical species *C*_2 _if there is a regulatory site *R *labeled by the same label (*l*_*R *_= *l*_*a*_) which influences the species *C*_2_. An influence is defined between two MIN variables as follows:

##### Definition 5

*An *influence *I between variables is a tuple *(*V*, *V'*, *P*, *L*) *where:*

• *V is the *influencing *variable;*

• *V' is the variable *influenced *by V;*

• *P is the set of influence *attributes, *having a *type *and a *value *(not necessarily unique);*

• *L is the set of *links *to sources of the information about the influence*.

The influence (ICR) of a species on a regulatory site of another species represents the chemical interaction between two biological objects in which the state of the regulatory site is modified by the species through an affinity. Symmetrically, a regulatory site can *influence *the value of a species, through the influence (IRC) of a regulatory site on a chemical species. In this case the interaction between corresponding biological objects cannot be represented by a chemical reaction, and there is no specific affinity associated to such an influence.

##### Definition 6

• *An *influence *ICR of a Chemical species C*_*ICR *_*on a Regulatory site R*_*ICR *_*is an influence *(*C*_*ICR*_, *R*_*ICR*_, *P*_*ICR*_, *L*_*ICR*_) *with an attribute *(*Affinity*, *a*_*ICR*_) ∈ *P*_*ICR *_*which is the *affinity *involved in the interaction of the species C*_*ICR *_*and the site R*_*ICR*_, *hence with *(*Affinity*, *a*_*ICR*_, 0) ∈ PCICR
 MathType@MTEF@5@5@+=feaafiart1ev1aaatCvAUfKttLearuWrP9MDH5MBPbIqV92AaeXatLxBI9gBaebbnrfifHhDYfgasaacPC6xNi=xH8viVGI8Gi=hEeeu0xXdbba9frFj0xb9qqpG0dXdb9aspeI8k8fiI+fsY=rqGqVepae9pg0db9vqaiVgFr0xfr=xfr=xc9adbaqaaeGacaGaaiaabeqaaeqabiWaaaGcbaGaemiuaa1aaSbaaSqaaiabdoeadnaaBaaameaacqWGjbqscqWGdbWqcqWGsbGuaeqaaaWcbeaaaaa@31C6@*and either *laICR=lRICR
 MathType@MTEF@5@5@+=feaafiart1ev1aaatCvAUfKttLearuWrP9MDH5MBPbIqV92AaeXatLxBI9gBaebbnrfifHhDYfgasaacPC6xNi=xH8viVGI8Gi=hEeeu0xXdbba9frFj0xb9qqpG0dXdb9aspeI8k8fiI+fsY=rqGqVepae9pg0db9vqaiVgFr0xfr=xfr=xc9adbaqaaeGacaGaaiaabeqaaeqabiWaaaGcbaGaemiBaW2aaSbaaSqaaiabdggaHnaaBaaameaacqWGjbqscqWGdbWqcqWGsbGuaeqaaaWcbeaakiabg2da9iabdYgaSnaaBaaaleaacqWGsbGudaWgaaadbaGaemysaKKaem4qamKaemOuaifabeaaaSqabaaaaa@3993@*or a*_*ICR *_= *.

• *An *influence *IRC of the regulatory site R*_*IRC *_*on the species C*_*IRC *_*is an influence *(*R*_*IRC*_, *C*_*IRC*_, *P*_*IRC*_, *L*_*IRC*_) *with the attribute *(*Kind*, *IRC*) ∈ *P*_*IRC*_.

**Figure 3:**
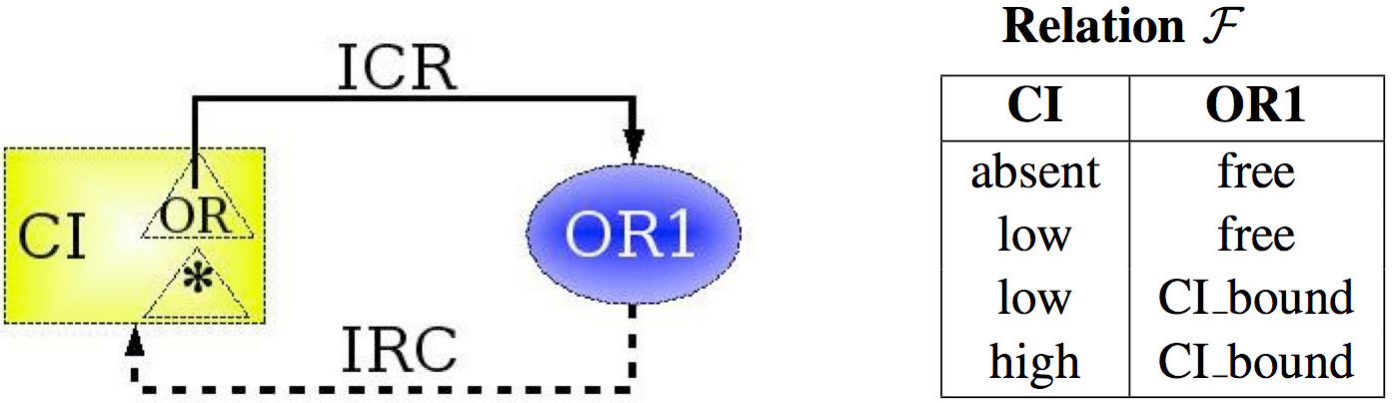
A small interaction network representing the chemical species *CI *and the (regulatory) site named *OR*1. **Left**. The influence ICR links the affinity labeled *OR *of species *CI *with the site *OR*1, and the influence IRC links the site *OR*1 and the species *CI*. In the *λ *switch, the regulatory site *OR*1 corresponds to the regulatory region in the DNA molecule coding for the protein *CI*. Thus, *CI *can influence the regulatory site *OR*1, and the activity of *CI *can be regulated through the regulatory site *OR*1. **Right**. The corresponding relation ℱ
 MathType@MTEF@5@5@+=feaafiart1ev1aaatCvAUfKttLearuWrP9MDH5MBPbIqV92AaeXatLxBI9gBaebbnrfifHhDYfgasaacPC6xNi=xH8viVGI8Gi=hEeeu0xXdbba9frFj0xb9qqpG0dXdb9aspeI8k8fiI+fsY=rqGqVepae9pg0db9vqaiVgFr0xfr=xfr=xc9adbaqaaeGacaGaaiaabeqaaeqabiWaaaGcbaWenfgDOvwBHrxAJfwnHbqeg0uy0HwzTfgDPnwy1aaceaGae8xmHyeaaa@36AD@ indicating the biologically observed states of the network.

An influence has a set of attributes, which should describe, in particular, the relationship between the values of the species and those of the regulatory site, like the parameters of the corresponding chemical reaction: kinetic rate or speed, or stoichiometric coefficients. Several examples of the IRCs and ICRs are shown on the Figure 3, by dashed and plain arcs, respectively.

#### The network

After presenting the species and the regulatory sites, the influences between them, we can now give a formal definition of the MIN for the modeling of a biological system. The information about the possible connections between species of the system is already coded in the labels of the regulatory sites and affinities. We consider that the states of the model are expressed through observable values of species and sites, so that Ω_*C *_denotes the set of functions associating a value of its value set to each species of the model, Ω_*R *_is the same for the sites of the model, and Ω is the set of all possible *observable states *of the model. In the following, *ω *∈ Ω stands for any given observable state of the system and *ω*(*V*) will stand for the value of the variable *V *in the state *ω*.

In general, in a single biological experiment (an *observation*), the values of only a subset of biological objects are measured. In this case, the observable values of non observed species and sites take the special value "*undef*" and the state of the system will be considered as "partly" defined.

In the set Ω of observable system states a subset ℱ
 MathType@MTEF@5@5@+=feaafiart1ev1aaatCvAUfKttLearuWrP9MDH5MBPbIqV92AaeXatLxBI9gBaebbnrfifHhDYfgasaacPC6xNi=xH8viVGI8Gi=hEeeu0xXdbba9frFj0xb9qqpG0dXdb9aspeI8k8fiI+fsY=rqGqVepae9pg0db9vqaiVgFr0xfr=xfr=xc9adbaqaaeGacaGaaiaabeqaaeqabiWaaaGcbaWenfgDOvwBHrxAJfwnHbqeg0uy0HwzTfgDPnwy1aaceaGae8xmHyeaaa@36AD@ ⊂ Ω of *observed system states *will yield all the partly defined system states which were really observed in biological experiments and described by biologists. ℱ
 MathType@MTEF@5@5@+=feaafiart1ev1aaatCvAUfKttLearuWrP9MDH5MBPbIqV92AaeXatLxBI9gBaebbnrfifHhDYfgasaacPC6xNi=xH8viVGI8Gi=hEeeu0xXdbba9frFj0xb9qqpG0dXdb9aspeI8k8fiI+fsY=rqGqVepae9pg0db9vqaiVgFr0xfr=xfr=xc9adbaqaaeGacaGaaiaabeqaaeqabiWaaaGcbaWenfgDOvwBHrxAJfwnHbqeg0uy0HwzTfgDPnwy1aaceaGae8xmHyeaaa@36AD@ plays the role of a databank from which the parameters of the dynamics of the system interactions could be inferred. If some of these parameters (as, for example, kinetic rates for biochemical reactions) are known (were measured in biology), they will be directly mentioned in the attributes of the corresponding influences (there will be some attribute of the kind (*Kinetic*_*rate*, 15) belonging to *P*_*ICR *_or *P*_*IRC*_, for instance).

##### Definition 7 (MIN)

*A Modular Interaction Network *ℳ
 MathType@MTEF@5@5@+=feaafiart1ev1aaatCvAUfKttLearuWrP9MDH5MBPbIqV92AaeXatLxBI9gBaebbnrfifHhDYfgasaacPC6xNi=xH8viVGI8Gi=hEeeu0xXdbba9frFj0xb9qqpG0dXdb9aspeI8k8fiI+fsY=rqGqVepae9pg0db9vqaiVgFr0xfr=xfr=xc9adbaqaaeGacaGaaiaabeqaaeqabiWaaaGcbaWenfgDOvwBHrxAJfwnHbqeg0uy0HwzTfgDPnwy1aaceaGae83mH0eaaa@36B6@*is a tuple *(V,ℐCℛ,ℐℛC,ℱ,ℒ
 MathType@MTEF@5@5@+=feaafiart1ev1aaatCvAUfKttLearuWrP9MDH5MBPbIqV92AaeXatLxBI9gBaebbnrfifHhDYfgasaacPC6xNi=xH8viVGI8Gi=hEeeu0xXdbba9frFj0xb9qqpG0dXdb9aspeI8k8fiI+fsY=rqGqVepae9pg0db9vqaiVgFr0xfr=xfr=xc9adbaqaaeGacaGaaiaabeqaaeqabiWaaaGcbaWenfgDOvwBHrxAJfwnHbqeg0uy0HwzTfgDPnwy1aaceaGae8xfXBLaeiilaWIae8heHKKae8NaXpKae83gHiLaeiilaWIae8heHKKae83gHiLae8NaXpKaeiilaWIae8xmHyKaeiilaWIae8NeHWeaaa@44AC@) *where*:

• V=C∪ℛ
 MathType@MTEF@5@5@+=feaafiart1ev1aaatCvAUfKttLearuWrP9MDH5MBPbIqV92AaeXatLxBI9gBaebbnrfifHhDYfgasaacPC6xNi=xH8viVGI8Gi=hEeeu0xXdbba9frFj0xb9qqpG0dXdb9aspeI8k8fiI+fsY=rqGqVepae9pg0db9vqaiVgFr0xfr=xfr=xc9adbaqaaeGacaGaaiaabeqaaeqabiWaaaGcbaWenfgDOvwBHrxAJfwnHbqeg0uy0HwzTfgDPnwy1aaceaGae8xfXBLaeyypa0Jae8NaXpKaeyOkIGSae83gHifaaa@3CE5@*is the set of variables of the model; it is partitioned in a set *C={Ci|i=1..|C|}
 MathType@MTEF@5@5@+=feaafiart1ev1aaatCvAUfKttLearuWrP9MDH5MBPbIqV92AaeXatLxBI9gBaebbnrfifHhDYfgasaacPC6xNi=xH8viVGI8Gi=hEeeu0xXdbba9frFj0xb9qqpG0dXdb9aspeI8k8fiI+fsY=rqGqVepae9pg0db9vqaiVgFr0xfr=xfr=xc9adbaqaaeGacaGaaiaabeqaaeqabiWaaaGcbaWenfgDOvwBHrxAJfwnHbqeg0uy0HwzTfgDPnwy1aaceaGae8NaXpKaeyypa0Jaei4EaSNaem4qam0aaSbaaSqaaiabdMgaPbqabaGccqGG8baFcqWGPbqAcqGH9aqpcqaIXaqmcqGGUaGlcqGGUaGlcqGG8baFcqWFce=qcqGG8baFcqGG9bqFaaa@4945@*of chemical species and a set *ℛ={Rj|j=1..|ℛ|}
 MathType@MTEF@5@5@+=feaafiart1ev1aaatCvAUfKttLearuWrP9MDH5MBPbIqV92AaeXatLxBI9gBaebbnrfifHhDYfgasaacPC6xNi=xH8viVGI8Gi=hEeeu0xXdbba9frFj0xb9qqpG0dXdb9aspeI8k8fiI+fsY=rqGqVepae9pg0db9vqaiVgFr0xfr=xfr=xc9adbaqaaeGacaGaaiaabeqaaeqabiWaaaGcbaWenfgDOvwBHrxAJfwnHbqeg0uy0HwzTfgDPnwy1aaceaGae83gHiLaeyypa0Jaei4EaSNaemOuai1aaSbaaSqaaiabdQgaQbqabaGccqGG8baFcqWGQbGAcqGH9aqpcqaIXaqmcqGGUaGlcqGGUaGlcqGG8baFcqWFBeIucqGG8baFcqGG9bqFaaa@4817@*of regulatory sites;*

• ℐCℛ⊆{ICRija|i=1..|C|,j=1..|ℛ|,(Affinity,a,0)∈PCi}
 MathType@MTEF@5@5@+=feaafiart1ev1aaatCvAUfKttLearuWrP9MDH5MBPbIqV92AaeXatLxBI9gBaebbnrfifHhDYfgasaacPC6xNi=xH8viVGI8Gi=hEeeu0xXdbba9frFj0xb9qqpG0dXdb9aspeI8k8fiI+fsY=rqGqVepae9pg0db9vqaiVgFr0xfr=xfr=xc9adbaqaaeGacaGaaiaabeqaaeqabiWaaaGcbaWenfgDOvwBHrxAJfwnHbqeg0uy0HwzTfgDPnwy1aaceaGae8heHKKae8NaXpKae83gHiLaeyOHI0Saei4EaSNaemysaKKaem4qamKaemOuai1aaSbaaSqaaiabdMgaPjabdQgaQjabdggaHbqabaGccqGG8baFcqWGPbqAcqGH9aqpcqaIXaqmcqGGUaGlcqGGUaGlcqGG8baFcqWFce=qcqGG8baFcqGGSaalcqWGQbGAcqGH9aqpcqaIXaqmcqGGUaGlcqGGUaGlcqGG8baFcqWFBeIucqGG8baFcqGGSaalcqGGOaakcqWGbbqqcqWGMbGzcqWGMbGzcqWGPbqAcqWGUbGBcqWGPbqAcqWG0baDcqWG5bqEcqGGSaalcqWGHbqycqGGSaalcqaIWaamcqGGPaqkcqGHiiIZcqWGqbaudaWgaaWcbaGaem4qam0aaSbaaWqaaiabdMgaPbqabaaaleqaaOGaeiyFa0haaa@721C@*is a set of influences from chemical species to regulatory sites through an affinity of the former and there is no more than one influence between such a pair of variables through the same affinity;*

• ℐℛC⊆{IRCjk|j=1..|ℛ|,k=1..|C|}
 MathType@MTEF@5@5@+=feaafiart1ev1aaatCvAUfKttLearuWrP9MDH5MBPbIqV92AaeXatLxBI9gBaebbnrfifHhDYfgasaacPC6xNi=xH8viVGI8Gi=hEeeu0xXdbba9frFj0xb9qqpG0dXdb9aspeI8k8fiI+fsY=rqGqVepae9pg0db9vqaiVgFr0xfr=xfr=xc9adbaqaaeGacaGaaiaabeqaaeqabiWaaaGcbaWenfgDOvwBHrxAJfwnHbqeg0uy0HwzTfgDPnwy1aaceaGae8heHKKae83gHiLae8NaXpKaeyOHI0Saei4EaSNaemysaKKaemOuaiLaem4qam0aaSbaaSqaaiabdQgaQjabdUgaRbqabaGccqGG8baFcqWGQbGAcqGH9aqpcqaIXaqmcqGGUaGlcqGGUaGlcqGG8baFcqWFBeIucqGG8baFcqGGSaalcqWGRbWAcqGH9aqpcqaIXaqmcqGGUaGlcqGGUaGlcqGG8baFcqWFce=qcqGG8baFcqGG9bqFaaa@5A0D@*is a set of influences from regulatory sites to chemical species and there is no more than one influence between such a pair of variables;*

• ℱ
 MathType@MTEF@5@5@+=feaafiart1ev1aaatCvAUfKttLearuWrP9MDH5MBPbIqV92AaeXatLxBI9gBaebbnrfifHhDYfgasaacPC6xNi=xH8viVGI8Gi=hEeeu0xXdbba9frFj0xb9qqpG0dXdb9aspeI8k8fiI+fsY=rqGqVepae9pg0db9vqaiVgFr0xfr=xfr=xc9adbaqaaeGacaGaaiaabeqaaeqabiWaaaGcbaWenfgDOvwBHrxAJfwnHbqeg0uy0HwzTfgDPnwy1aaceaGae8xmHyeaaa@36AD@ ⊂ Ω *is a set of observed partly defined states of the biological system;*

• ℒ
 MathType@MTEF@5@5@+=feaafiart1ev1aaatCvAUfKttLearuWrP9MDH5MBPbIqV92AaeXatLxBI9gBaebbnrfifHhDYfgasaacPC6xNi=xH8viVGI8Gi=hEeeu0xXdbba9frFj0xb9qqpG0dXdb9aspeI8k8fiI+fsY=rqGqVepae9pg0db9vqaiVgFr0xfr=xfr=xc9adbaqaaeGacaGaaiaabeqaaeqabiWaaaGcbaWenfgDOvwBHrxAJfwnHbqeg0uy0HwzTfgDPnwy1aaceaGae8NeHWeaaa@3694@*is a set of *links *to sources of the information about those observations*.

In figures, species will be represented by boxes, affinities by triangles inside the boxes of species, regulatory sites by ellipses, influences of a species on a regulatory site by plain arcs, and influences of a regulatory site on a species by dashed arcs. A small example of an interaction network is presented in Figure 3.

**Figure 4:**
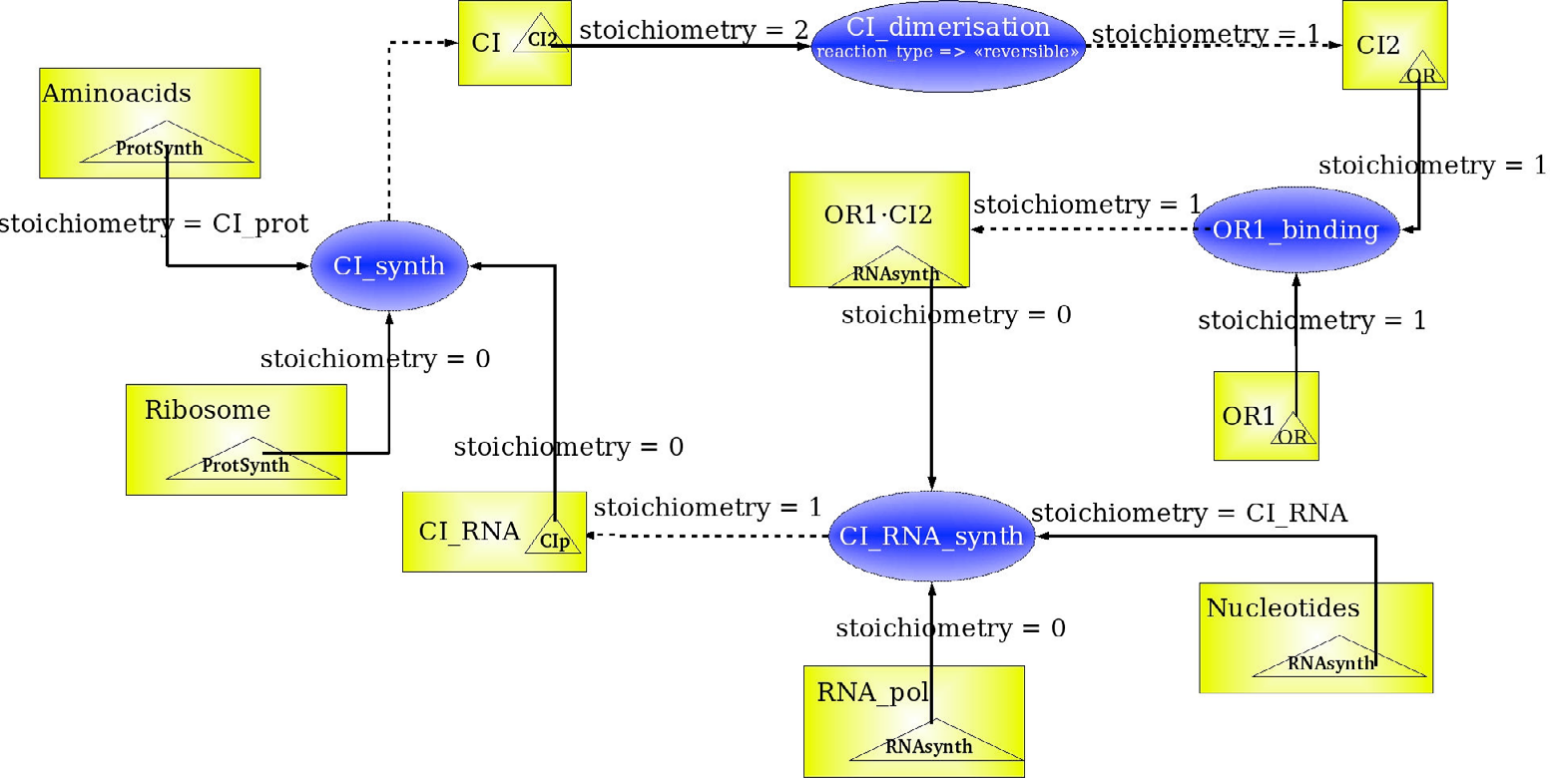
A MIN model representing the enzymatic reaction of CI synthesis. The reactions *CI*_*dimerisation *and *OR*1_*binding *are reversible, so they have the appropriate attribute. The reactions *CI*_*RNA*_*synth *and *CI*_*synth *are non reversible and have the appropriate attribute.

A MIN model having a highest level of detail has the property that each regulatory site corresponds to a (single) chemical reaction. We present an example of such a model in Figure 4. It illustrates the CI protein synthesis from the CI gene regulated by the OR1 regulatory site in function of the presence of CI protein dimer.

The corresponding chemical species are represented by chemical species of the MIN model. The biochemical reactions of this example are represented by regulatory sites, because a reaction is possible when all the substrates are present. This reaction regulates the level of activity of a chemical species by increasing or decreasing its quantity (concentration). Each reaction has an attribute "reversible" or "not reversible". For instance, if a reaction is reversible, this means that all the species connected to this reaction can be either products or substrates of the reaction. Another attribute of the regulatory site is a kinetic rate, which is in general a function of other mensurable parameters of the system such as concentrations of species catalyzing the reaction or even non participating directly in the reaction but influencing its kinetics. For example, such species can sequestrate one or more substrates or products or catalyze intermediate reaction steps. Another natural parameter of the kinetic rate function is the temperature: biochemical reactions go faster when the temperature increases.

On each influence adjacent to the regulatory site, an attribute corresponding to the stoichiometric coefficient is indicated. It may have 3 qualitatively different values:

• 0, which means that the corresponding species is an enzyme, i.e., it is not consumed or produced in this reaction, even if its presence is necessary for the reaction takes place;

• a numerical value, which corresponds to the number of molecules implicated in the reaction, generally one or two;

• any other label, standing for a vector of coefficients saying how many molecules of each of the 20 types of aminoacids (*a*_1_, *a*_2_,...,*a*_20_) or each of the 5 types of nucleotides (*n*_1_, *n*_2_, *n*_3_, *n*_4_, *n*_5_) is needed to synthesize the macromolecular product of the reaction.

For example, the stoichiometric coefficients for *Nucleotides *and *Aminoacids *in Figure 4 are labels, and each label represents the composition of the corresponding macromolecule: CI RNA or CI protein. In general, the opposite reaction of the biochemical synthesis is degradation, and it liberates the same quantities of the corresponding substrate residuals. The stoichiometric coefficients for *RNA_pol *or *Ribosome *are 0, which means that these are enzymes in the reactions of CI RNA synthesis and of CI protein synthesis. The stoichiometric coefficient for *CI *is 2 for the reaction of the dimerisation of CI, meaning that two molecules of CI are needed to form a dimer.

#### Compression of MINs

In order to simplify MIN models, it may be interesting to find the variables representing the same biological object and to combine them. So, the following defini-tion introduces the syntactic *compatibility *and the *union *of variables.

##### Definition 8 (Compatibility and union of variables)

*Let *{*V*_*i *_| *i *= 1, 2,...,*k*} *be the set of variables of the MIN *ℳ
 MathType@MTEF@5@5@+=feaafiart1ev1aaatCvAUfKttLearuWrP9MDH5MBPbIqV92AaeXatLxBI9gBaebbnrfifHhDYfgasaacPC6xNi=xH8viVGI8Gi=hEeeu0xXdbba9frFj0xb9qqpG0dXdb9aspeI8k8fiI+fsY=rqGqVepae9pg0db9vqaiVgFr0xfr=xfr=xc9adbaqaaeGacaGaaiaabeqaaeqabiWaaaGcbaWenfgDOvwBHrxAJfwnHbqeg0uy0HwzTfgDPnwy1aaceaGae83mH0eaaa@36B6@, *with V*_*i *_= (*N*_*i*_, *W*_*i*_, *P*_*i*_, *L*_*i*_). *The variables in this set will be said to be *compatible *if they have the same names *(∀*V*_*i*_, *V*_*j *_NVi=NVj
 MathType@MTEF@5@5@+=feaafiart1ev1aaatCvAUfKttLearuWrP9MDH5MBPbIqV92AaeXatLxBI9gBaebbnrfifHhDYfgasaacPC6xNi=xH8viVGI8Gi=hEeeu0xXdbba9frFj0xb9qqpG0dXdb9aspeI8k8fiI+fsY=rqGqVepae9pg0db9vqaiVgFr0xfr=xfr=xc9adbaqaaeGacaGaaiaabeqaaeqabiWaaaGcbaGaemOta40aaSbaaSqaaiabdAfawnaaBaaameaacqWGPbqAaeqaaaWcbeaakiabg2da9iabd6eaonaaBaaaleaacqWGwbGvdaWgaaadbaGaemOAaOgabeaaaSqabaaaaa@3517@), *their unique attributes are compatible *((*x*, *y*, 1) ∈ *P*_*i *_∧ (*x*, *z*, *b*) ∈ *P*_*j *_⇒ *y *= *z *∧ *b *= 1), *if their partial orders are compatible *((∪i=1k<Vi)∗
 MathType@MTEF@5@5@+=feaafiart1ev1aaatCvAUfKttLearuWrP9MDH5MBPbIqV92AaeXatLxBI9gBaebbnrfifHhDYfgasaacPC6xNi=xH8viVGI8Gi=hEeeu0xXdbba9frFj0xb9qqpG0dXdb9aspeI8k8fiI+fsY=rqGqVepae9pg0db9vqaiVgFr0xfr=xfr=xc9adbaqaaeGacaGaaiaabeqaaeqabiWaaaGcbaWaaeWaaeaadaWeWaqaaiabgYda8maaBaaaleaacqWGwbGvdaWgaaadbaGaemyAaKgabeaaaSqabaaabaGaemyAaKMaeyypa0JaeGymaedabaGaem4AaSganiablQIivbaakiaawIcacaGLPaaadaahaaWcbeqaaiabgEHiQaaaaaa@38B2@*is acyclic*) *and their observed values are compatible *(∀*V*_*i*_, *V*_*j*_∀(...,*w*_*i*_,...,*w*_*j*_,...) ∈ ℱ
 MathType@MTEF@5@5@+=feaafiart1ev1aaatCvAUfKttLearuWrP9MDH5MBPbIqV92AaeXatLxBI9gBaebbnrfifHhDYfgasaacPC6xNi=xH8viVGI8Gi=hEeeu0xXdbba9frFj0xb9qqpG0dXdb9aspeI8k8fiI+fsY=rqGqVepae9pg0db9vqaiVgFr0xfr=xfr=xc9adbaqaaeGacaGaaiaabeqaaeqabiWaaaGcbaWenfgDOvwBHrxAJfwnHbqeg0uy0HwzTfgDPnwy1aaceaGae8xmHyeaaa@36AD@*either w*_*i *_= *undef or w*_*j *_= *undef or w*_*i *_= *w*_*j*_). *In such a case, their *union ∪i=1kVi=(∪i=1kNi,∪i=1kWi,∪i=1kPi,∪i=1kLi)
 MathType@MTEF@5@5@+=feaafiart1ev1aaatCvAUfKttLearuWrP9MDH5MBPbIqV92AaeXatLxBI9gBaebbnrfifHhDYfgasaacPC6xNi=xH8viVGI8Gi=hEeeu0xXdbba9frFj0xb9qqpG0dXdb9aspeI8k8fiI+fsY=rqGqVepae9pg0db9vqaiVgFr0xfr=xfr=xc9adbaqaaeGacaGaaiaabeqaaeqabiWaaaGcbaWaambmaeaacqWGwbGvdaWgaaWcbaGaemyAaKgabeaaaeaacqWGPbqAcqGH9aqpcqaIXaqmaeaacqWGRbWAa0GaeSOkIufakiabg2da9maabmaabaWaambmaeaacqWGobGtdaWgaaWcbaGaemyAaKgabeaaaeaacqWGPbqAcqGH9aqpcqaIXaqmaeaacqWGRbWAa0GaeSOkIufakiabcYcaSmaatadabaGaem4vaC1aaSbaaSqaaiabdMgaPbqabaaabaGaemyAaKMaeyypa0JaeGymaedabaGaem4AaSganiablQIivbGccqGGSaaldaWeWaqaaiabdcfaqnaaBaaaleaacqWGPbqAaeqaaaqaaiabdMgaPjabg2da9iabigdaXaqaaiabdUgaRbqdcqWIQisvaOGaeiilaWYaambmaeaacqWGmbatdaWgaaWcbaGaemyAaKgabeaaaeaacqWGPbqAcqGH9aqpcqaIXaqmaeaacqWGRbWAa0GaeSOkIufaaOGaayjkaiaawMcaaaaa@5DCA@, *with *<∪i=1kVi=(∪i=1k<Vi)∗
 MathType@MTEF@5@5@+=feaafiart1ev1aaatCvAUfKttLearuWrP9MDH5MBPbIqV92AaeXatLxBI9gBaebbnrfifHhDYfgasaacPC6xNi=xH8viVGI8Gi=hEeeu0xXdbba9frFj0xb9qqpG0dXdb9aspeI8k8fiI+fsY=rqGqVepae9pg0db9vqaiVgFr0xfr=xfr=xc9adbaqaaeGacaGaaiaabeqaaeqabiWaaaGcbaGaeyipaWZaaSbaaSqaamaatadabaGaemOvay1aaSbaaWqaaiabdMgaPbqabaaabaGaemyAaKMaeyypa0JaeGymaedabaGaem4AaSgaoiablQIivbaaleqaaOGaeyypa0ZaaeWaaeaadaWeWaqaaiabgYda8maaBaaaleaacqWGwbGvdaWgaaadbaGaemyAaKgabeaaaSqabaaabaGaemyAaKMaeyypa0JaeGymaedabaGaem4AaSganiablQIivbaakiaawIcacaGLPaaadaahaaWcbeqaaiabgEHiQaaaaaa@43F3@.

As the values of variables come from different biological experiments, in order to compare them we need to use the same approximations as generally accepted by biological science. This means that the "equality" of values *w*_*i *_= *w*_*j *_should be confirmed by a biologist when it is not obvious. Notice also that chemical species may only be compatible with other chemical species, and similarly for regulatory sites.

This definition will sometimes allow to reduce the representation of a MIN, by replacing compatible sets of variables by their union. Moreover, the translation of MIN representation in other formalism can allow further compression of variables depending on the capability of the formalism to distinguish between different biological objects.

Thus, the *simplification *is an operation on MIN ℳ
 MathType@MTEF@5@5@+=feaafiart1ev1aaatCvAUfKttLearuWrP9MDH5MBPbIqV92AaeXatLxBI9gBaebbnrfifHhDYfgasaacPC6xNi=xH8viVGI8Gi=hEeeu0xXdbba9frFj0xb9qqpG0dXdb9aspeI8k8fiI+fsY=rqGqVepae9pg0db9vqaiVgFr0xfr=xfr=xc9adbaqaaeGacaGaaiaabeqaaeqabiWaaaGcbaWenfgDOvwBHrxAJfwnHbqeg0uy0HwzTfgDPnwy1aaceaGae83mH0eaaa@36B6@ which produces MIN ℳ
 MathType@MTEF@5@5@+=feaafiart1ev1aaatCvAUfKttLearuWrP9MDH5MBPbIqV92AaeXatLxBI9gBaebbnrfifHhDYfgasaacPC6xNi=xH8viVGI8Gi=hEeeu0xXdbba9frFj0xb9qqpG0dXdb9aspeI8k8fiI+fsY=rqGqVepae9pg0db9vqaiVgFr0xfr=xfr=xc9adbaqaaeGacaGaaiaabeqaaeqabiWaaaGcbaWenfgDOvwBHrxAJfwnHbqeg0uy0HwzTfgDPnwy1aaceaGae83mH0eaaa@36B6@' in a following way:

• First of all, the compatible variables of the MIN ℳ
 MathType@MTEF@5@5@+=feaafiart1ev1aaatCvAUfKttLearuWrP9MDH5MBPbIqV92AaeXatLxBI9gBaebbnrfifHhDYfgasaacPC6xNi=xH8viVGI8Gi=hEeeu0xXdbba9frFj0xb9qqpG0dXdb9aspeI8k8fiI+fsY=rqGqVepae9pg0db9vqaiVgFr0xfr=xfr=xc9adbaqaaeGacaGaaiaabeqaaeqabiWaaaGcbaWenfgDOvwBHrxAJfwnHbqeg0uy0HwzTfgDPnwy1aaceaGae83mH0eaaa@36B6@ are combined;

• then, the ICRs (IRCs) of a variable *V*_1 _on *V*_2 _of the MIN ℳ
 MathType@MTEF@5@5@+=feaafiart1ev1aaatCvAUfKttLearuWrP9MDH5MBPbIqV92AaeXatLxBI9gBaebbnrfifHhDYfgasaacPC6xNi=xH8viVGI8Gi=hEeeu0xXdbba9frFj0xb9qqpG0dXdb9aspeI8k8fiI+fsY=rqGqVepae9pg0db9vqaiVgFr0xfr=xfr=xc9adbaqaaeGacaGaaiaabeqaaeqabiWaaaGcbaWenfgDOvwBHrxAJfwnHbqeg0uy0HwzTfgDPnwy1aaceaGae83mH0eaaa@36B6@ are linked to the variables V′1
 MathType@MTEF@5@5@+=feaafiart1ev1aaatCvAUfKttLearuWrP9MDH5MBPbIqV92AaeXatLxBI9gBaebbnrfifHhDYfgasaacPC6xNi=xH8viVGI8Gi=hEeeu0xXdbba9frFj0xb9qqpG0dXdb9aspeI8k8fiI+fsY=rqGqVepae9pg0db9vqaiVgFr0xfr=xfr=xc9adbaqaaeGacaGaaiaabeqaaeqabiWaaaGcbaGafmOvayLbauaadaWgaaWcbaGaeGymaedabeaaaaa@2E30@ and V′2
 MathType@MTEF@5@5@+=feaafiart1ev1aaatCvAUfKttLearuWrP9MDH5MBPbIqV92AaeXatLxBI9gBaebbnrfifHhDYfgasaacPC6xNi=xH8viVGI8Gi=hEeeu0xXdbba9frFj0xb9qqpG0dXdb9aspeI8k8fiI+fsY=rqGqVepae9pg0db9vqaiVgFr0xfr=xfr=xc9adbaqaaeGacaGaaiaabeqaaeqabiWaaaGcbaGafmOvayLbauaadaWgaaWcbaGaeGOmaidabeaaaaa@2E32@ of ℳ
 MathType@MTEF@5@5@+=feaafiart1ev1aaatCvAUfKttLearuWrP9MDH5MBPbIqV92AaeXatLxBI9gBaebbnrfifHhDYfgasaacPC6xNi=xH8viVGI8Gi=hEeeu0xXdbba9frFj0xb9qqpG0dXdb9aspeI8k8fiI+fsY=rqGqVepae9pg0db9vqaiVgFr0xfr=xfr=xc9adbaqaaeGacaGaaiaabeqaaeqabiWaaaGcbaWenfgDOvwBHrxAJfwnHbqeg0uy0HwzTfgDPnwy1aaceaGae83mH0eaaa@36B6@', where V′1
 MathType@MTEF@5@5@+=feaafiart1ev1aaatCvAUfKttLearuWrP9MDH5MBPbIqV92AaeXatLxBI9gBaebbnrfifHhDYfgasaacPC6xNi=xH8viVGI8Gi=hEeeu0xXdbba9frFj0xb9qqpG0dXdb9aspeI8k8fiI+fsY=rqGqVepae9pg0db9vqaiVgFr0xfr=xfr=xc9adbaqaaeGacaGaaiaabeqaaeqabiWaaaGcbaGafmOvayLbauaadaWgaaWcbaGaeGymaedabeaaaaa@2E30@ is compatible with *V*_1 _and V′2
 MathType@MTEF@5@5@+=feaafiart1ev1aaatCvAUfKttLearuWrP9MDH5MBPbIqV92AaeXatLxBI9gBaebbnrfifHhDYfgasaacPC6xNi=xH8viVGI8Gi=hEeeu0xXdbba9frFj0xb9qqpG0dXdb9aspeI8k8fiI+fsY=rqGqVepae9pg0db9vqaiVgFr0xfr=xfr=xc9adbaqaaeGacaGaaiaabeqaaeqabiWaaaGcbaGafmOvayLbauaadaWgaaWcbaGaeGOmaidabeaaaaa@2E32@ is compatible with *V*_2_;

• the relation ℱ
 MathType@MTEF@5@5@+=feaafiart1ev1aaatCvAUfKttLearuWrP9MDH5MBPbIqV92AaeXatLxBI9gBaebbnrfifHhDYfgasaacPC6xNi=xH8viVGI8Gi=hEeeu0xXdbba9frFj0xb9qqpG0dXdb9aspeI8k8fiI+fsY=rqGqVepae9pg0db9vqaiVgFr0xfr=xfr=xc9adbaqaaeGacaGaaiaabeqaaeqabiWaaaGcbaWenfgDOvwBHrxAJfwnHbqeg0uy0HwzTfgDPnwy1aaceaGae8xmHyeaaa@36AD@ is updated: the entries containing a pair of combined variables with different observed values are splitted in two entries where only one value at a time is listed for the combined variable.

The formal definition of MIN simplification is presented below.

##### Definition 9 (Simplification of MIN)

*If *ℳ
 MathType@MTEF@5@5@+=feaafiart1ev1aaatCvAUfKttLearuWrP9MDH5MBPbIqV92AaeXatLxBI9gBaebbnrfifHhDYfgasaacPC6xNi=xH8viVGI8Gi=hEeeu0xXdbba9frFj0xb9qqpG0dXdb9aspeI8k8fiI+fsY=rqGqVepae9pg0db9vqaiVgFr0xfr=xfr=xc9adbaqaaeGacaGaaiaabeqaaeqabiWaaaGcbaWenfgDOvwBHrxAJfwnHbqeg0uy0HwzTfgDPnwy1aaceaGae83mH0eaaa@36B6@ = (V,ℐCℛ,ℐℛC,ℱ,ℒ
 MathType@MTEF@5@5@+=feaafiart1ev1aaatCvAUfKttLearuWrP9MDH5MBPbIqV92AaeXatLxBI9gBaebbnrfifHhDYfgasaacPC6xNi=xH8viVGI8Gi=hEeeu0xXdbba9frFj0xb9qqpG0dXdb9aspeI8k8fiI+fsY=rqGqVepae9pg0db9vqaiVgFr0xfr=xfr=xc9adbaqaaeGacaGaaiaabeqaaeqabiWaaaGcbaWenfgDOvwBHrxAJfwnHbqeg0uy0HwzTfgDPnwy1aaceaGae8xfXBLaeiilaWIae8heHKKae8NaXpKae83gHiLaeiilaWIae8heHKKae83gHiLae8NaXpKaeiilaWIae8xmHyKaeiilaWIae8NeHWeaaa@44AC@) *is a MIN*, V′=C′∪ℛ′
 MathType@MTEF@5@5@+=feaafiart1ev1aaatCvAUfKttLearuWrP9MDH5MBPbIqV92AaeXatLxBI9gBaebbnrfifHhDYfgasaacPC6xNi=xH8viVGI8Gi=hEeeu0xXdbba9frFj0xb9qqpG0dXdb9aspeI8k8fiI+fsY=rqGqVepae9pg0db9vqaiVgFr0xfr=xfr=xc9adbaqaaeGacaGaaiaabeqaaeqabiWaaaGcbaWenfgDOvwBHrxAJfwnHbqeg0uy0HwzTfgDPnwy1aaceaGaf8xfXBLbauaacqGH9aqpcuWFce=qgaqbaiabgQIiilqb=Triszaafaaaaa@3D09@*is a partition of *V
 MathType@MTEF@5@5@+=feaafiart1ev1aaatCvAUfKttLearuWrP9MDH5MBPbIqV92AaeXatLxBI9gBaebbnrfifHhDYfgasaacPC6xNi=xH8viVGI8Gi=hEeeu0xXdbba9frFj0xb9qqpG0dXdb9aspeI8k8fiI+fsY=rqGqVepae9pg0db9vqaiVgFr0xfr=xfr=xc9adbaqaaeGacaGaaiaabeqaaeqabiWaaaGcbaWenfgDOvwBHrxAJfwnHbqeg0uy0HwzTfgDPnwy1aaceaGae8xfXBfaaa@3771@*into sets of compatible variables in *ℳ
 MathType@MTEF@5@5@+=feaafiart1ev1aaatCvAUfKttLearuWrP9MDH5MBPbIqV92AaeXatLxBI9gBaebbnrfifHhDYfgasaacPC6xNi=xH8viVGI8Gi=hEeeu0xXdbba9frFj0xb9qqpG0dXdb9aspeI8k8fiI+fsY=rqGqVepae9pg0db9vqaiVgFr0xfr=xfr=xc9adbaqaaeGacaGaaiaabeqaaeqabiWaaaGcbaWenfgDOvwBHrxAJfwnHbqeg0uy0HwzTfgDPnwy1aaceaGae83mH0eaaa@36B6@, *then the *compressed *form of *ℳ
 MathType@MTEF@5@5@+=feaafiart1ev1aaatCvAUfKttLearuWrP9MDH5MBPbIqV92AaeXatLxBI9gBaebbnrfifHhDYfgasaacPC6xNi=xH8viVGI8Gi=hEeeu0xXdbba9frFj0xb9qqpG0dXdb9aspeI8k8fiI+fsY=rqGqVepae9pg0db9vqaiVgFr0xfr=xfr=xc9adbaqaaeGacaGaaiaabeqaaeqabiWaaaGcbaWenfgDOvwBHrxAJfwnHbqeg0uy0HwzTfgDPnwy1aaceaGae83mH0eaaa@36B6@*through the partition *V
 MathType@MTEF@5@5@+=feaafiart1ev1aaatCvAUfKttLearuWrP9MDH5MBPbIqV92AaeXatLxBI9gBaebbnrfifHhDYfgasaacPC6xNi=xH8viVGI8Gi=hEeeu0xXdbba9frFj0xb9qqpG0dXdb9aspeI8k8fiI+fsY=rqGqVepae9pg0db9vqaiVgFr0xfr=xfr=xc9adbaqaaeGacaGaaiaabeqaaeqabiWaaaGcbaWenfgDOvwBHrxAJfwnHbqeg0uy0HwzTfgDPnwy1aaceaGae8xfXBfaaa@3771@' *is the MIN *ℳ′=(V′,ℐCℛ′,ℐℛC′,ℱ′,ℒ)
 MathType@MTEF@5@5@+=feaafiart1ev1aaatCvAUfKttLearuWrP9MDH5MBPbIqV92AaeXatLxBI9gBaebbnrfifHhDYfgasaacPC6xNi=xH8viVGI8Gi=hEeeu0xXdbba9frFj0xb9qqpG0dXdb9aspeI8k8fiI+fsY=rqGqVepae9pg0db9vqaiVgFr0xfr=xfr=xc9adbaqaaeGacaGaaiaabeqaaeqabiWaaaGcbaWenfgDOvwBHrxAJfwnHbqeg0uy0HwzTfgDPnwy1aaceaGaf83mH0KbauaacqGH9aqpcqGGOaakcuWFveVvgaqbaiabcYcaSiab=brijjab=jq8djqb=TriszaafaGaeiilaWIae8heHKKae83gHiLaf8NaXpKbauaacqGGSaalcuWFXeIrgaqbaiabcYcaSiab=jrimjabcMcaPaaa@48C6@*defined as follows:*

• *each variable V' *∈ V
 MathType@MTEF@5@5@+=feaafiart1ev1aaatCvAUfKttLearuWrP9MDH5MBPbIqV92AaeXatLxBI9gBaebbnrfifHhDYfgasaacPC6xNi=xH8viVGI8Gi=hEeeu0xXdbba9frFj0xb9qqpG0dXdb9aspeI8k8fiI+fsY=rqGqVepae9pg0db9vqaiVgFr0xfr=xfr=xc9adbaqaaeGacaGaaiaabeqaaeqabiWaaaGcbaWenfgDOvwBHrxAJfwnHbqeg0uy0HwzTfgDPnwy1aaceaGae8xfXBfaaa@3771@' *represents the union of compatible variables composing the set V' *(*V' *= ⋃_*V*∈*V'*_*V*);

• ℐCℛ′=def∪C′∈C′∪a:(Affinity,a,0)∈PC′ICR′C′,a where ICR′C′,a={(C′,R′,P′,L′)|R′∈ℛ′,X={(C,R,P,L)∈ℐCℛ|C∈C′,R∈R′,(Affinity,a)∈P(C,R,P,L)}≠∅,P′=∪ICR∈XPICR,L′=∪ICR∈XLICR}
 MathType@MTEF@5@5@+=feaafiart1ev1aaatCvAUfKttLearuWrP9MDH5MBPbIqV92AaeXatLxBI9gBaebbnrfifHhDYfgasaacPC6xNi=xH8viVGI8Gi=hEeeu0xXdbba9frFj0xb9qqpG0dXdb9aspeI8k8fiI+fsY=rqGqVepae9pg0db9vqaiVgFr0xfr=xfr=xc9adbaqaaeGacaGaaiaabeqaaeqabiWaaaGcbaWenfgDOvwBHrxAJfwnHbqeg0uy0HwzTfgDPnwy1aaceaGae8heHKKae8NaXpKaf83gHiLbauaadaWfGaqaaiabg2da9aWcbeqaaiabdsgaKjabdwgaLjabdAgaMbaakmaatababaWaambeaeaacqWGjbqscqWGdbWqcuWGsbGugaqbamaaBaaaleaacuWGdbWqgaqbaiabcYcaSiabdggaHbqabaGccqqGGaaiaSqaaiabdggaHjabcQda6iabcIcaOiabdgeabjabdAgaMjabdAgaMjabdMgaPjabd6gaUjabdMgaPjabdsha0jabdMha5jabcYcaSiabdggaHjabcYcaSiabicdaWiabcMcaPiabgIGiolabdcfaqnaaBaaameaacuWGdbWqgaqbaaqabaaaleqaniablQIivbaaleaacuWGdbWqgaqbaiabgIGiolqb=jq8dzaafaaabeqdcqWIQisvaOGaem4DaCNaemiAaGMaemyzauMaemOCaiNaemyzauMaeeiiaaIaemysaKKaem4qamKafmOuaiLbauaadaWgaaWcbaGafm4qamKbauaacqGGSaalcqWGHbqyaeqaaOGaeyypa0Jaei4EaSNaeiikaGIafm4qamKbauaacqGGSaalcuWGsbGugaqbaiabcYcaSiqbdcfaqzaafaGaeiilaWIafmitaWKbauaacqGGPaqkcqGG8baFcuWGsbGugaqbaiabgIGiolqb=TriszaafaGaeiilaWIaemiwaGLaeyypa0Jaei4EaSNaeiikaGIaem4qamKaeiilaWIaemOuaiLaeiilaWIaemiuaaLaeiilaWIaemitaWKaeiykaKIaeyicI4Sae8heHKKae8NaXpKae83gHiLaeiiFaWNaem4qamKaeyicI4Safm4qamKbauaacqGGSaalcqWGsbGucqGHiiIZcuWGsbGugaqbaiabcYcaSiabcIcaOiabdgeabjabdAgaMjabdAgaMjabdMgaPjabd6gaUjabdMgaPjabdsha0jabdMha5jabcYcaSiabdggaHjabcMcaPiabgIGiolabdcfaqnaaBaaaleaacqGGOaakcqWGdbWqcqGGSaalcqWGsbGucqGGSaalcqWGqbaucqGGSaalcqWGmbatcqGGPaqkaeqaaOGaeiyFa0NaeyiyIKRaeyybIySaeiilaWIafmiuaaLbauaacqGH9aqpdaWeqaqaaiabdcfaqnaaBaaaleaacqWGjbqscqWGdbWqcqWGsbGuaeqaaOGaeiilaWIafmitaWKbauaacqGH9aqpdaWeqaqaaiabdYeamnaaBaaaleaacqWGjbqscqWGdbWqcqWGsbGuaeqaaaqaaiabdMeajjabdoeadjabdkfasjabgIGiolabdIfaybqab0GaeSOkIufakiabc2ha9bWcbaGaemysaKKaem4qamKaemOuaiLaeyicI4SaemiwaGfabeqdcqWIQisvaaaa@E0A5@;

• ℐℛC′=def{(R′,C′,P′,L′)|R′∈ℛ′,C′∈C′,X={(R,C,P,L)∈ℐℛC|C∈C′,R∈R′}≠∅,P′=∪IRC∈XPIRC,L′=∪IRC∈XLIRC}
 MathType@MTEF@5@5@+=feaafiart1ev1aaatCvAUfKttLearuWrP9MDH5MBPbIqV92AaeXatLxBI9gBaebbnrfifHhDYfgasaacPC6xNi=xH8viVGI8Gi=hEeeu0xXdbba9frFj0xb9qqpG0dXdb9aspeI8k8fiI+fsY=rqGqVepae9pg0db9vqaiVgFr0xfr=xfr=xc9adbaqaaeGacaGaaiaabeqaaeqabiWaaaGcbaWenfgDOvwBHrxAJfwnHbqeg0uy0HwzTfgDPnwy1aaceaGae8heHKKae83gHiLaf8NaXpKbauaadaWfGaqaaiabg2da9aWcbeqaaiabdsgaKjabdwgaLjabdAgaMbaakiabcUha7jabcIcaOiqbdkfaszaafaGaeiilaWIafm4qamKbauaacqGGSaalcuWGqbaugaqbaiabcYcaSiqbdYeamzaafaGaeiykaKIaeiiFaWNafmOuaiLbauaacqGHiiIZcuWFBeIugaqbaiabcYcaSiqbdoeadzaafaGaeyicI4Saf8NaXpKbauaacqGGSaalcqWGybawcqGH9aqpcqGG7bWEcqGGOaakcqWGsbGucqGGSaalcqWGdbWqcqGGSaalcqWGqbaucqGGSaalcqWGmbatcqGGPaqkcqGHiiIZcqWFqesscqWFBeIucqWFce=qcqGG8baFcqWGdbWqcqGHiiIZcuWGdbWqgaqbaiabcYcaSiabdkfasjabgIGiolqbdkfaszaafaGaeiyFa0NaeyiyIKRaeyybIySaeiilaWIafmiuaaLbauaacqGH9aqpdaWeqaqaaiabdcfaqnaaBaaaleaacqWGjbqscqWGsbGucqWGdbWqaeqaaOGaeiilaWIafmitaWKbauaacqGH9aqpdaWeqaqaaiabdYeamnaaBaaaleaacqWGjbqscqWGsbGucqWGdbWqaeqaaaqaaiabdMeajjabdkfasjabdoeadjabgIGiolabdIfaybqab0GaeSOkIufakiabc2ha9bWcbaGaemysaKKaemOuaiLaem4qamKaeyicI4SaemiwaGfabeqdcqWIQisvaaaa@957C@;

• ℱ′=def{ω′=(w′1,...,w′|V′|)|∃(w1,...,w|V|)∈ℱ,∀i(∀Vj∈V′iw′i=wj=undef∨∃Vj∈V′iw′i=wj≠undef)}
 MathType@MTEF@5@5@+=feaafiart1ev1aaatCvAUfKttLearuWrP9MDH5MBPbIqV92AaeXatLxBI9gBaebbnrfifHhDYfgasaacPC6xNi=xH8viVGI8Gi=hEeeu0xXdbba9frFj0xb9qqpG0dXdb9aspeI8k8fiI+fsY=rqGqVepae9pg0db9vqaiVgFr0xfr=xfr=xc9adbaqaaeGacaGaaiaabeqaaeqabiWaaaGcbaWenfgDOvwBHrxAJfwnHbqeg0uy0HwzTfgDPnwy1aaceaGaf8xmHyKbauaadaWfGaqaaiabg2da9aWcbeqaaiabdsgaKjabdwgaLjabdAgaMbaakiabcUha7HGaciqb+L8a3zaafaGaeyypa0JaeiikaGIafm4DaCNbauaadaWgaaWcbaGaeGymaedabeaakiabcYcaSiabc6caUiabc6caUiabc6caUiabcYcaSiqbdEha3zaafaWaaSbaaSqaamaaemaabaGaf8xfXBLbauaaaiaawEa7caGLiWoaaeqaaOGaeiykaKIaeiiFaWNaey4aIqIaeiikaGIaem4DaC3aaSbaaSqaaiabigdaXaqabaGccqGGSaalcqGGUaGlcqGGUaGlcqGGUaGlcqGGSaalcqWG3bWDdaWgaaWcbaWaaqWaaeaacqWFveVvaiaawEa7caGLiWoaaeqaaOGaeiykaKIaeyicI4Sae8xmHyKaeiilaWIaeyiaIiIaemyAaKMaeiikaGIaeyiaIiIaemOvay1aaSbaaSqaaiabdQgaQbqabaGccqGHiiIZcuWGwbGvgaqbamaaBaaaleaacqWGPbqAaeqaaOGafm4DaCNbauaadaWgaaWcbaGaemyAaKgabeaakiabg2da9iabdEha3naaBaaaleaacqWGQbGAaeqaaOGaeyypa0JaemyDauNaemOBa4MaemizaqMaemyzauMaemOzayMaeyikIOTaey4aIqIaemOvay1aaSbaaSqaaiabdQgaQbqabaGccqGHiiIZcuWGwbGvgaqbamaaBaaaleaacqWGPbqAaeqaaOGafm4DaCNbauaadaWgaaWcbaGaemyAaKgabeaakiabg2da9iabdEha3naaBaaaleaacqWGQbGAaeqaaOGaeyiyIKRaemyDauNaemOBa4MaemizaqMaemyzauMaemOzayMaeiykaKIaeiyFa0haaa@9AE5@.

#### Composition of MINs

One of the main characteristics of MINs is that they are modular and enable an incremental construction of models of biological systems. The operation of *composition *of two MINs includes establishing new, composed, sets of species, sites and influences. The species set of the resulting MINs is the *union *of species of the composing MINs, and the new sites set is the union of regulatory site sets of composing MINs. All the information about the interactions in composing systems must be also preserved. That means that a particular attention should be paid on the conversion of influences from composing MINs to the resulting one. If source MINs do not contain common species, there is no transformation to perform; the data from these MINs should be just put together.

##### Definition 10 (Union of MINs)

*If *ℳi=(Ci,ℛi,ℐCℛi,ℐℛCi,ℱi)
 MathType@MTEF@5@5@+=feaafiart1ev1aaatCvAUfKttLearuWrP9MDH5MBPbIqV92AaeXatLxBI9gBaebbnrfifHhDYfgasaacPC6xNi=xH8viVGI8Gi=hEeeu0xXdbba9frFj0xb9qqpG0dXdb9aspeI8k8fiI+fsY=rqGqVepae9pg0db9vqaiVgFr0xfr=xfr=xc9adbaqaaeGacaGaaiaabeqaaeqabiWaaaGcbaWenfgDOvwBHrxAJfwnHbqeg0uy0HwzTfgDPnwy1aaceaGae83mH00aaSbaaSqaaiabdMgaPbqabaGccqGH9aqpcqGGOaakcqWFce=qdaWgaaWcbaGaemyAaKgabeaakiabcYcaSiab=TrisnaaBaaaleaacqWGPbqAaeqaaOGaeiilaWIae8heHKKae8NaXpKae83gHi1aaSbaaSqaaiabdMgaPbqabaGccqGGSaalcqWFqesscqWFBeIucqWFce=qdaWgaaWcbaGaemyAaKgabeaakiabcYcaSiab=ftignaaBaaaleaacqWGPbqAaeqaaOGaeiykaKcaaa@51D9@*for i *= 1, 2 *are MINs, their *union ℳ=ℳ1⊕ℳ2
 MathType@MTEF@5@5@+=feaafiart1ev1aaatCvAUfKttLearuWrP9MDH5MBPbIqV92AaeXatLxBI9gBaebbnrfifHhDYfgasaacPC6xNi=xH8viVGI8Gi=hEeeu0xXdbba9frFj0xb9qqpG0dXdb9aspeI8k8fiI+fsY=rqGqVepae9pg0db9vqaiVgFr0xfr=xfr=xc9adbaqaaeGacaGaaiaabeqaaeqabiWaaaGcbaWenfgDOvwBHrxAJfwnHbqeg0uy0HwzTfgDPnwy1aaceaGae83mH0Kaeyypa0Jae83mH00aaSbaaSqaaiabigdaXaqabaGccqGHvksXcqWFZestdaWgaaWcbaGaeGOmaidabeaaaaa@3E54@*is the MIN such that *ℳ=def{C1∪C2,ℛ1∪ℛ2,ℐCℛ1∪ℐCℛ2,ℐℛC1∪ℐℛC2,ℱ1×U2∪U1×ℱ2}
 MathType@MTEF@5@5@+=feaafiart1ev1aaatCvAUfKttLearuWrP9MDH5MBPbIqV92AaeXatLxBI9gBaebbnrfifHhDYfgasaacPC6xNi=xH8viVGI8Gi=hEeeu0xXdbba9frFj0xb9qqpG0dXdb9aspeI8k8fiI+fsY=rqGqVepae9pg0db9vqaiVgFr0xfr=xfr=xc9adbaqaaeGacaGaaiaabeqaaeqabiWaaaGcbaWenfgDOvwBHrxAJfwnHbqeg0uy0HwzTfgDPnwy1aaceaGae83mH00aaCbiaeaacqGH9aqpaSqabeaacqWGKbazcqWGLbqzcqWGMbGzaaGccqGG7bWEcqWFce=qdaWgaaWcbaGaeGymaedabeaakiabgQIiilab=jq8dnaaBaaaleaacqaIYaGmaeqaaOGaeiilaWIae83gHi1aaSbaaSqaaiabigdaXaqabaGccqGHQicYcqWFBeIudaWgaaWcbaGaeGOmaidabeaakiabcYcaSiab=brijjab=jq8djab=TrisnaaBaaaleaacqaIXaqmaeqaaOGaeyOkIGSae8heHKKae8NaXpKae83gHi1aaSbaaSqaaiabikdaYaqabaGccqGGSaalcqWFqesscqWFBeIucqWFce=qdaWgaaWcbaGaeGymaedabeaakiabgQIiilab=brijjab=Trisjab=jq8dnaaBaaaleaacqaIYaGmaeqaaOGaeiilaWIae8xmHy0aaSbaaSqaaiabigdaXaqabaGccqGHxdaTcqWFueFvdaWgaaWcbaGaeGOmaidabeaakiabgQIiilab=rr8vnaaBaaaleaacqaIXaqmaeqaaOGaey41aqRae8xmHy0aaSbaaSqaaiabikdaYaqabaGccqGG9bqFaaa@7772@, *where *U
 MathType@MTEF@5@5@+=feaafiart1ev1aaatCvAUfKttLearuWrP9MDH5MBPbIqV92AaeXatLxBI9gBaebbnrfifHhDYfgasaacPC6xNi=xH8viVGI8Gi=hEeeu0xXdbba9frFj0xb9qqpG0dXdb9aspeI8k8fiI+fsY=rqGqVepae9pg0db9vqaiVgFr0xfr=xfr=xc9adbaqaaeGacaGaaiaabeqaaeqabiWaaaGcbaWenfgDOvwBHrxAJfwnHbqeg0uy0HwzTfgDPnwy1aaceaGae8hfXxfaaa@376F@_*i *_*is the state of model *ℳ
 MathType@MTEF@5@5@+=feaafiart1ev1aaatCvAUfKttLearuWrP9MDH5MBPbIqV92AaeXatLxBI9gBaebbnrfifHhDYfgasaacPC6xNi=xH8viVGI8Gi=hEeeu0xXdbba9frFj0xb9qqpG0dXdb9aspeI8k8fiI+fsY=rqGqVepae9pg0db9vqaiVgFr0xfr=xfr=xc9adbaqaaeGacaGaaiaabeqaaeqabiWaaaGcbaWenfgDOvwBHrxAJfwnHbqeg0uy0HwzTfgDPnwy1aaceaGae83mH0eaaa@36B6@_*i *_*where all variables have the value undef*.

This means that MIN models can be composed from parts that share the same species or are completely independent. This can be very useful at the first construction stages of biological regulatory networks where the data is incomplete and is not necessarily connected.

**Figure 5:**
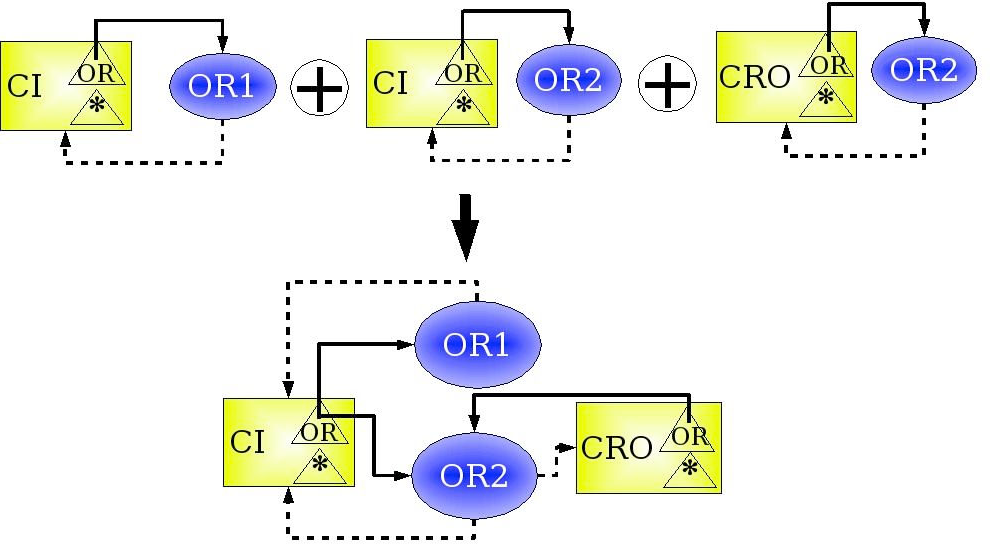
Union and compression of interaction networks. Three networks sharing species and regulatory sites can be combined into one by a composition and compressed by collapsing equivalent species and sites. All existing interactions are preserved.

In case of presence of equivalent regulatory sites or species in the resulting MIN, the union of these sites or species must replace them. In this case the in-fluences between all sites and all the species, which were influencing one another in the source MIN, must be established (see Figure 5). If there are in the source MIN two different influences between the same affinity of a species and the same regulatory site, they must be replaced by only one influence carrying the union of all possible attributes of both connections. In a same way, if there are two different influences from a regulatory site on a given species, it must be replaced by the influence carrying the union of all possible data, using the previously defined operation of simplification of MIN.

### Multivalued logical formalism (MLM): basics

**Figure 6:**
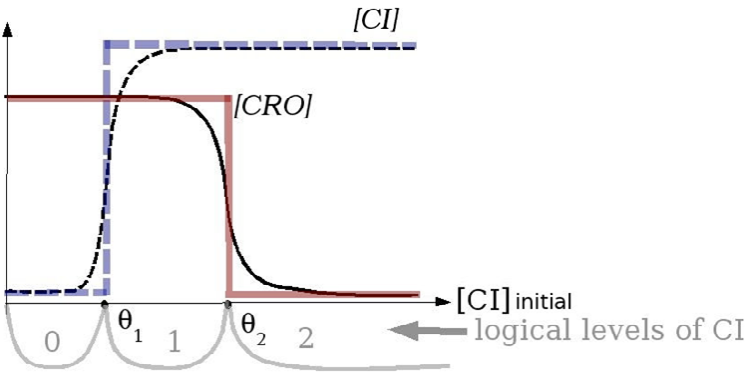
The multivalued logical approximation of the level of activity of biological objects. The axes represent input (abscissa) and output (ordinate) protein concentrations. The dashed thin sigmoid curve represents [*CI*] – the measured concentration of the protein *CI *at the equilibrium point. This curve is approximated by the thick dashed multivalued logical function with the threshold *θ*_1_. The solid curve corresponds to the influence of [CI] on [*CRO*] and its approximation by the multivalued logical function with the threshold *θ*_2_. In this case the activity of the protein *CI *has three logical levels: 0, 1 and 2, indicated in the bottom part and separated by the thresholds.

The multivalued logical approach is designed to express the interdependency between activity levels (often concentrations) of biological objects, e.g., proteins. It applies when this interdependency can be represented by a sigmoidal curve, which is approximated by a multivalued logical function. This function can distinguish between different levels of activity of a biological object, so it may be *multivalued *(see Figure 6). The multivalued logical model (MLM) consists of two parts: a directed graph of interactions and a table of *dynamic parameters*.

The goal of modeling genetic regulatory networks in the multivalued logical formalism [7] is to obtain a state graph representing the behaviour of a biological system from a qualitative point of view. This means that an observable sequence of states of a biological system is represented by a path in the state graph of the model.

The multivalued logical formalism, which has been shown very useful for genetic networks study [11,12], is composed of a directed labeled *regulatory graph *and a table of *dynamic parameters*. The *state *of the regulatory graph, expressed through the labels of its vertices, can evolve according to dynamic parameters. The possible traces of this evolution can be represented in the form of a *state graph*. The nodes of the state graph represent the different states of the system and the arcs of the state graph represent the possible activity modifications of the biological objects.

For dynamic systems with saturation (like genetic regulatory networks) one can approximate the sigmoid curve, representing the level of the activity of a variable as a function of the level of another one, by a multivalued logical function. This approximation is called *logical abstraction *because it allows to distinguish between only two activity states of the system: below the threshold level and above it.

**Figure 7:**
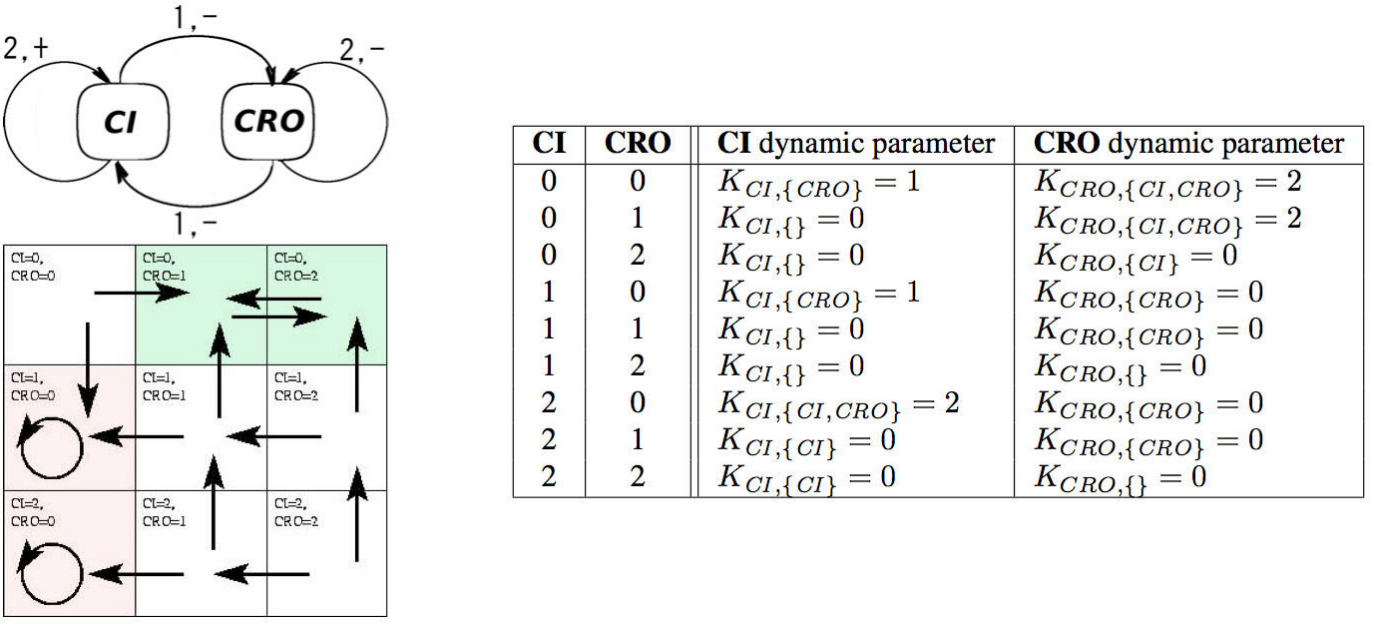
An MLM instance: its regulatory graph (left, top), the corresponding state graph (left, bottom) and the table of its dynamic parameters (right).

The following definition describes an *instance of MLM *as introduced by R. Thomas. It is composed of a regulatory graph (*U*, *E*) and a table *K *of *dynamic parameters *(see Figure 7). Each node *u *of the graph corresponds to a variable with integer values between 0 and the *boundary b*_*u *_of the variable, which drives the topology of the corresponding state graph. The influences between variables in MLM can be positive (*inducing*) or negative (*inhibiting*).

#### Definition 11 (Instance of a Multivalued logical model)

*An *instance *M *of an MLM *of a genetic regulatory network is a pair *(G
 MathType@MTEF@5@5@+=feaafiart1ev1aaatCvAUfKttLearuWrP9MDH5MBPbIqV92AaeXatLxBI9gBaebbnrfifHhDYfgasaacPC6xNi=xH8viVGI8Gi=hEeeu0xXdbba9frFj0xb9qqpG0dXdb9aspeI8k8fiI+fsY=rqGqVepae9pg0db9vqaiVgFr0xfr=xfr=xc9adbaqaaeGacaGaaiaabeqaaeqabiWaaaGcbaWenfgDOvwBHrxAJfwnHbqeg0uy0HwzTfgDPnwy1aaceaGae8NbXFeaaa@3753@, *K*) *where:*

• G
 MathType@MTEF@5@5@+=feaafiart1ev1aaatCvAUfKttLearuWrP9MDH5MBPbIqV92AaeXatLxBI9gBaebbnrfifHhDYfgasaacPC6xNi=xH8viVGI8Gi=hEeeu0xXdbba9frFj0xb9qqpG0dXdb9aspeI8k8fiI+fsY=rqGqVepae9pg0db9vqaiVgFr0xfr=xfr=xc9adbaqaaeGacaGaaiaabeqaaeqabiWaaaGcbaWenfgDOvwBHrxAJfwnHbqeg0uy0HwzTfgDPnwy1aaceaGae8NbXFeaaa@3753@ = (*U*, *E*) *is a labeled directed graph:*

- *each vertex u *∈ *U is called a *variable *of the genetic regulatory network, and is provided with a strictly positive integer b*_*u *_*called the *boundary *of u;*

- *each arc *(*u*_1_, *u*_2_) ∈ *E is labeled by a pair *(*θ*, *ε*) *where θ, called the *threshold, *is an integer between 1 and *bu1
 MathType@MTEF@5@5@+=feaafiart1ev1aaatCvAUfKttLearuWrP9MDH5MBPbIqV92AaeXatLxBI9gBaebbnrfifHhDYfgasaacPC6xNi=xH8viVGI8Gi=hEeeu0xXdbba9frFj0xb9qqpG0dXdb9aspeI8k8fiI+fsY=rqGqVepae9pg0db9vqaiVgFr0xfr=xfr=xc9adbaqaaeGacaGaaiaabeqaaeqabiWaaaGcbaGaemOyai2aaSbaaSqaaiabdwha1naaBaaameaacqaIXaqmaeqaaaWcbeaaaaa@2FE7@, *and ε, called the *sign, *belongs to *{+, -}. *When ε *= +, *u*_1 _*is called an *inducer *of u*_2_. *When ε *= -, *u*_1 _*is called an *inhibitor *of u*_2_. *The set of predecessors of u*_2 _*is denoted *G
 MathType@MTEF@5@5@+=feaafiart1ev1aaatCvAUfKttLearuWrP9MDH5MBPbIqV92AaeXatLxBI9gBaebbnrfifHhDYfgasaacPC6xNi=xH8viVGI8Gi=hEeeu0xXdbba9frFj0xb9qqpG0dXdb9aspeI8k8fiI+fsY=rqGqVepae9pg0db9vqaiVgFr0xfr=xfr=xc9adbaqaaeGacaGaaiaabeqaaeqabiWaaaGcbaWenfgDOvwBHrxAJfwnHbqeg0uy0HwzTfgDPnwy1aaceaGae8NbXFeaaa@3753@^-1^(*u*_2_).

• *K *= {*K*_*u*,*ω *_| *u *∈ *U *∧ *ω *⊆ G
 MathType@MTEF@5@5@+=feaafiart1ev1aaatCvAUfKttLearuWrP9MDH5MBPbIqV92AaeXatLxBI9gBaebbnrfifHhDYfgasaacPC6xNi=xH8viVGI8Gi=hEeeu0xXdbba9frFj0xb9qqpG0dXdb9aspeI8k8fiI+fsY=rqGqVepae9pg0db9vqaiVgFr0xfr=xfr=xc9adbaqaaeGacaGaaiaabeqaaeqabiWaaaGcbaWenfgDOvwBHrxAJfwnHbqeg0uy0HwzTfgDPnwy1aaceaGae8NbXFeaaa@3753@^-1^(*u*)} *is a family of integers such that *0 ≤ *K*_*u*,*ω *_≤ *b*_*u *_*for any variable u and any subset ω of predecessors of u in the graph *G
 MathType@MTEF@5@5@+=feaafiart1ev1aaatCvAUfKttLearuWrP9MDH5MBPbIqV92AaeXatLxBI9gBaebbnrfifHhDYfgasaacPC6xNi=xH8viVGI8Gi=hEeeu0xXdbba9frFj0xb9qqpG0dXdb9aspeI8k8fiI+fsY=rqGqVepae9pg0db9vqaiVgFr0xfr=xfr=xc9adbaqaaeGacaGaaiaabeqaaeqabiWaaaGcbaWenfgDOvwBHrxAJfwnHbqeg0uy0HwzTfgDPnwy1aaceaGae8NbXFeaaa@3753@, *called the *dynamic parameters *of u*.

The dynamics of an MLM instance *M *is defined through the notion of states and transitions. A *state of M *is a *mapping μ *: *U *→ ℕ such that, for any variable *u *∈ *U*, 0 ≤ *μ*(*u*) ≤ *b*_*u*_. The *value μ*(*u*) is then called the *level *of the variable *u*. For example, an MLM instance with two variables *u*_1 _and *u*_2 _with bu1=bu2
 MathType@MTEF@5@5@+=feaafiart1ev1aaatCvAUfKttLearuWrP9MDH5MBPbIqV92AaeXatLxBI9gBaebbnrfifHhDYfgasaacPC6xNi=xH8viVGI8Gi=hEeeu0xXdbba9frFj0xb9qqpG0dXdb9aspeI8k8fiI+fsY=rqGqVepae9pg0db9vqaiVgFr0xfr=xfr=xc9adbaqaaeGacaGaaiaabeqaaeqabiWaaaGcbaGaemOyai2aaSbaaSqaaiabdwha1naaBaaameaacqaIXaqmaeqaaaWcbeaakiabg2da9iabdkgaInaaBaaaleaacqWG1bqDdaWgaaadbaGaeGOmaidabeaaaSqabaaaaa@350D@ = 2 has 9 states corresponding to the following mappings *μ*_1 _= (0, 0), *μ*_2 _= (0, 1), *μ*_3 _= (0, 2),...,*μ*_7 _= (2, 0), *μ*_8 _= (2, 1), *μ*_9 _= (2, 2). In this case the level of variable *u*_2 _in state *μ*_2 _is *μ*_2_(*u*_2_) = 1.

In order to unify the treatment of different influences between variables, the definition of *resources of a variable *is introduced in MLM. The variable *u*_1 _influencing the variable *u*_2 _is a resource in some state if *u*_1 _*helps *the variable *u*_2 _in that state, meaning that *u*_1 _acts to increase the activity level of *u*_2_.

#### Definition 12 (Resources of a Variable)

*Given a state μ and a variable u *∈ *U of a MLM M, the *set of resources *of u is the set ω*_*u*_(*μ*) *containing all the variables u' of M such that:*

• *u' *∈ G
 MathType@MTEF@5@5@+=feaafiart1ev1aaatCvAUfKttLearuWrP9MDH5MBPbIqV92AaeXatLxBI9gBaebbnrfifHhDYfgasaacPC6xNi=xH8viVGI8Gi=hEeeu0xXdbba9frFj0xb9qqpG0dXdb9aspeI8k8fiI+fsY=rqGqVepae9pg0db9vqaiVgFr0xfr=xfr=xc9adbaqaaeGacaGaaiaabeqaaeqabiWaaaGcbaWenfgDOvwBHrxAJfwnHbqeg0uy0HwzTfgDPnwy1aaceaGae8NbXFeaaa@3753@^-1^(*u*) *is a predecessor of u in the underlying directed graph G of M;*

• *the arc *(*u'*, *u*) *is labeled by *(*θ*, *ε*) *and*

- *if ε *= "+" *then μ*(*u'*) ≥ *θ*,

- *if ε *= "-" *then μ*(*u'*) ≤ *θ*.

The set of variables *ω*_*u*_(*μ*) is consequently the subset of G
 MathType@MTEF@5@5@+=feaafiart1ev1aaatCvAUfKttLearuWrP9MDH5MBPbIqV92AaeXatLxBI9gBaebbnrfifHhDYfgasaacPC6xNi=xH8viVGI8Gi=hEeeu0xXdbba9frFj0xb9qqpG0dXdb9aspeI8k8fiI+fsY=rqGqVepae9pg0db9vqaiVgFr0xfr=xfr=xc9adbaqaaeGacaGaaiaabeqaaeqabiWaaaGcbaWenfgDOvwBHrxAJfwnHbqeg0uy0HwzTfgDPnwy1aaceaGae8NbXFeaaa@3753@^-1^(*u*) containing both inducers of *u *whose expression level has reached the threshold and the inhibitors of *u *whose expression level has *not *reached the threshold.

The dynamics of the MLM reflects the dynamics of a "continuous" biological process, so the model variables cannot "skip" values: going from "1" to "3", for example, without passing by the value "2". So, the *multivalued logical function *is introduced to describe the evolution of a variable level in a given system state.

#### Definition 13 (Multivalued Logical Function)

*Given a state μ and a variable u of an instance M of MLM*, the multivalued logical function *κ*_*u*_(*μ*) *is defined as follows:*

• *if μ*(*u*) <Ku,ωu(μ)
 MathType@MTEF@5@5@+=feaafiart1ev1aaatCvAUfKttLearuWrP9MDH5MBPbIqV92AaeXatLxBI9gBaebbnrfifHhDYfgasaacPC6xNi=xH8viVGI8Gi=hEeeu0xXdbba9frFj0xb9qqpG0dXdb9aspeI8k8fiI+fsY=rqGqVepae9pg0db9vqaiVgFr0xfr=xfr=xc9adbaqaaeGacaGaaiaabeqaaeqabiWaaaGcbaGaem4saS0aaSbaaSqaaiabdwha1jabcYcaSGGaciab=L8a3naaBaaameaacqWG1bqDaeqaaSGaeiikaGIae8hVd0MaeiykaKcabeaaaaa@3653@*then κ*_*u*_(*μ*) = *μ*(*u*) + 1

• *if μ*(*u*) = Ku,ωu(μ)
 MathType@MTEF@5@5@+=feaafiart1ev1aaatCvAUfKttLearuWrP9MDH5MBPbIqV92AaeXatLxBI9gBaebbnrfifHhDYfgasaacPC6xNi=xH8viVGI8Gi=hEeeu0xXdbba9frFj0xb9qqpG0dXdb9aspeI8k8fiI+fsY=rqGqVepae9pg0db9vqaiVgFr0xfr=xfr=xc9adbaqaaeGacaGaaiaabeqaaeqabiWaaaGcbaGaem4saS0aaSbaaSqaaiabdwha1jabcYcaSGGaciab=L8a3naaBaaameaacqWG1bqDaeqaaSGaeiikaGIae8hVd0MaeiykaKcabeaaaaa@3653@*then κ*_*u*_(*μ*) = *μ*(*u*)

• *if μ*(*u*) > Ku,ωu(μ)
 MathType@MTEF@5@5@+=feaafiart1ev1aaatCvAUfKttLearuWrP9MDH5MBPbIqV92AaeXatLxBI9gBaebbnrfifHhDYfgasaacPC6xNi=xH8viVGI8Gi=hEeeu0xXdbba9frFj0xb9qqpG0dXdb9aspeI8k8fiI+fsY=rqGqVepae9pg0db9vqaiVgFr0xfr=xfr=xc9adbaqaaeGacaGaaiaabeqaaeqabiWaaaGcbaGaem4saS0aaSbaaSqaaiabdwha1jabcYcaSGGaciab=L8a3naaBaaameaacqWG1bqDaeqaaSGaeiikaGIae8hVd0MaeiykaKcabeaaaaa@3653@*then κ*_*u*_(*μ*) = *μ*(*u*) - 1

The function *κ*_*u *_represents a "step by step" evolution of the expression level of *u *from its current expression level *μ*(*u*) to its dynamic parameters Ku,ωu(μ)
 MathType@MTEF@5@5@+=feaafiart1ev1aaatCvAUfKttLearuWrP9MDH5MBPbIqV92AaeXatLxBI9gBaebbnrfifHhDYfgasaacPC6xNi=xH8viVGI8Gi=hEeeu0xXdbba9frFj0xb9qqpG0dXdb9aspeI8k8fiI+fsY=rqGqVepae9pg0db9vqaiVgFr0xfr=xfr=xc9adbaqaaeGacaGaaiaabeqaaeqabiWaaaGcbaGaem4saS0aaSbaaSqaaiabdwha1jabcYcaSGGaciab=L8a3naaBaaameaacqWG1bqDaeqaaSGaeiikaGIae8hVd0MaeiykaKcabeaaaaa@3653@. The state graph of a MLM is often called *asynchronous *because only one variable can evolve at a time. Then, the evolution of the model can be represented as a *state graph*, where the system can move on a graph of system states according to its multivalued logical function.

#### Definition 14 ("Asynchronous" State Graph)

*The *state graph *of a MLM M is the directed graph *SG
 MathType@MTEF@5@5@+=feaafiart1ev1aaatCvAUfKttLearuWrP9MDH5MBPbIqV92AaeXatLxBI9gBaebbnrfifHhDYfgasaacPC6xNi=xH8viVGI8Gi=hEeeu0xXdbba9frFj0xb9qqpG0dXdb9aspeI8k8fiI+fsY=rqGqVepae9pg0db9vqaiVgFr0xfr=xfr=xc9adbaqaaeGacaGaaiaabeqaaeqabiWaaaGcbaWenfgDOvwBHrxAJfwnHbqeg0uy0HwzTfgDPnwy1aaceaGae8NeXpLae8NbXFeaaa@392E@*whose vertices are all the possible states of M and such that there is an edge from μ to μ' if and only if there exists a variable u satisfying:*

• *μ'*(*u*) = *κ*_*u*_(*μ*) ≠ *μ*(*u*) *where κ*_*u*_(*μ*) *is the multivalued logical function for u;*

• *for any variable u' *≠ *u we have μ'*(*u'*) = *μ*(*u'*).

An arc of the state graph from *μ *to *μ' *is usually denoted as (*μ *→ *μ'*) and is called a *transition*. This is illustrated in Figure 7(right).

## Results

### Translation of a MIN into an MLM

This section presents the translation algorithm of MIN into MLM formalism. It is structured in a following way. First of all, we note that multiple translations of MIN model into MLM formalism are possible, and the impact that it has on the translation algorithm. After that, the translation itself is described, starting with the construction of the MLM regulatory graph topology, then determining the dynamic parameters. At the end, this section contains an example of a translation of a small MIN network into MLM.

The obtained by translation MLM model will be called the *translated network*. As in many cases, the values of all parameters of the MLM model cannot be deduced precisely from the experimental data; the set of all possible parametrisations consistent with biological observations must be considered as a model which can be studied and later be refined by adding other information.

The biological information presented in MIN is much richer than that of an MLM instance, so one MIN can have multiple semantics expressed through a set of MLM instances. In other words, an MLM may be assimilated to the set of its instances. The topology of the regulatory network, as well as the boundaries, will be the same for all instances (deduced from that of MIN). However, dynamic parameters, as well as arc labels can be different since an arc of an MLM regulatory graph may correspond to several arcs of a MIN (one by affinity). As the observable values of a variable of a MIN are partially ordered (see Definition 1), the different ways of enumerating values of *u *(topological sort) will be considered as yielding different instances of the MLM. So, in the following, we will consider every combinations of possible parameters as one instance of MLM, and the translation procedure of MIN into MLM will give all these possible parameters that can be deduced from MIN data.

Now, let us introduce the construction of the MLM regulatory graph from the MIN model. First, the *translated variables *of the MLM must be defined. They are obtained from the species of the MIN, keeping only one (arbitrarily chosen) name and providing it with a boundary corresponding to the number of observable values of the MIN variable. Unless two species share a same name, due to unfortunate choices in independent sources; we shall assume it is always possible to choose those names in such a way that no two different nodes have the same name.

#### Definition 15 (Translated variables of a MIN)

*Let C *∈ V
 MathType@MTEF@5@5@+=feaafiart1ev1aaatCvAUfKttLearuWrP9MDH5MBPbIqV92AaeXatLxBI9gBaebbnrfifHhDYfgasaacPC6xNi=xH8viVGI8Gi=hEeeu0xXdbba9frFj0xb9qqpG0dXdb9aspeI8k8fiI+fsY=rqGqVepae9pg0db9vqaiVgFr0xfr=xfr=xc9adbaqaaeGacaGaaiaabeqaaeqabiWaaaGcbaWenfgDOvwBHrxAJfwnHbqeg0uy0HwzTfgDPnwy1aaceaGae8xfXBfaaa@3771@*be a chemical species of the MIN *ℳ
 MathType@MTEF@5@5@+=feaafiart1ev1aaatCvAUfKttLearuWrP9MDH5MBPbIqV92AaeXatLxBI9gBaebbnrfifHhDYfgasaacPC6xNi=xH8viVGI8Gi=hEeeu0xXdbba9frFj0xb9qqpG0dXdb9aspeI8k8fiI+fsY=rqGqVepae9pg0db9vqaiVgFr0xfr=xfr=xc9adbaqaaeGacaGaaiaabeqaaeqabiWaaaGcbaWenfgDOvwBHrxAJfwnHbqeg0uy0HwzTfgDPnwy1aaceaGae83mH0eaaa@36B6@, *let *|*W*_*C*_| *be the number of different observable values of C and N *∈ *N*_*C *_*be a name of C. The *translation *of C is a vertex u *∈ *U labeled with N and provided with a boundary b*_*u *_= |*W*_*C*_|. *The species C is then called the *original species *of u*.

**Figure 8:**
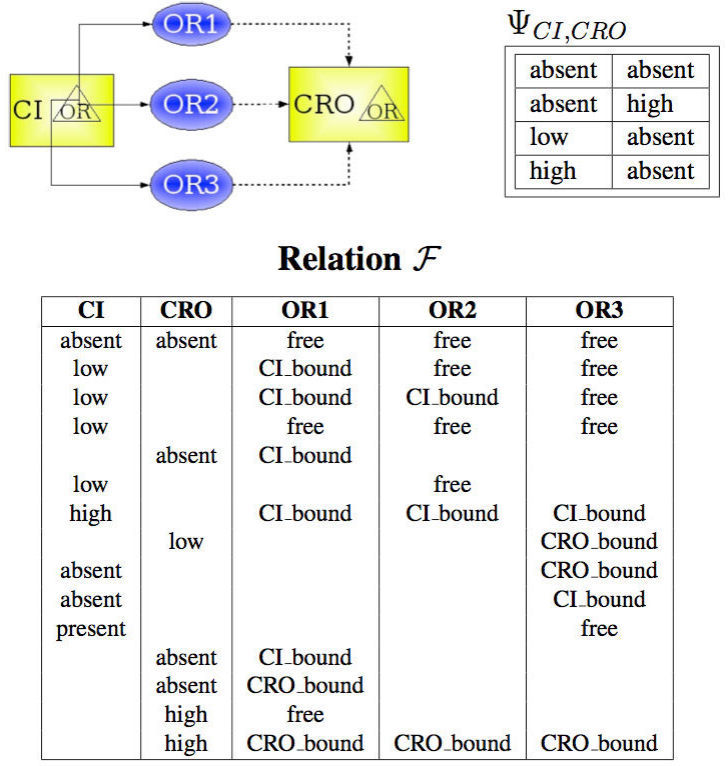
Translation of dynamic information from a MIN to an MLM model. **Top, Left **The species *CI *regulates the species *CRO *through the sites *OR*1, *OR*2 and *OR*3. **Top, Right **The relation Ψ_*CI*,*CRO *_comprises three lines characterizing the regulation of *CRO *by *CI *through the regulatory site *OR*1. **Bottom **The relation ℱ
 MathType@MTEF@5@5@+=feaafiart1ev1aaatCvAUfKttLearuWrP9MDH5MBPbIqV92AaeXatLxBI9gBaebbnrfifHhDYfgasaacPC6xNi=xH8viVGI8Gi=hEeeu0xXdbba9frFj0xb9qqpG0dXdb9aspeI8k8fiI+fsY=rqGqVepae9pg0db9vqaiVgFr0xfr=xfr=xc9adbaqaaeGacaGaaiaabeqaaeqabiWaaaGcbaWenfgDOvwBHrxAJfwnHbqeg0uy0HwzTfgDPnwy1aaceaGae8xmHyeaaa@36AD@ shows *undef *values as white spaces.

The arcs of the regulatory graph of the MLM are deduced from the MIN structure in the following way: there is an arc between the translated variables *u*_1 _and *u*_2 _iff there is a pair (*ICR*, *IRC*) in MIN such that *R*_*ICR *_= *R*_*IRC*_, and *C*_*ICR *_and *C*_*IRC *_are the original species of variables *u*_1 _and *u*_2_, respectively (see Figure 8).

The MLM regulatory graph is not complete yet, as we need to find the arc labels. These labels depend on the observed values of MIN variables. The information on the possible combinations of observed values of variables is contained in the relation ℱ
 MathType@MTEF@5@5@+=feaafiart1ev1aaatCvAUfKttLearuWrP9MDH5MBPbIqV92AaeXatLxBI9gBaebbnrfifHhDYfgasaacPC6xNi=xH8viVGI8Gi=hEeeu0xXdbba9frFj0xb9qqpG0dXdb9aspeI8k8fiI+fsY=rqGqVepae9pg0db9vqaiVgFr0xfr=xfr=xc9adbaqaaeGacaGaaiaabeqaaeqabiWaaaGcbaWenfgDOvwBHrxAJfwnHbqeg0uy0HwzTfgDPnwy1aaceaGae8xmHyeaaa@36AD@. The same type of knowledge enables us to determine also the dynamic parameters of the MLM model. However, the influences are defined in MIN between chemical species and regulatory sites, but the MLM model encompasses the regulatory sites inside the variables representing the species, as shown in the previous definition. Thus, we need to reconstruct the parameters of influ-ences of species on species from ℱ
 MathType@MTEF@5@5@+=feaafiart1ev1aaatCvAUfKttLearuWrP9MDH5MBPbIqV92AaeXatLxBI9gBaebbnrfifHhDYfgasaacPC6xNi=xH8viVGI8Gi=hEeeu0xXdbba9frFj0xb9qqpG0dXdb9aspeI8k8fiI+fsY=rqGqVepae9pg0db9vqaiVgFr0xfr=xfr=xc9adbaqaaeGacaGaaiaabeqaaeqabiWaaaGcbaWenfgDOvwBHrxAJfwnHbqeg0uy0HwzTfgDPnwy1aaceaGae8xmHyeaaa@36AD@ and the MIN topology.

In order to find the arc labels of the translated regulatory graph and the corresponding dynamic parameters *K*, we introduce the relation Ψ_*ik *_between values of the species *C*_*i *_and the species *C*_*k*_, called *interspecies regulation relation*. This relation is defined if there is a site *R*_*j *_such that there is an *ICR*_*ij *_with (*Affinity*, *a*) ∈ PICRij
 MathType@MTEF@5@5@+=feaafiart1ev1aaatCvAUfKttLearuWrP9MDH5MBPbIqV92AaeXatLxBI9gBaebbnrfifHhDYfgasaacPC6xNi=xH8viVGI8Gi=hEeeu0xXdbba9frFj0xb9qqpG0dXdb9aspeI8k8fiI+fsY=rqGqVepae9pg0db9vqaiVgFr0xfr=xfr=xc9adbaqaaeGacaGaaiaabeqaaeqabiWaaaGcbaGaemiuaa1aaSbaaSqaaiabdMeajjabdoeadjabdkfasnaaBaaameaacqWGPbqAcqWGQbGAaeqaaaWcbeaaaaa@336F@ and (*Affinity*, *a*, 0) ∈ PCi
 MathType@MTEF@5@5@+=feaafiart1ev1aaatCvAUfKttLearuWrP9MDH5MBPbIqV92AaeXatLxBI9gBaebbnrfifHhDYfgasaacPC6xNi=xH8viVGI8Gi=hEeeu0xXdbba9frFj0xb9qqpG0dXdb9aspeI8k8fiI+fsY=rqGqVepae9pg0db9vqaiVgFr0xfr=xfr=xc9adbaqaaeGacaGaaiaabeqaaeqabiWaaaGcbaGaemiuaa1aaSbaaSqaaiabdoeadnaaBaaameaacqWGPbqAaeqaaaWcbeaaaaa@2FCA@ and there is an *IRC*_*jk *_in the MIN, i.e., the species *C*_*i *_*regulates *the species *C*_*j *_through the site *R*_*j*_. For example, on Figure 8, the species *CI *regulates the species *CRO *through the sites *OR*1, *OR*2 and *OR*3.

In order to translate the information about the dynamics of the biological system, contained in ℱ
 MathType@MTEF@5@5@+=feaafiart1ev1aaatCvAUfKttLearuWrP9MDH5MBPbIqV92AaeXatLxBI9gBaebbnrfifHhDYfgasaacPC6xNi=xH8viVGI8Gi=hEeeu0xXdbba9frFj0xb9qqpG0dXdb9aspeI8k8fiI+fsY=rqGqVepae9pg0db9vqaiVgFr0xfr=xfr=xc9adbaqaaeGacaGaaiaabeqaaeqabiWaaaGcbaWenfgDOvwBHrxAJfwnHbqeg0uy0HwzTfgDPnwy1aaceaGae8xmHyeaaa@36AD@, we need to define the *choice *operation *σ*, which we will call a *selection*, as presented in following definition. For each pair of variables *V*_*i*_, *V*_*j*_, the selection σVi,Vj(ℱ)
 MathType@MTEF@5@5@+=feaafiart1ev1aaatCvAUfKttLearuWrP9MDH5MBPbIqV92AaeXatLxBI9gBaebbnrfifHhDYfgasaacPC6xNi=xH8viVGI8Gi=hEeeu0xXdbba9frFj0xb9qqpG0dXdb9aspeI8k8fiI+fsY=rqGqVepae9pg0db9vqaiVgFr0xfr=xfr=xc9adbaqaaeGacaGaaiaabeqaaeqabiWaaaGcbaacciGae83Wdm3aaSbaaSqaaiabdAfawnaaBaaameaacqWGPbqAaeqaaSGaeiilaWIaemOvay1aaSbaaWqaaiabdQgaQbqabaaaleqaaOGaeiikaGYenfgDOvwBHrxAJfwnHbqeg0uy0HwzTfgDPnwy1aaceaGae4xmHyKaeiykaKcaaa@40D0@ returns the observed system states in which both values of variables *i *and *j *were measured.

#### Definition 16 (Selection of observed states for a pair of MIN variables)

*The *selection *of observed states *ℱ
 MathType@MTEF@5@5@+=feaafiart1ev1aaatCvAUfKttLearuWrP9MDH5MBPbIqV92AaeXatLxBI9gBaebbnrfifHhDYfgasaacPC6xNi=xH8viVGI8Gi=hEeeu0xXdbba9frFj0xb9qqpG0dXdb9aspeI8k8fiI+fsY=rqGqVepae9pg0db9vqaiVgFr0xfr=xfr=xc9adbaqaaeGacaGaaiaabeqaaeqabiWaaaGcbaWenfgDOvwBHrxAJfwnHbqeg0uy0HwzTfgDPnwy1aaceaGae8xmHyeaaa@36AD@*of a biological system *ℳ
 MathType@MTEF@5@5@+=feaafiart1ev1aaatCvAUfKttLearuWrP9MDH5MBPbIqV92AaeXatLxBI9gBaebbnrfifHhDYfgasaacPC6xNi=xH8viVGI8Gi=hEeeu0xXdbba9frFj0xb9qqpG0dXdb9aspeI8k8fiI+fsY=rqGqVepae9pg0db9vqaiVgFr0xfr=xfr=xc9adbaqaaeGacaGaaiaabeqaaeqabiWaaaGcbaWenfgDOvwBHrxAJfwnHbqeg0uy0HwzTfgDPnwy1aaceaGae83mH0eaaa@36B6@*for a pair of variables V*_*i*_, *V*_*j *_*is the subset *σVi,Vj⊆ℱ
 MathType@MTEF@5@5@+=feaafiart1ev1aaatCvAUfKttLearuWrP9MDH5MBPbIqV92AaeXatLxBI9gBaebbnrfifHhDYfgasaacPC6xNi=xH8viVGI8Gi=hEeeu0xXdbba9frFj0xb9qqpG0dXdb9aspeI8k8fiI+fsY=rqGqVepae9pg0db9vqaiVgFr0xfr=xfr=xc9adbaqaaeGacaGaaiaabeqaaeqabiWaaaGcbaacciGae83Wdm3aaSbaaSqaaiabdAfawnaaBaaameaacqWGPbqAaeqaaSGaeiilaWIaemOvay1aaSbaaWqaaiabdQgaQbqabaaaleqaaOGaeyOHI08enfgDOvwBHrxAJfwnHbqeg0uy0HwzTfgDPnwy1aaceaGae4xmHyeaaa@411F@*such that *ω∈σVi,Vj
 MathType@MTEF@5@5@+=feaafiart1ev1aaatCvAUfKttLearuWrP9MDH5MBPbIqV92AaeXatLxBI9gBaebbnrfifHhDYfgasaacPC6xNi=xH8viVGI8Gi=hEeeu0xXdbba9frFj0xb9qqpG0dXdb9aspeI8k8fiI+fsY=rqGqVepae9pg0db9vqaiVgFr0xfr=xfr=xc9adbaqaaeGacaGaaiaabeqaaeqabiWaaaGcbaacciGae8xYdCNaeyicI4Sae83Wdm3aaSbaaSqaaiabdAfawnaaBaaameaacqWGPbqAaeqaaSGaeiilaWIaemOvay1aaSbaaWqaaiabdQgaQbqabaaaleqaaaaa@3787@*if and only if ω*(*V*_*i*_) *and ω*(*V*_*j*_) *are both defined*.

The selection will be used in the next definition in order to formally define the *interspecies regulation relation *Ψ_*i*,*k*_, which links the values of species *i *and *k *which could be observed experimentally at the same time. This relation lists the values coming from ℱ
 MathType@MTEF@5@5@+=feaafiart1ev1aaatCvAUfKttLearuWrP9MDH5MBPbIqV92AaeXatLxBI9gBaebbnrfifHhDYfgasaacPC6xNi=xH8viVGI8Gi=hEeeu0xXdbba9frFj0xb9qqpG0dXdb9aspeI8k8fiI+fsY=rqGqVepae9pg0db9vqaiVgFr0xfr=xfr=xc9adbaqaaeGacaGaaiaabeqaaeqabiWaaaGcbaWenfgDOvwBHrxAJfwnHbqeg0uy0HwzTfgDPnwy1aaceaGae8xmHyeaaa@36AD@ lines where states were observed for species *i*, species *k *and the regulatory site *R*, influenced by *i *and influencing *k*. That means that the interaction of species *i *and *k *is transmitted by the regulatory site *R*.

#### Definition 17 (Interspecies regulation relation)

*An *interspecies regulation relation Ψi,k⊆WCi×WCk
 MathType@MTEF@5@5@+=feaafiart1ev1aaatCvAUfKttLearuWrP9MDH5MBPbIqV92AaeXatLxBI9gBaebbnrfifHhDYfgasaacPC6xNi=xH8viVGI8Gi=hEeeu0xXdbba9frFj0xb9qqpG0dXdb9aspeI8k8fiI+fsY=rqGqVepae9pg0db9vqaiVgFr0xfr=xfr=xc9adbaqaaeGacaGaaiaabeqaaeqabiWaaaGcbaGaeuiQdK1aaSbaaSqaaiabdMgaPjabcYcaSiabdUgaRbqabaGccqGHgksZcqWGxbWvdaWgaaWcbaGaem4qam0aaSbaaWqaaiabdMgaPbqabaaaleqaaOGaey41aqRaem4vaC1aaSbaaSqaaiabdoeadnaaBaaameaacqWGRbWAaeqaaaWcbeaaaaa@3D62@*is a relation between values of the species C*_*i *_*and C*_*k *_*of a MIN *ℳ
 MathType@MTEF@5@5@+=feaafiart1ev1aaatCvAUfKttLearuWrP9MDH5MBPbIqV92AaeXatLxBI9gBaebbnrfifHhDYfgasaacPC6xNi=xH8viVGI8Gi=hEeeu0xXdbba9frFj0xb9qqpG0dXdb9aspeI8k8fiI+fsY=rqGqVepae9pg0db9vqaiVgFr0xfr=xfr=xc9adbaqaaeGacaGaaiaabeqaaeqabiWaaaGcbaWenfgDOvwBHrxAJfwnHbqeg0uy0HwzTfgDPnwy1aaceaGae83mH0eaaa@36B6@, *defined when the species C*_*i *_*regulates the species *Ck:Ψik=def{(w1,w2)|(Ci,R,P,L)∈ℐCℛ,(R,Ck,P,L)∈ℐℛC,ω1,ω2∈ℱ:w1=ω1(Ci),ω1(R)=ω2(R),ω2(Ck)=w2}
 MathType@MTEF@5@5@+=feaafiart1ev1aaatCvAUfKttLearuWrP9MDH5MBPbIqV92AaeXatLxBI9gBaebbnrfifHhDYfgasaacPC6xNi=xH8viVGI8Gi=hEeeu0xXdbba9frFj0xb9qqpG0dXdb9aspeI8k8fiI+fsY=rqGqVepae9pg0db9vqaiVgFr0xfr=xfr=xc9adbaqaaeGacaGaaiaabeqaaeqabiWaaaGcbaGaem4qam0aaSbaaSqaaiabdUgaRbqabaGccqGG6aGocqqHOoqwdaWgaaWcbaGaemyAaKMaem4AaSgabeaakmaaxacabaGaeyypa0daleqabaGaemizaqMaemyzauMaemOzaygaaOGaei4EaSNaeiikaGIaem4DaC3aaSbaaSqaaiabigdaXaqabaGccqGGSaalcqWG3bWDdaWgaaWcbaGaeGOmaidabeaakiabcMcaPiabcYha8jabcIcaOiabdoeadnaaBaaaleaacqWGPbqAaeqaaOGaeiilaWIaemOuaiLaeiilaWIaemiuaaLaeiilaWIaemitaWKaeiykaKIaeyicI48enfgDOvwBHrxAJfwnHbqeg0uy0HwzTfgDPnwy1aaceaGae8heHKKae8NaXpKae83gHiLaeiilaWIaeiikaGIaemOuaiLaeiilaWIaem4qam0aaSbaaSqaaiabdUgaRbqabaGccqGGSaalcqWGqbaucqGGSaalcqWGmbatcqGGPaqkcqGHiiIZcqWFqesscqWFBeIucqWFce=qcqGGSaaliiGacqGFjpWDdaWgaaWcbaGaeGymaedabeaakiabcYcaSiab+L8a3naaBaaaleaacqaIYaGmaeqaaOGaeyicI4Sae8xmHyKaeiOoaOJaem4DaC3aaSbaaSqaaiabigdaXaqabaGccqGH9aqpcqGFjpWDdaWgaaWcbaGaeGymaedabeaakiabcIcaOiabdoeadnaaBaaaleaacqWGPbqAaeqaaOGaeiykaKIaeiilaWIae4xYdC3aaSbaaSqaaiabigdaXaqabaGccqGGOaakcqWGsbGucqGGPaqkcqGH9aqpcqGFjpWDdaWgaaWcbaGaeGOmaidabeaakiabcIcaOiabdkfasjabcMcaPiabcYcaSiab+L8a3naaBaaaleaacqaIYaGmaeqaaOGaeiikaGIaem4qam0aaSbaaSqaaiabdUgaRbqabaGccqGGPaqkcqGH9aqpcqWG3bWDdaWgaaWcbaGaeGOmaidabeaakiabc2ha9baa@9F18@.

Thus, the Ψ relation lists the pairs of values (*w*_*i*_, *w*_*k*_) of species *C*_*i *_and *C*_*k *_such that the value *w*_*i *_of the species *C*_*i *_and the value *w*_*k *_of the species *C*_*k *_where observed simultaneously or when the regulatory site linking them was in the same state (for an example see Figure 8).

The next definition uses the interspecies regulation relation in order to add the missing labels on the arcs of MLM regulatory graph, translated from MIN. The observed values, returned by the interspecies regulation relation, are sorted by the first value, and then the algorithm tries to fit them to a sigmoid curve, an ascendant or a descendant one. If such fitting is possible, the algorithm tries to determine the threshold for this sigmoid curve. The first fact is translated by the sign, "+" or "-", in the arc label. The threshold value is also mentioned on the corresponding arc, when found.

#### Definition 18 (Translated regulatory graph)

*If *ℳ
 MathType@MTEF@5@5@+=feaafiart1ev1aaatCvAUfKttLearuWrP9MDH5MBPbIqV92AaeXatLxBI9gBaebbnrfifHhDYfgasaacPC6xNi=xH8viVGI8Gi=hEeeu0xXdbba9frFj0xb9qqpG0dXdb9aspeI8k8fiI+fsY=rqGqVepae9pg0db9vqaiVgFr0xfr=xfr=xc9adbaqaaeGacaGaaiaabeqaaeqabiWaaaGcbaWenfgDOvwBHrxAJfwnHbqeg0uy0HwzTfgDPnwy1aaceaGae83mH0eaaa@36B6@ = (V,ℐCℛ,ℐℛC,ℱ,ℒ
 MathType@MTEF@5@5@+=feaafiart1ev1aaatCvAUfKttLearuWrP9MDH5MBPbIqV92AaeXatLxBI9gBaebbnrfifHhDYfgasaacPC6xNi=xH8viVGI8Gi=hEeeu0xXdbba9frFj0xb9qqpG0dXdb9aspeI8k8fiI+fsY=rqGqVepae9pg0db9vqaiVgFr0xfr=xfr=xc9adbaqaaeGacaGaaiaabeqaaeqabiWaaaGcbaWenfgDOvwBHrxAJfwnHbqeg0uy0HwzTfgDPnwy1aaceaGae8xfXBLaeiilaWIae8heHKKae8NaXpKae83gHiLaeiilaWIae8heHKKae83gHiLae8NaXpKaeiilaWIae8xmHyKaeiilaWIae8NeHWeaaa@44AC@) *is a MIN with *V=C∪ℛ
 MathType@MTEF@5@5@+=feaafiart1ev1aaatCvAUfKttLearuWrP9MDH5MBPbIqV92AaeXatLxBI9gBaebbnrfifHhDYfgasaacPC6xNi=xH8viVGI8Gi=hEeeu0xXdbba9frFj0xb9qqpG0dXdb9aspeI8k8fiI+fsY=rqGqVepae9pg0db9vqaiVgFr0xfr=xfr=xc9adbaqaaeGacaGaaiaabeqaaeqabiWaaaGcbaWenfgDOvwBHrxAJfwnHbqeg0uy0HwzTfgDPnwy1aaceaGae8xfXBLaeyypa0Jae8NaXpKaeyOkIGSae83gHifaaa@3CE5@, *its *translated regulatory graph G
 MathType@MTEF@5@5@+=feaafiart1ev1aaatCvAUfKttLearuWrP9MDH5MBPbIqV92AaeXatLxBI9gBaebbnrfifHhDYfgasaacPC6xNi=xH8viVGI8Gi=hEeeu0xXdbba9frFj0xb9qqpG0dXdb9aspeI8k8fiI+fsY=rqGqVepae9pg0db9vqaiVgFr0xfr=xfr=xc9adbaqaaeGacaGaaiaabeqaaeqabiWaaaGcbaWenfgDOvwBHrxAJfwnHbqeg0uy0HwzTfgDPnwy1aaceaGae8NbXFeaaa@3753@ = (*U*, ℰ
 MathType@MTEF@5@5@+=feaafiart1ev1aaatCvAUfKttLearuWrP9MDH5MBPbIqV92AaeXatLxBI9gBaebbnrfifHhDYfgasaacPC6xNi=xH8viVGI8Gi=hEeeu0xXdbba9frFj0xb9qqpG0dXdb9aspeI8k8fiI+fsY=rqGqVepae9pg0db9vqaiVgFr0xfr=xfr=xc9adbaqaaeGacaGaaiaabeqaaeqabiWaaaGcbaWenfgDOvwBHrxAJfwnHbqeg0uy0HwzTfgDPnwy1aaceaGae8hmHueaaa@36AB@) *(representing a set of genetic regulatory graphs) is a directed graph where:*

• *U is a set of translated variables of *ℳ
 MathType@MTEF@5@5@+=feaafiart1ev1aaatCvAUfKttLearuWrP9MDH5MBPbIqV92AaeXatLxBI9gBaebbnrfifHhDYfgasaacPC6xNi=xH8viVGI8Gi=hEeeu0xXdbba9frFj0xb9qqpG0dXdb9aspeI8k8fiI+fsY=rqGqVepae9pg0db9vqaiVgFr0xfr=xfr=xc9adbaqaaeGacaGaaiaabeqaaeqabiWaaaGcbaWenfgDOvwBHrxAJfwnHbqeg0uy0HwzTfgDPnwy1aaceaGae83mH0eaaa@36B6@;

• ℰ
 MathType@MTEF@5@5@+=feaafiart1ev1aaatCvAUfKttLearuWrP9MDH5MBPbIqV92AaeXatLxBI9gBaebbnrfifHhDYfgasaacPC6xNi=xH8viVGI8Gi=hEeeu0xXdbba9frFj0xb9qqpG0dXdb9aspeI8k8fiI+fsY=rqGqVepae9pg0db9vqaiVgFr0xfr=xfr=xc9adbaqaaeGacaGaaiaabeqaaeqabiWaaaGcbaWenfgDOvwBHrxAJfwnHbqeg0uy0HwzTfgDPnwy1aaceaGae8hmHueaaa@36AB@*is the set of arcs *(*u*_1_, *u*_2_) *between variables of U such that:*

- (*u*_1_, *u*_2_) ∈ ℰ
 MathType@MTEF@5@5@+=feaafiart1ev1aaatCvAUfKttLearuWrP9MDH5MBPbIqV92AaeXatLxBI9gBaebbnrfifHhDYfgasaacPC6xNi=xH8viVGI8Gi=hEeeu0xXdbba9frFj0xb9qqpG0dXdb9aspeI8k8fiI+fsY=rqGqVepae9pg0db9vqaiVgFr0xfr=xfr=xc9adbaqaaeGacaGaaiaabeqaaeqabiWaaaGcbaWenfgDOvwBHrxAJfwnHbqeg0uy0HwzTfgDPnwy1aaceaGae8hmHueaaa@36AB@*if u*_*i *_*is a translated variable of C*_*i *_∈ C
 MathType@MTEF@5@5@+=feaafiart1ev1aaatCvAUfKttLearuWrP9MDH5MBPbIqV92AaeXatLxBI9gBaebbnrfifHhDYfgasaacPC6xNi=xH8viVGI8Gi=hEeeu0xXdbba9frFj0xb9qqpG0dXdb9aspeI8k8fiI+fsY=rqGqVepae9pg0db9vqaiVgFr0xfr=xfr=xc9adbaqaaeGacaGaaiaabeqaaeqabiWaaaGcbaWenfgDOvwBHrxAJfwnHbqeg0uy0HwzTfgDPnwy1aaceaGae8NaXpeaaa@374B@, *i *= 1, 2 *and *∃*ICR *∈ ℐCℛ
 MathType@MTEF@5@5@+=feaafiart1ev1aaatCvAUfKttLearuWrP9MDH5MBPbIqV92AaeXatLxBI9gBaebbnrfifHhDYfgasaacPC6xNi=xH8viVGI8Gi=hEeeu0xXdbba9frFj0xb9qqpG0dXdb9aspeI8k8fiI+fsY=rqGqVepae9pg0db9vqaiVgFr0xfr=xfr=xc9adbaqaaeGacaGaaiaabeqaaeqabiWaaaGcbaWenfgDOvwBHrxAJfwnHbqeg0uy0HwzTfgDPnwy1aaceaGae8heHKKae8NaXpKae83gHifaaa@395D@, ∃*IRC *∈ ℐℛC
 MathType@MTEF@5@5@+=feaafiart1ev1aaatCvAUfKttLearuWrP9MDH5MBPbIqV92AaeXatLxBI9gBaebbnrfifHhDYfgasaacPC6xNi=xH8viVGI8Gi=hEeeu0xXdbba9frFj0xb9qqpG0dXdb9aspeI8k8fiI+fsY=rqGqVepae9pg0db9vqaiVgFr0xfr=xfr=xc9adbaqaaeGacaGaaiaabeqaaeqabiWaaaGcbaWenfgDOvwBHrxAJfwnHbqeg0uy0HwzTfgDPnwy1aaceaGae8heHKKae83gHiLae8NaXpeaaa@395D@*such that C*_*ICR *_= *C*_1_, *R*_*ICR *_= *R *= *R*_*IRC *_*and C*_*IRC *_= *C*_2_. *For each pair *(*ICR*, *IRC*) *satisfying these conditions we will use the notation *(*ICR *+ *IRC*) ∈ (*u*_1_, *u*_2_).

- *the arc *(*u*_1_, *u*_2_) *is labeled with a set of pairs *(*θ*, *ε*) *such that:*

* *if *∃wi∈WCi
 MathType@MTEF@5@5@+=feaafiart1ev1aaatCvAUfKttLearuWrP9MDH5MBPbIqV92AaeXatLxBI9gBaebbnrfifHhDYfgasaacPC6xNi=xH8viVGI8Gi=hEeeu0xXdbba9frFj0xb9qqpG0dXdb9aspeI8k8fiI+fsY=rqGqVepae9pg0db9vqaiVgFr0xfr=xfr=xc9adbaqaaeGacaGaaiaabeqaaeqabiWaaaGcbaGaey4aIqIaem4DaC3aaSbaaSqaaiabdMgaPbqabaGccqGHiiIZcqWGxbWvdaWgaaWcbaGaem4qam0aaSbaaWqaaiabdMgaPbqabaaaleqaaaaa@3539@, *i *= 1, 2, (*w*_1_, *w*_2_) ∈ Ψ_1,2 _*such that: *∃Ψ′1,2⊆Ψ1,2:(w1,w2)∈Ψ′1,2
 MathType@MTEF@5@5@+=feaafiart1ev1aaatCvAUfKttLearuWrP9MDH5MBPbIqV92AaeXatLxBI9gBaebbnrfifHhDYfgasaacPC6xNi=xH8viVGI8Gi=hEeeu0xXdbba9frFj0xb9qqpG0dXdb9aspeI8k8fiI+fsY=rqGqVepae9pg0db9vqaiVgFr0xfr=xfr=xc9adbaqaaeGacaGaaiaabeqaaeqabiWaaaGcbaGaey4aIqIafuiQdKLbauaadaWgaaWcbaGaeGymaeJaeiilaWIaeGOmaidabeaakiabgAOinlabfI6aznaaBaaaleaacqaIXaqmcqGGSaalcqaIYaGmaeqaaOGaeiOoaOJaeiikaGIaem4DaC3aaSbaaSqaaiabigdaXaqabaGccqGGSaalcqWG3bWDdaWgaaWcbaGaeGOmaidabeaakiabcMcaPiabgIGiolqbfI6azzaafaWaaSbaaSqaaiabigdaXiabcYcaSiabikdaYaqabaaaaa@469A@*and *∀(w′1,w′2)∈Ψ′1,2
 MathType@MTEF@5@5@+=feaafiart1ev1aaatCvAUfKttLearuWrP9MDH5MBPbIqV92AaeXatLxBI9gBaebbnrfifHhDYfgasaacPC6xNi=xH8viVGI8Gi=hEeeu0xXdbba9frFj0xb9qqpG0dXdb9aspeI8k8fiI+fsY=rqGqVepae9pg0db9vqaiVgFr0xfr=xfr=xc9adbaqaaeGacaGaaiaabeqaaeqabiWaaaGcbaGaeyiaIiIaeiikaGIafm4DaCNbauaadaWgaaWcbaGaeGymaedabeaakiabcYcaSiqbdEha3zaafaWaaSbaaSqaaiabikdaYaqabaGccqGGPaqkcqGHiiIZcuqHOoqwgaqbamaaBaaaleaacqaIXaqmcqGGSaalcqaIYaGmaeqaaaaa@3A96@, *if *w′1≼C1w1⇒w′2≼C2w2
 MathType@MTEF@5@5@+=feaafiart1ev1aaatCvAUfKttLearuWrP9MDH5MBPbIqV92AaeXatLxBI9gBaebbnrfifHhDYfgasaacPC6xNi=xH8viVGI8Gi=hEeeu0xXdbba9frFj0xb9qqpG0dXdb9aspeI8k8fiI+fsY=rqGqVepae9pg0db9vqaiVgFr0xfr=xfr=xc9adbaqaaeGacaGaaiaabeqaaeqabiWaaaGcbaGafm4DaCNbauaadaWgaaWcbaGaeGymaedabeaatuuDJXwAK1uy0HMmaeHbfv3ySLgzG0uy0HgiuD3BaGabaOGae8hFIa7aaSbaaSqaaiabboeadnaaBaaameaacqqGXaqmaeqaaaWcbeaakiabdEha3naaBaaaleaacqaIXaqmaeqaaOGaeyO0H4Tafm4DaCNbauaadaWgaaWcbaGaeGOmaidabeaakiab=XNiWoaaBaaaleaacqqGdbWqdaWgaaadbaGaeeOmaidabeaaaSqabaGccqWG3bWDdaWgaaWcbaGaeGOmaidabeaaaaa@4AC8@*and if *w1≼C1w′1⇒w2≼C2w′2
 MathType@MTEF@5@5@+=feaafiart1ev1aaatCvAUfKttLearuWrP9MDH5MBPbIqV92AaeXatLxBI9gBaebbnrfifHhDYfgasaacPC6xNi=xH8viVGI8Gi=hEeeu0xXdbba9frFj0xb9qqpG0dXdb9aspeI8k8fiI+fsY=rqGqVepae9pg0db9vqaiVgFr0xfr=xfr=xc9adbaqaaeGacaGaaiaabeqaaeqabiWaaaGcbaGaem4DaC3aaSbaaSqaaiabigdaXaqabaWefv3ySLgznfgDOjdaryqr1ngBPrginfgDObcv39gaiqaakiab=XNiWoaaBaaaleaacqqGdbWqdaWgaaadbaGaeeymaedabeaaaSqabaGccuWG3bWDgaqbamaaBaaaleaacqaIXaqmaeqaaOGaeyO0H4Taem4DaC3aaSbaaSqaaiabikdaYaqabaGccqWF8jcSdaWgaaWcbaGaee4qam0aaSbaaWqaaiabbkdaYaqabaaaleqaaOGafm4DaCNbauaadaWgaaWcbaGaeGOmaidabeaaaaa@4AC8@, *then *(*w*, +) *is in the set*. (*In this case w *= *w*_1 _*is a *threshold, *and *(*w*_1_, *w*_2_) *is a *positive threshold pair *of MLM interaction *(*u*_1_, *u*_2_));

* *if *∃wi∈WCi
 MathType@MTEF@5@5@+=feaafiart1ev1aaatCvAUfKttLearuWrP9MDH5MBPbIqV92AaeXatLxBI9gBaebbnrfifHhDYfgasaacPC6xNi=xH8viVGI8Gi=hEeeu0xXdbba9frFj0xb9qqpG0dXdb9aspeI8k8fiI+fsY=rqGqVepae9pg0db9vqaiVgFr0xfr=xfr=xc9adbaqaaeGacaGaaiaabeqaaeqabiWaaaGcbaGaey4aIqIaem4DaC3aaSbaaSqaaiabdMgaPbqabaGccqGHiiIZcqWGxbWvdaWgaaWcbaGaem4qam0aaSbaaWqaaiabdMgaPbqabaaaleqaaaaa@3539@, *i *= 1, 2, (*w*_1_, *w*_2_) ∈ Ψ_1,2 _*such that: *∃Ψ′1,2⊆Ψ1,2:(w1,w2)∈Ψ′1,2
 MathType@MTEF@5@5@+=feaafiart1ev1aaatCvAUfKttLearuWrP9MDH5MBPbIqV92AaeXatLxBI9gBaebbnrfifHhDYfgasaacPC6xNi=xH8viVGI8Gi=hEeeu0xXdbba9frFj0xb9qqpG0dXdb9aspeI8k8fiI+fsY=rqGqVepae9pg0db9vqaiVgFr0xfr=xfr=xc9adbaqaaeGacaGaaiaabeqaaeqabiWaaaGcbaGaey4aIqIafuiQdKLbauaadaWgaaWcbaGaeGymaeJaeiilaWIaeGOmaidabeaakiabgAOinlabfI6aznaaBaaaleaacqaIXaqmcqGGSaalcqaIYaGmaeqaaOGaeiOoaOJaeiikaGIaem4DaC3aaSbaaSqaaiabigdaXaqabaGccqGGSaalcqWG3bWDdaWgaaWcbaGaeGOmaidabeaakiabcMcaPiabgIGiolqbfI6azzaafaWaaSbaaSqaaiabigdaXiabcYcaSiabikdaYaqabaaaaa@469A@*and *∀(w′1,w′2)∈Ψ′1,2
 MathType@MTEF@5@5@+=feaafiart1ev1aaatCvAUfKttLearuWrP9MDH5MBPbIqV92AaeXatLxBI9gBaebbnrfifHhDYfgasaacPC6xNi=xH8viVGI8Gi=hEeeu0xXdbba9frFj0xb9qqpG0dXdb9aspeI8k8fiI+fsY=rqGqVepae9pg0db9vqaiVgFr0xfr=xfr=xc9adbaqaaeGacaGaaiaabeqaaeqabiWaaaGcbaGaeyiaIiIaeiikaGIafm4DaCNbauaadaWgaaWcbaGaeGymaedabeaakiabcYcaSiqbdEha3zaafaWaaSbaaSqaaiabikdaYaqabaGccqGGPaqkcqGHiiIZcuqHOoqwgaqbamaaBaaaleaacqaIXaqmcqGGSaalcqaIYaGmaeqaaaaa@3A96@, *if *w′1≼C1w1⇒w2≼C2w′2
 MathType@MTEF@5@5@+=feaafiart1ev1aaatCvAUfKttLearuWrP9MDH5MBPbIqV92AaeXatLxBI9gBaebbnrfifHhDYfgasaacPC6xNi=xH8viVGI8Gi=hEeeu0xXdbba9frFj0xb9qqpG0dXdb9aspeI8k8fiI+fsY=rqGqVepae9pg0db9vqaiVgFr0xfr=xfr=xc9adbaqaaeGacaGaaiaabeqaaeqabiWaaaGcbaGafm4DaCNbauaadaWgaaWcbaGaeGymaedabeaatuuDJXwAK1uy0HMmaeHbfv3ySLgzG0uy0HgiuD3BaGabaOGae8hFIa7aaSbaaSqaaiabboeadnaaBaaameaacqqGXaqmaeqaaaWcbeaakiabdEha3naaBaaaleaacqaIXaqmaeqaaOGaeyO0H4Taem4DaC3aaSbaaSqaaiabikdaYaqabaGccqWF8jcSdaWgaaWcbaGaee4qam0aaSbaaWqaaiabbkdaYaqabaaaleqaaOGafm4DaCNbauaadaWgaaWcbaGaeGOmaidabeaaaaa@4AC8@*and if *w1≼C1w′1⇒w′2≼C2w2
 MathType@MTEF@5@5@+=feaafiart1ev1aaatCvAUfKttLearuWrP9MDH5MBPbIqV92AaeXatLxBI9gBaebbnrfifHhDYfgasaacPC6xNi=xH8viVGI8Gi=hEeeu0xXdbba9frFj0xb9qqpG0dXdb9aspeI8k8fiI+fsY=rqGqVepae9pg0db9vqaiVgFr0xfr=xfr=xc9adbaqaaeGacaGaaiaabeqaaeqabiWaaaGcbaGaem4DaC3aaSbaaSqaaiabigdaXaqabaWefv3ySLgznfgDOjdaryqr1ngBPrginfgDObcv39gaiqaakiab=XNiWoaaBaaaleaacqqGdbWqdaWgaaadbaGaeeymaedabeaaaSqabaGccuWG3bWDgaqbamaaBaaaleaacqaIXaqmaeqaaOGaeyO0H4Tafm4DaCNbauaadaWgaaWcbaGaeGOmaidabeaakiab=XNiWoaaBaaaleaacqqGdbWqdaWgaaadbaGaeeOmaidabeaaaSqabaGccqWG3bWDdaWgaaWcbaGaeGOmaidabeaaaaa@4AC8@, *then *(*w*, -) *is in the set*. (*In this case w *= *w*_1 _*is a *threshold, *and *(*w*_1_, *w*_2_) *is a *negative threshold pair *of MLM interaction *(*u*_1_, *u*_2_));

The translated regulatory graph G
 MathType@MTEF@5@5@+=feaafiart1ev1aaatCvAUfKttLearuWrP9MDH5MBPbIqV92AaeXatLxBI9gBaebbnrfifHhDYfgasaacPC6xNi=xH8viVGI8Gi=hEeeu0xXdbba9frFj0xb9qqpG0dXdb9aspeI8k8fiI+fsY=rqGqVepae9pg0db9vqaiVgFr0xfr=xfr=xc9adbaqaaeGacaGaaiaabeqaaeqabiWaaaGcbaWenfgDOvwBHrxAJfwnHbqeg0uy0HwzTfgDPnwy1aaceaGae8NbXFeaaa@3753@ looks very much like a MLM model, but there are still some differences. It may contain several labels by arc, and these labels contains observed values, which are not necessary numerical ones. Thus, the next definition describes how to obtain a family of well formed MLM models from G
 MathType@MTEF@5@5@+=feaafiart1ev1aaatCvAUfKttLearuWrP9MDH5MBPbIqV92AaeXatLxBI9gBaebbnrfifHhDYfgasaacPC6xNi=xH8viVGI8Gi=hEeeu0xXdbba9frFj0xb9qqpG0dXdb9aspeI8k8fiI+fsY=rqGqVepae9pg0db9vqaiVgFr0xfr=xfr=xc9adbaqaaeGacaGaaiaabeqaaeqabiWaaaGcbaWenfgDOvwBHrxAJfwnHbqeg0uy0HwzTfgDPnwy1aaceaGae8NbXFeaaa@3753@.

#### Definition 19 (Labeled directed graphs)

*The family of labeled directed graphs compatible with the translated regulatory graph *G
 MathType@MTEF@5@5@+=feaafiart1ev1aaatCvAUfKttLearuWrP9MDH5MBPbIqV92AaeXatLxBI9gBaebbnrfifHhDYfgasaacPC6xNi=xH8viVGI8Gi=hEeeu0xXdbba9frFj0xb9qqpG0dXdb9aspeI8k8fiI+fsY=rqGqVepae9pg0db9vqaiVgFr0xfr=xfr=xc9adbaqaaeGacaGaaiaabeqaaeqabiWaaaGcbaWenfgDOvwBHrxAJfwnHbqeg0uy0HwzTfgDPnwy1aaceaGae8NbXFeaaa@3753@ = (*U*, ℰ
 MathType@MTEF@5@5@+=feaafiart1ev1aaatCvAUfKttLearuWrP9MDH5MBPbIqV92AaeXatLxBI9gBaebbnrfifHhDYfgasaacPC6xNi=xH8viVGI8Gi=hEeeu0xXdbba9frFj0xb9qqpG0dXdb9aspeI8k8fiI+fsY=rqGqVepae9pg0db9vqaiVgFr0xfr=xfr=xc9adbaqaaeGacaGaaiaabeqaaeqabiWaaaGcbaWenfgDOvwBHrxAJfwnHbqeg0uy0HwzTfgDPnwy1aaceaGae8hmHueaaa@36AB@) *is the set of graphs G *= (*U*, *E*) *constructed in the following way:*

• (*u*, *u'*) ∈ *E iff *(*u*, *u'*) ∈ ℰ
 MathType@MTEF@5@5@+=feaafiart1ev1aaatCvAUfKttLearuWrP9MDH5MBPbIqV92AaeXatLxBI9gBaebbnrfifHhDYfgasaacPC6xNi=xH8viVGI8Gi=hEeeu0xXdbba9frFj0xb9qqpG0dXdb9aspeI8k8fiI+fsY=rqGqVepae9pg0db9vqaiVgFr0xfr=xfr=xc9adbaqaaeGacaGaaiaabeqaaeqabiWaaaGcbaWenfgDOvwBHrxAJfwnHbqeg0uy0HwzTfgDPnwy1aaceaGae8hmHueaaa@36AB@*and it is labeled with at most one of pairs *(*θ*, *ε*) *from the set labelling *(*u*, *u'*) ∈ ℰ
 MathType@MTEF@5@5@+=feaafiart1ev1aaatCvAUfKttLearuWrP9MDH5MBPbIqV92AaeXatLxBI9gBaebbnrfifHhDYfgasaacPC6xNi=xH8viVGI8Gi=hEeeu0xXdbba9frFj0xb9qqpG0dXdb9aspeI8k8fiI+fsY=rqGqVepae9pg0db9vqaiVgFr0xfr=xfr=xc9adbaqaaeGacaGaaiaabeqaaeqabiWaaaGcbaWenfgDOvwBHrxAJfwnHbqeg0uy0HwzTfgDPnwy1aaceaGae8hmHueaaa@36AB@, *if any*.

• *For each node u of the so constructed translated regulatory graph, let us consider the set *Θ_*u *_*of all thresholds occuring on the arcs originating from u. The bound b*_*u *_*associated to u will be the *|Θ_*u*_| + *Nua*, *where Nua is the number of unlabeled arcs originating from u. For each topological sort *(*θ*_1_,...,θbu
 MathType@MTEF@5@5@+=feaafiart1ev1aaatCvAUfKttLearuWrP9MDH5MBPbIqV92AaeXatLxBI9gBaebbnrfifHhDYfgasaacPC6xNi=xH8viVGI8Gi=hEeeu0xXdbba9frFj0xb9qqpG0dXdb9aspeI8k8fiI+fsY=rqGqVepae9pg0db9vqaiVgFr0xfr=xfr=xc9adbaqaaeGacaGaaiaabeqaaeqabiWaaaGcbaacciGae8hUde3aaSbaaSqaaiabdkgaInaaBaaameaacqWG1bqDaeqaaaWcbeaaaaa@30B4@) *of *Θ, *the numerical values *1 ≤ *t *≤ *b*_*u *_*are associated to the corresponding variable values *(*θ*_1_,...,θbu
 MathType@MTEF@5@5@+=feaafiart1ev1aaatCvAUfKttLearuWrP9MDH5MBPbIqV92AaeXatLxBI9gBaebbnrfifHhDYfgasaacPC6xNi=xH8viVGI8Gi=hEeeu0xXdbba9frFj0xb9qqpG0dXdb9aspeI8k8fiI+fsY=rqGqVepae9pg0db9vqaiVgFr0xfr=xfr=xc9adbaqaaeGacaGaaiaabeqaaeqabiWaaaGcbaacciGae8hUde3aaSbaaSqaaiabdkgaInaaBaaameaacqWG1bqDaeqaaaWcbeaaaaa@30B4@), *and each label *(*θ*, *ε*) *is replaced by the corresponding *(*t*, *ε*) *in arc labels*.

• *If *(*u*, *u'*) ∈ ℰ
 MathType@MTEF@5@5@+=feaafiart1ev1aaatCvAUfKttLearuWrP9MDH5MBPbIqV92AaeXatLxBI9gBaebbnrfifHhDYfgasaacPC6xNi=xH8viVGI8Gi=hEeeu0xXdbba9frFj0xb9qqpG0dXdb9aspeI8k8fiI+fsY=rqGqVepae9pg0db9vqaiVgFr0xfr=xfr=xc9adbaqaaeGacaGaaiaabeqaaeqabiWaaaGcbaWenfgDOvwBHrxAJfwnHbqeg0uy0HwzTfgDPnwy1aaceaGae8hmHueaaa@36AB@*has an empty label*, (*u*, *u'*) ∈ *E should be labeled with *(*t*, *ε*) *such that *1 ≤ *t *<*b*_*u *_*and ε *= + *or *-.

A state *μ *of such a graph *G *∈ G
 MathType@MTEF@5@5@+=feaafiart1ev1aaatCvAUfKttLearuWrP9MDH5MBPbIqV92AaeXatLxBI9gBaebbnrfifHhDYfgasaacPC6xNi=xH8viVGI8Gi=hEeeu0xXdbba9frFj0xb9qqpG0dXdb9aspeI8k8fiI+fsY=rqGqVepae9pg0db9vqaiVgFr0xfr=xfr=xc9adbaqaaeGacaGaaiaabeqaaeqabiWaaaGcbaWenfgDOvwBHrxAJfwnHbqeg0uy0HwzTfgDPnwy1aaceaGae8NbXFeaaa@3753@ associates then to the node *u *a numerical value in {0,...,*b*_*u*_} identifying an interval between two successive thresholds.

The MIN representation of biological systems is richer than that of MLM, already because the last does not take into account states of regulatory sites. So, several states of the MIN may be represented by only one state of the MLM. In order to establish the connection between dynamic parameters of both systems, the correspondence between states of them must be introduced: one MLM state corresponds to a domain of states in MIN.

#### Notation 1 (Translation of system states of MIN in MLM)

*If *ℳ
 MathType@MTEF@5@5@+=feaafiart1ev1aaatCvAUfKttLearuWrP9MDH5MBPbIqV92AaeXatLxBI9gBaebbnrfifHhDYfgasaacPC6xNi=xH8viVGI8Gi=hEeeu0xXdbba9frFj0xb9qqpG0dXdb9aspeI8k8fiI+fsY=rqGqVepae9pg0db9vqaiVgFr0xfr=xfr=xc9adbaqaaeGacaGaaiaabeqaaeqabiWaaaGcbaWenfgDOvwBHrxAJfwnHbqeg0uy0HwzTfgDPnwy1aaceaGae83mH0eaaa@36B6@ = (V,ℐCℛ,ℐℛC,ℱ,ℒ
 MathType@MTEF@5@5@+=feaafiart1ev1aaatCvAUfKttLearuWrP9MDH5MBPbIqV92AaeXatLxBI9gBaebbnrfifHhDYfgasaacPC6xNi=xH8viVGI8Gi=hEeeu0xXdbba9frFj0xb9qqpG0dXdb9aspeI8k8fiI+fsY=rqGqVepae9pg0db9vqaiVgFr0xfr=xfr=xc9adbaqaaeGacaGaaiaabeqaaeqabiWaaaGcbaWenfgDOvwBHrxAJfwnHbqeg0uy0HwzTfgDPnwy1aaceaGae8xfXBLaeiilaWIae8heHKKae8NaXpKae83gHiLaeiilaWIae8heHKKae83gHiLae8NaXpKaeiilaWIae8xmHyKaeiilaWIae8NeHWeaaa@44AC@) *is a MIN, and G *= (*U*, *E*) *is one of the family of labeled directed graphs compatible with the translated regulatory graph of *ℳ
 MathType@MTEF@5@5@+=feaafiart1ev1aaatCvAUfKttLearuWrP9MDH5MBPbIqV92AaeXatLxBI9gBaebbnrfifHhDYfgasaacPC6xNi=xH8viVGI8Gi=hEeeu0xXdbba9frFj0xb9qqpG0dXdb9aspeI8k8fiI+fsY=rqGqVepae9pg0db9vqaiVgFr0xfr=xfr=xc9adbaqaaeGacaGaaiaabeqaaeqabiWaaaGcbaWenfgDOvwBHrxAJfwnHbqeg0uy0HwzTfgDPnwy1aaceaGae83mH0eaaa@36B6@, *μ is a state of G*, O
 MathType@MTEF@5@5@+=feaafiart1ev1aaatCvAUfKttLearuWrP9MDH5MBPbIqV92AaeXatLxBI9gBaebbnrfifHhDYfgasaacPC6xNi=xH8viVGI8Gi=hEeeu0xXdbba9frFj0xb9qqpG0dXdb9aspeI8k8fiI+fsY=rqGqVepae9pg0db9vqaiVgFr0xfr=xfr=xc9adbaqaaeGacaGaaiaabeqaaeqabiWaaaGcbaWenfgDOvwBHrxAJfwnHbqeg0uy0HwzTfgDPnwy1aaceaGae8NdX=eaaa@3763@_*μ *_*is the set of states ω *∈ Ω *such that *∀*u *∈ *U if C *∈ C
 MathType@MTEF@5@5@+=feaafiart1ev1aaatCvAUfKttLearuWrP9MDH5MBPbIqV92AaeXatLxBI9gBaebbnrfifHhDYfgasaacPC6xNi=xH8viVGI8Gi=hEeeu0xXdbba9frFj0xb9qqpG0dXdb9aspeI8k8fiI+fsY=rqGqVepae9pg0db9vqaiVgFr0xfr=xfr=xc9adbaqaaeGacaGaaiaabeqaaeqabiWaaaGcbaWenfgDOvwBHrxAJfwnHbqeg0uy0HwzTfgDPnwy1aaceaGae8NaXpeaaa@374B@*is the original species of the variable u then *(*μ*(*u*) = 0 ∧ *ω*(*C*) ≼ *θ*_1_) ∨ (0 <*μ*(*u*) <*b*_*u *_∧ *θ*_*μ*(*u*) _≼ *ω*(*C*) ∧ *ω*(*C*) ≺ *θ*_*μ*(*u*)+1_) ∨ (*μ*(*u*) = *b*_*u *_∧ θbu
 MathType@MTEF@5@5@+=feaafiart1ev1aaatCvAUfKttLearuWrP9MDH5MBPbIqV92AaeXatLxBI9gBaebbnrfifHhDYfgasaacPC6xNi=xH8viVGI8Gi=hEeeu0xXdbba9frFj0xb9qqpG0dXdb9aspeI8k8fiI+fsY=rqGqVepae9pg0db9vqaiVgFr0xfr=xfr=xc9adbaqaaeGacaGaaiaabeqaaeqabiWaaaGcbaacciGae8hUde3aaSbaaSqaaiabdkgaInaaBaaameaacqWG1bqDaeqaaaWcbeaaaaa@30B4@ ≼ *ω*(*C*)). *μ is called the *translated state *of the *domain O
 MathType@MTEF@5@5@+=feaafiart1ev1aaatCvAUfKttLearuWrP9MDH5MBPbIqV92AaeXatLxBI9gBaebbnrfifHhDYfgasaacPC6xNi=xH8viVGI8Gi=hEeeu0xXdbba9frFj0xb9qqpG0dXdb9aspeI8k8fiI+fsY=rqGqVepae9pg0db9vqaiVgFr0xfr=xfr=xc9adbaqaaeGacaGaaiaabeqaaeqabiWaaaGcbaWenfgDOvwBHrxAJfwnHbqeg0uy0HwzTfgDPnwy1aaceaGae8NdX=eaaa@3763@_*μ*_, *and *O
 MathType@MTEF@5@5@+=feaafiart1ev1aaatCvAUfKttLearuWrP9MDH5MBPbIqV92AaeXatLxBI9gBaebbnrfifHhDYfgasaacPC6xNi=xH8viVGI8Gi=hEeeu0xXdbba9frFj0xb9qqpG0dXdb9aspeI8k8fiI+fsY=rqGqVepae9pg0db9vqaiVgFr0xfr=xfr=xc9adbaqaaeGacaGaaiaabeqaaeqabiWaaaGcbaWenfgDOvwBHrxAJfwnHbqeg0uy0HwzTfgDPnwy1aaceaGae8NdX=eaaa@3763@_*μ *_*is the set of *original states *of μ*.

In order to obtain the MLM translation of a MIN, we still need to define the dynamic parameters *K *associated to the possible states of the graphs *G *compatible with G
 MathType@MTEF@5@5@+=feaafiart1ev1aaatCvAUfKttLearuWrP9MDH5MBPbIqV92AaeXatLxBI9gBaebbnrfifHhDYfgasaacPC6xNi=xH8viVGI8Gi=hEeeu0xXdbba9frFj0xb9qqpG0dXdb9aspeI8k8fiI+fsY=rqGqVepae9pg0db9vqaiVgFr0xfr=xfr=xc9adbaqaaeGacaGaaiaabeqaaeqabiWaaaGcbaWenfgDOvwBHrxAJfwnHbqeg0uy0HwzTfgDPnwy1aaceaGae8NbXFeaaa@3753@. The dynamic parameters for a variable are composed of observed states found in ℱ
 MathType@MTEF@5@5@+=feaafiart1ev1aaatCvAUfKttLearuWrP9MDH5MBPbIqV92AaeXatLxBI9gBaebbnrfifHhDYfgasaacPC6xNi=xH8viVGI8Gi=hEeeu0xXdbba9frFj0xb9qqpG0dXdb9aspeI8k8fiI+fsY=rqGqVepae9pg0db9vqaiVgFr0xfr=xfr=xc9adbaqaaeGacaGaaiaabeqaaeqabiWaaaGcbaWenfgDOvwBHrxAJfwnHbqeg0uy0HwzTfgDPnwy1aaceaGae8xmHyeaaa@36AD@ at lines determined by possible values of this variable's resources.

#### Definition 20 (MLM translation)

*If *ℳ
 MathType@MTEF@5@5@+=feaafiart1ev1aaatCvAUfKttLearuWrP9MDH5MBPbIqV92AaeXatLxBI9gBaebbnrfifHhDYfgasaacPC6xNi=xH8viVGI8Gi=hEeeu0xXdbba9frFj0xb9qqpG0dXdb9aspeI8k8fiI+fsY=rqGqVepae9pg0db9vqaiVgFr0xfr=xfr=xc9adbaqaaeGacaGaaiaabeqaaeqabiWaaaGcbaWenfgDOvwBHrxAJfwnHbqeg0uy0HwzTfgDPnwy1aaceaGae83mH0eaaa@36B6@ = (V,ℐCℛ,ℐℛC,ℱ,ℒ
 MathType@MTEF@5@5@+=feaafiart1ev1aaatCvAUfKttLearuWrP9MDH5MBPbIqV92AaeXatLxBI9gBaebbnrfifHhDYfgasaacPC6xNi=xH8viVGI8Gi=hEeeu0xXdbba9frFj0xb9qqpG0dXdb9aspeI8k8fiI+fsY=rqGqVepae9pg0db9vqaiVgFr0xfr=xfr=xc9adbaqaaeGacaGaaiaabeqaaeqabiWaaaGcbaWenfgDOvwBHrxAJfwnHbqeg0uy0HwzTfgDPnwy1aaceaGae8xfXBLaeiilaWIae8heHKKae8NaXpKae83gHiLaeiilaWIae8heHKKae83gHiLae8NaXpKaeiilaWIae8xmHyKaeiilaWIae8NeHWeaaa@44AC@) *is a MIN, its *MLM translation *is a family of instances M *= (*G*, *K*) *such that:*

• *G is one of the family of labeled directed graphs compatible with the translated regulatory graph of *ℳ
 MathType@MTEF@5@5@+=feaafiart1ev1aaatCvAUfKttLearuWrP9MDH5MBPbIqV92AaeXatLxBI9gBaebbnrfifHhDYfgasaacPC6xNi=xH8viVGI8Gi=hEeeu0xXdbba9frFj0xb9qqpG0dXdb9aspeI8k8fiI+fsY=rqGqVepae9pg0db9vqaiVgFr0xfr=xfr=xc9adbaqaaeGacaGaaiaabeqaaeqabiWaaaGcbaWenfgDOvwBHrxAJfwnHbqeg0uy0HwzTfgDPnwy1aaceaGae83mH0eaaa@36B6@;

*K *= {Ku,ωu(μ)
 MathType@MTEF@5@5@+=feaafiart1ev1aaatCvAUfKttLearuWrP9MDH5MBPbIqV92AaeXatLxBI9gBaebbnrfifHhDYfgasaacPC6xNi=xH8viVGI8Gi=hEeeu0xXdbba9frFj0xb9qqpG0dXdb9aspeI8k8fiI+fsY=rqGqVepae9pg0db9vqaiVgFr0xfr=xfr=xc9adbaqaaeGacaGaaiaabeqaaeqabiWaaaGcbaGaem4saS0aaSbaaSqaaiabdwha1jabcYcaSGGaciab=L8a3naaBaaameaacqWG1bqDaeqaaSGaeiikaGIae8hVd0MaeiykaKcabeaaaaa@3653@} *are the dynamic parameters of the MLM instance M where *Ku,ωu(μ)
 MathType@MTEF@5@5@+=feaafiart1ev1aaatCvAUfKttLearuWrP9MDH5MBPbIqV92AaeXatLxBI9gBaebbnrfifHhDYfgasaacPC6xNi=xH8viVGI8Gi=hEeeu0xXdbba9frFj0xb9qqpG0dXdb9aspeI8k8fiI+fsY=rqGqVepae9pg0db9vqaiVgFr0xfr=xfr=xc9adbaqaaeGacaGaaiaabeqaaeqabiWaaaGcbaGaem4saS0aaSbaaSqaaiabdwha1jabcYcaSGGaciab=L8a3naaBaaameaacqWG1bqDaeqaaSGaeiikaGIae8hVd0MaeiykaKcabeaaaaa@3653@*is a set of observable values that the variable u (see Definition 12), the translated variable of C*_*u *_∈ C
 MathType@MTEF@5@5@+=feaafiart1ev1aaatCvAUfKttLearuWrP9MDH5MBPbIqV92AaeXatLxBI9gBaebbnrfifHhDYfgasaacPC6xNi=xH8viVGI8Gi=hEeeu0xXdbba9frFj0xb9qqpG0dXdb9aspeI8k8fiI+fsY=rqGqVepae9pg0db9vqaiVgFr0xfr=xfr=xc9adbaqaaeGacaGaaiaabeqaaeqabiWaaaGcbaWenfgDOvwBHrxAJfwnHbqeg0uy0HwzTfgDPnwy1aaceaGae8NaXpeaaa@374B@, *can have when the MIN state of the system ω is an original state of the state μ of G: if C*_*u' *_∈ C
 MathType@MTEF@5@5@+=feaafiart1ev1aaatCvAUfKttLearuWrP9MDH5MBPbIqV92AaeXatLxBI9gBaebbnrfifHhDYfgasaacPC6xNi=xH8viVGI8Gi=hEeeu0xXdbba9frFj0xb9qqpG0dXdb9aspeI8k8fiI+fsY=rqGqVepae9pg0db9vqaiVgFr0xfr=xfr=xc9adbaqaaeGacaGaaiaabeqaaeqabiWaaaGcbaWenfgDOvwBHrxAJfwnHbqeg0uy0HwzTfgDPnwy1aaceaGae8NaXpeaaa@374B@*is the original variable of u' *∈ *G*^-1^(*u*), Ku,ωu(μ)∈∪u′∈G−1(u)(∪ω∈O(μ)ΨCu′,Cu(ω))
 MathType@MTEF@5@5@+=feaafiart1ev1aaatCvAUfKttLearuWrP9MDH5MBPbIqV92AaeXatLxBI9gBaebbnrfifHhDYfgasaacPC6xNi=xH8viVGI8Gi=hEeeu0xXdbba9frFj0xb9qqpG0dXdb9aspeI8k8fiI+fsY=rqGqVepae9pg0db9vqaiVgFr0xfr=xfr=xc9adbaqaaeGacaGaaiaabeqaaeqabiWaaaGcbaGaem4saS0aaSbaaSqaaiabdwha1jabcYcaSGGaciab=L8a3naaBaaameaacqWG1bqDaeqaaSGaeiikaGIae8hVd0MaeiykaKcabeaakiabgIGiopaatababaWaaeWaaeaadaWeqaqaaiabfI6aznaaBaaaleaacqWGdbWqdaWgaaadbaGafmyDauNbauaaaeqaaSGaeiilaWIaem4qam0aaSbaaWqaaiabdwha1bqabaaaleqaaOWaaeWaaeaacqWFjpWDaiaawIcacaGLPaaaaSqaaiab=L8a3jabgIGioprtHrhAL1wy0L2yHvtyaeHbnfgDOvwBHrxAJfwnaGabaiab+5q8pjabcIcaOiab=X7aTjabcMcaPaqab0GaeSOkIufaaOGaayjkaiaawMcaaaWcbaGafmyDauNbauaacqGHiiIZcqWGhbWrdaahaaadbeqaaiabgkHiTiabigdaXaaaliabcIcaOiabdwha1jabcMcaPaqab0GaeSOkIufaaaa@6365@.

Numerical values are associated to dynamic parameters using the partial order on values of the original species or other information, preserving the order obtained after the threshold ordering.

**Figure 9:**
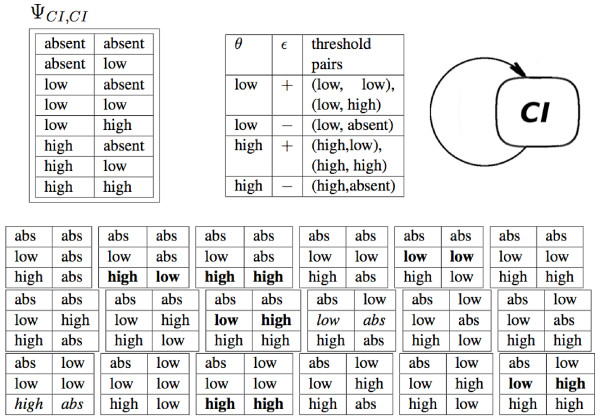
Translation of dynamic parameters from ℱ
 MathType@MTEF@5@5@+=feaafiart1ev1aaatCvAUfKttLearuWrP9MDH5MBPbIqV92AaeXatLxBI9gBaebbnrfifHhDYfgasaacPC6xNi=xH8viVGI8Gi=hEeeu0xXdbba9frFj0xb9qqpG0dXdb9aspeI8k8fiI+fsY=rqGqVepae9pg0db9vqaiVgFr0xfr=xfr=xc9adbaqaaeGacaGaaiaabeqaaeqabiWaaaGcbaWenfgDOvwBHrxAJfwnHbqeg0uy0HwzTfgDPnwy1aaceaGae8xmHyeaaa@36AD@ to MLM. **Left **For the small network, represented on the Figure 3, the interspecies regulation relation Ψ_*CI*,*CI *_is constructed. **Right **The obtained translated regulatory graph and its labels (*θ*, *ε*) with corresponding threshold pairs (shown in **bold **for positive pairs and in *italic *for negative ones in bottom tables). **Bottom **Ordering the *CI *values as *absent *≺_*CI *_*low *≺_*CI *_*high *enables to produce several fully ordered subset of Ψ_*CI*,*CI*_.

The Figure 9 illustrates the dynamic parameters translation from MIN model which is presented in Figure 3.

### Application to the *λ *phage genetic switch

#### Modeling the interacting entities

The chemical species of the model are associated to the chemically active molecules of the system: proteins *CI *and *CRO*, which are able to bind the regulatory sites of the *λ *switch. The regulatory sites named *OR*1, *OR*2 and *OR*3 can be distinguished in the regulatory region of the *λ *switch. Both proteins can bind these regulatory sites. This binding capability will be represented by the affinity labeled *OR*. The regulatory sites will be labeled with the same label *OR*.

The corresponding regulatory DNA regions *OR*1, *OR*2 and *OR*3, controlling the expression of *CI *and *CRO*, are shared by two genes: *cI *and *cro*. It means that the same regulatory site is used to control both genes, and that its state determines the activity level of both proteins simultaneously. So, the influences of *CI *and *CRO *on regulatory sites *OR*1, *OR*2 and *OR*3, and of these sites on the proteins' activity can be added into the model.

The static information about the biological system includes the information about observable values of variables. The observable states of regulatory sites *OR*1, *OR*2 and *OR*3 are "*CI_bound*, *CRO_bound*" or "*free*". Three different observable levels of activity (concentrations) of proteins can be measured: "*absent*", "*low*", "*high*" for *CI *and "*absent*", "*present*", "*high*" for *CRO*.

#### Dynamics of the system

The dynamic description of the biological system in MIN is expressed through the attributes of influences and in relation ℱ
 MathType@MTEF@5@5@+=feaafiart1ev1aaatCvAUfKttLearuWrP9MDH5MBPbIqV92AaeXatLxBI9gBaebbnrfifHhDYfgasaacPC6xNi=xH8viVGI8Gi=hEeeu0xXdbba9frFj0xb9qqpG0dXdb9aspeI8k8fiI+fsY=rqGqVepae9pg0db9vqaiVgFr0xfr=xfr=xc9adbaqaaeGacaGaaiaabeqaaeqabiWaaaGcbaWenfgDOvwBHrxAJfwnHbqeg0uy0HwzTfgDPnwy1aaceaGae8xmHyeaaa@36AD@ (see Figure 8).

The "affinity of *CI *for *OR*1 is tenfold higher than for *OR*2 and *OR*3" [1] can be translated in our formalism by placing the entry (*CI *= *low*; *OR*1 = *CI_bound*, *OR*2 = *free*, *OR*3 = *free*) in ℱ
 MathType@MTEF@5@5@+=feaafiart1ev1aaatCvAUfKttLearuWrP9MDH5MBPbIqV92AaeXatLxBI9gBaebbnrfifHhDYfgasaacPC6xNi=xH8viVGI8Gi=hEeeu0xXdbba9frFj0xb9qqpG0dXdb9aspeI8k8fiI+fsY=rqGqVepae9pg0db9vqaiVgFr0xfr=xfr=xc9adbaqaaeGacaGaaiaabeqaaeqabiWaaaGcbaWenfgDOvwBHrxAJfwnHbqeg0uy0HwzTfgDPnwy1aaceaGae8xmHyeaaa@36AD@.

The property of the *cooperativity *between interacting molecules such as "*CI *bound to *OR*1 increases the affinity of *OR*2 for another tenfold" can be represented in MIN through the refining the information about observabale states by adding the new entries {(*CI *= *low*, *OR*1 = *free*, *OR*2 = *free*) and (*CI *= *low*, *OR*1 = *CI_bound*; *OR*2 = *CI_bound*)} in ℱ
 MathType@MTEF@5@5@+=feaafiart1ev1aaatCvAUfKttLearuWrP9MDH5MBPbIqV92AaeXatLxBI9gBaebbnrfifHhDYfgasaacPC6xNi=xH8viVGI8Gi=hEeeu0xXdbba9frFj0xb9qqpG0dXdb9aspeI8k8fiI+fsY=rqGqVepae9pg0db9vqaiVgFr0xfr=xfr=xc9adbaqaaeGacaGaaiaabeqaaeqabiWaaaGcbaWenfgDOvwBHrxAJfwnHbqeg0uy0HwzTfgDPnwy1aaceaGae8xmHyeaaa@36AD@.

The next type of information concerns the influence of regulatory sites on the protein activity level. The fact that the "Polymerase binding to the *CRO *promoter is disabled if *CI *is bound to *OR*1" can be translated in our formalism by the fact that the protein *CRO *is absent when the *OR*1 site is bound, so we add the entry (*OR*1 = *CI_bound*; *CRO *= *absent*) in ℱ
 MathType@MTEF@5@5@+=feaafiart1ev1aaatCvAUfKttLearuWrP9MDH5MBPbIqV92AaeXatLxBI9gBaebbnrfifHhDYfgasaacPC6xNi=xH8viVGI8Gi=hEeeu0xXdbba9frFj0xb9qqpG0dXdb9aspeI8k8fiI+fsY=rqGqVepae9pg0db9vqaiVgFr0xfr=xfr=xc9adbaqaaeGacaGaaiaabeqaaeqabiWaaaGcbaWenfgDOvwBHrxAJfwnHbqeg0uy0HwzTfgDPnwy1aaceaGae8xmHyeaaa@36AD@.

In the same way the cooperativity could be represented in the expression of *CI*. Its promoter is naturally weak, but it can produce important quantities of *CI *if the site *OR*2 is occupied. This information provides two new entries for the relation ℱ
 MathType@MTEF@5@5@+=feaafiart1ev1aaatCvAUfKttLearuWrP9MDH5MBPbIqV92AaeXatLxBI9gBaebbnrfifHhDYfgasaacPC6xNi=xH8viVGI8Gi=hEeeu0xXdbba9frFj0xb9qqpG0dXdb9aspeI8k8fiI+fsY=rqGqVepae9pg0db9vqaiVgFr0xfr=xfr=xc9adbaqaaeGacaGaaiaabeqaaeqabiWaaaGcbaWenfgDOvwBHrxAJfwnHbqeg0uy0HwzTfgDPnwy1aaceaGae8xmHyeaaa@36AD@ : (*OR*2 = *free*, *CI *= *low*), (*OR*2 = *CI_bound*, *CI *= *high*).

The highest binding affinity of *CRO *is for *OR*3, so that *CRO *rapidly shuts off *CI *production by excluding the RNA polymerase from *CI *promoter, so, another condition for *CI *production is that *OR*3 remains vacant. It can be represented by entries (*OR*3 = *CRO_bound*, *CI *= *absent*) and (*OR*3 = *free*, *CI *= *present*) in ℱ
 MathType@MTEF@5@5@+=feaafiart1ev1aaatCvAUfKttLearuWrP9MDH5MBPbIqV92AaeXatLxBI9gBaebbnrfifHhDYfgasaacPC6xNi=xH8viVGI8Gi=hEeeu0xXdbba9frFj0xb9qqpG0dXdb9aspeI8k8fiI+fsY=rqGqVepae9pg0db9vqaiVgFr0xfr=xfr=xc9adbaqaaeGacaGaaiaabeqaaeqabiWaaaGcbaWenfgDOvwBHrxAJfwnHbqeg0uy0HwzTfgDPnwy1aaceaGae8xmHyeaaa@36AD@.

*Pr*, the *CRO *protein promoter, is inherently a strong one, so as soon as the site *OR*1 is vacant, *CRO *protein is produced, which is represented in MIN by entries (*OR*1 = *CI_bound*, *CRO *= *absent*), (*OR*1 = *CRO_bound*, *CRO *= *absent*) and (*OR*1 = *free*, *CRO *= *high*) in ℱ
 MathType@MTEF@5@5@+=feaafiart1ev1aaatCvAUfKttLearuWrP9MDH5MBPbIqV92AaeXatLxBI9gBaebbnrfifHhDYfgasaacPC6xNi=xH8viVGI8Gi=hEeeu0xXdbba9frFj0xb9qqpG0dXdb9aspeI8k8fiI+fsY=rqGqVepae9pg0db9vqaiVgFr0xfr=xfr=xc9adbaqaaeGacaGaaiaabeqaaeqabiWaaaGcbaWenfgDOvwBHrxAJfwnHbqeg0uy0HwzTfgDPnwy1aaceaGae8xmHyeaaa@36AD@.

**Figure 10:**
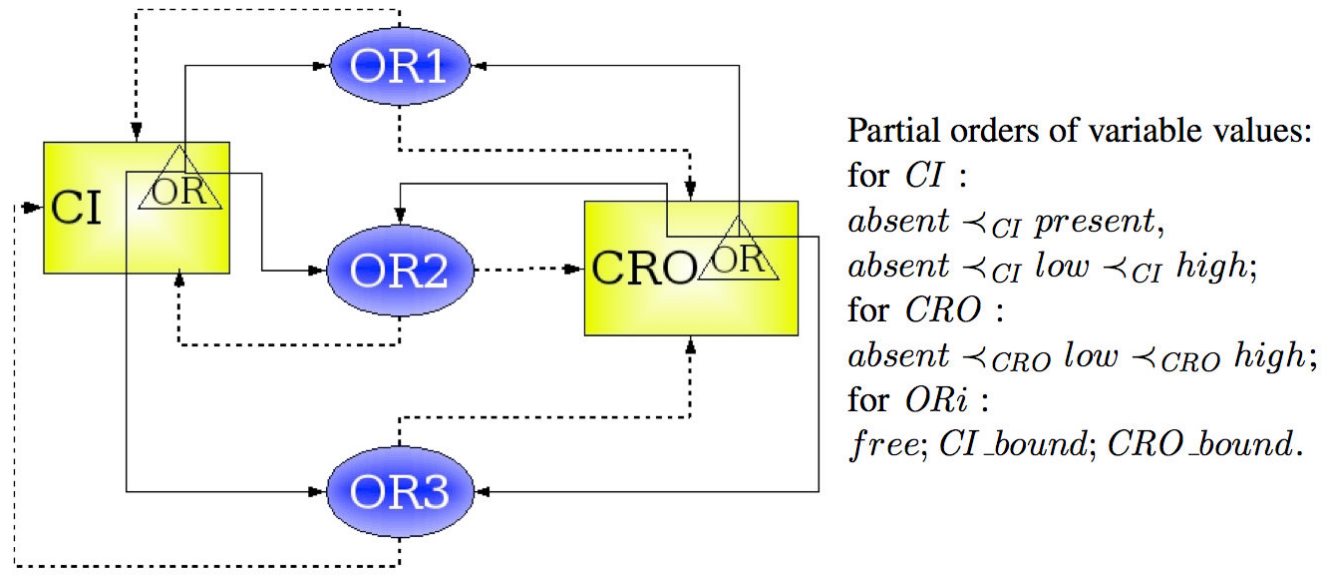
A MIN representing the genetic switch of the *λ *phage. Species *CRO *and *CI *represent proteins which bind with the affinity *OR *to the regulatory sites *OR*1, *OR*2 and *OR*3. These sites are present in the regulatory regions of genes encoding both proteins, so that they influence the corresponding species *CI *and *CRO*. The relation ℱ
 MathType@MTEF@5@5@+=feaafiart1ev1aaatCvAUfKttLearuWrP9MDH5MBPbIqV92AaeXatLxBI9gBaebbnrfifHhDYfgasaacPC6xNi=xH8viVGI8Gi=hEeeu0xXdbba9frFj0xb9qqpG0dXdb9aspeI8k8fiI+fsY=rqGqVepae9pg0db9vqaiVgFr0xfr=xfr=xc9adbaqaaeGacaGaaiaabeqaaeqabiWaaaGcbaWenfgDOvwBHrxAJfwnHbqeg0uy0HwzTfgDPnwy1aaceaGae8xmHyeaaa@36AD@ is the same as in Figure 8.

The resulting MIN is represented in Figure 10.

In order to transform the MIN representation of the *λ *switch in MLM we need to obtain the corresponding interaction graph and the dynamic parameters.

#### Translated interaction graph

The choice of variables of MLM is obvious: variables *CRO *and *CI *will represent the interacting molecular species of the MLM.

We can also follow in the MIN all described interactions between these two variables: *CI *regulates its own expression and the expression of *CRO *through sites *OR*1, *OR*2 and *OR*3. In the following, the *ICR*_*i,a,j *_notation means the *ICR *from the variable *V*_*i *_to the variable *V*_*j *_of MIN through the affinity *a*, and *IRC*_*ij *_means the *IRC *from the variable *V*_*j *_to *V*_*j*_.

(CI,CI)={(ICRCI,OR,OR1+IRCOR1,CI),(ICRCI,OR,OR2+IRCOR2,CI),(ICRCI,OR,OR3+IRCOR3,CI)};(CI,CRO)={(ICRCI,OR,OR1+IRCOR1,CRO),(ICRCI,OR,OR2+IRCOR2,CRO),(ICRCI,OR,OR3+IRCOR3,CRO)}.
 MathType@MTEF@5@5@+=feaafiart1ev1aaatCvAUfKttLearuWrP9MDH5MBPbIqV92AaeXatLxBI9gBaebbnrfifHhDYfgasaacPC6xNi=xI8qiVKYPFjYdHaVhbbf9v8qqaqFr0xc9vqFj0dXdbba91qpepeI8k8fiI+fsY=rqGqVepae9pg0db9vqaiVgFr0xfr=xfr=xc9adbaqaaeGacaGaaiaabeqaaeqabiWaaaGcbaqbaeaabeGaaaqaauaabaqaceaaaeaacqGGOaakcqWGdbWqcqWGjbqscqGGSaalcqWGdbWqcqWGjbqscqGGPaqkcqGH9aqpaeaafaqaaeWacaaabaaabaGaei4EaSNaeiikaGIaemysaKKaem4qamKaemOuai1aaSbaaSqaaiabdoeadjabdMeajjabcYcaSiabd+eapjabdkfasjabcYcaSiabd+eapjabdkfasjabigdaXaqabaGccqGHRaWkcqWGjbqscqWGsbGucqWGdbWqdaWgaaWcbaGaem4ta8KaemOuaiLaeGymaeJaeiilaWIaem4qamKaemysaKeabeaakiabcMcaPiabcYcaSaqaaaqaaiabcIcaOiabdMeajjabdoeadjabdkfasnaaBaaaleaacqWGdbWqcqWGjbqscqGGSaalcqWGpbWtcqWGsbGucqGGSaalcqWGpbWtcqWGsbGucqaIYaGmaeqaaOGaey4kaSIaemysaKKaemOuaiLaem4qam0aaSbaaSqaaiabd+eapjabdkfasjabikdaYiabcYcaSiabdoeadjabdMeajbqabaGccqGGPaqkcqGGSaalaeaaaeaacqGGOaakcqWGjbqscqWGdbWqcqWGsbGudaWgaaWcbaGaem4qamKaemysaKKaeiilaWIaem4ta8KaemOuaiLaeiilaWIaem4ta8KaemOuaiLaeG4mamdabeaakiabgUcaRiabdMeajjabdkfasjabdoeadnaaBaaaleaacqWGpbWtcqWGsbGucqaIZaWmcqGGSaalcqWGdbWqcqWGjbqsaeqaaOGaeiykaKIaeiyFa0Naei4oaSdaaaaaaeaafaqaaeGabaaabaGaeiikaGIaem4qamKaemysaKKaeiilaWIaem4qamKaemOuaiLaem4ta8KaeiykaKIaeyypa0dabaqbaeaabmGaaaqaaaqaaiabcUha7jabcIcaOiabdMeajjabdoeadjabdkfasnaaBaaaleaacqWGdbWqcqWGjbqscqGGSaalcqWGpbWtcqWGsbGucqGGSaalcqWGpbWtcqWGsbGucqaIXaqmaeqaaOGaey4kaSIaemysaKKaemOuaiLaem4qam0aaSbaaSqaaiabd+eapjabdkfasjabigdaXiabcYcaSiabdoeadjabdkfasjabd+eapbqabaGccqGGPaqkcqGGSaalaeaaaeaacqGGOaakcqWGjbqscqWGdbWqcqWGsbGudaWgaaWcbaGaem4qamKaemysaKKaeiilaWIaem4ta8KaemOuaiLaeiilaWIaem4ta8KaemOuaiLaeGOmaidabeaakiabgUcaRiabdMeajjabdkfasjabdoeadnaaBaaaleaacqWGpbWtcqWGsbGucqaIYaGmcqGGSaalcqWGdbWqcqWGsbGucqWGpbWtaeqaaOGaeiykaKIaeiilaWcabaaabaGaeiikaGIaemysaKKaem4qamKaemOuai1aaSbaaSqaaiabdoeadjabdMeajjabcYcaSiabd+eapjabdkfasjabcYcaSiabd+eapjabdkfasjabiodaZaqabaGccqGHRaWkcqWGjbqscqWGsbGucqWGdbWqdaWgaaWcbaGaem4ta8KaemOuaiLaeG4mamJaeiilaWIaem4qamKaemOuaiLaem4ta8eabeaakiabcMcaPiabc2ha9jabc6caUaaaaaaaaaaa@E593@

*CRO *regulates its own expression and the expression of *CI *through the same regulatory sites:

(CRO,CRO)={(ICRCRO,OR,OR1+IRCOR1,CRO),(ICRCRO,OR,OR2+IRCOR2,CRO),(ICRCRO,OR,OR3+IRCOR3,CRO)};(CRO,CI)={(ICRCRO,OR,OR1+IRCOR1,CI),(ICRCRO,OR,OR2+IRCOR2,CI),(ICRCRO,OR,OR3+IRCOR3,CI)}.
 MathType@MTEF@5@5@+=feaafiart1ev1aaatCvAUfKttLearuWrP9MDH5MBPbIqV92AaeXatLxBI9gBaebbnrfifHhDYfgasaacPC6xNi=xI8qiVKYPFjYdHaVhbbf9v8qqaqFr0xc9vqFj0dXdbba91qpepeI8k8fiI+fsY=rqGqVepae9pg0db9vqaiVgFr0xfr=xfr=xc9adbaqaaeGacaGaaiaabeqaaeqabiWaaaGcbaqbaeaabeGaaaqaauaabaqaceaaaeaacqGGOaakcqWGdbWqcqWGsbGucqWGpbWtcqGGSaalcqWGdbWqcqWGsbGucqWGpbWtcqGGPaqkcqGH9aqpaeaafaqaaeWacaaabaaabaGaei4EaSNaeiikaGIaemysaKKaem4qamKaemOuai1aaSbaaSqaaiabdoeadjabdkfasjabd+eapjabcYcaSiabd+eapjabdkfasjabcYcaSiabd+eapjabdkfasjabigdaXaqabaGccqGHRaWkcqWGjbqscqWGsbGucqWGdbWqdaWgaaWcbaGaem4ta8KaemOuaiLaeGymaeJaeiilaWIaem4qamKaemOuaiLaem4ta8eabeaakiabcMcaPiabcYcaSaqaaaqaaiabcIcaOiabdMeajjabdoeadjabdkfasnaaBaaaleaacqWGdbWqcqWGsbGucqWGpbWtcqGGSaalcqWGpbWtcqWGsbGucqGGSaalcqWGpbWtcqWGsbGucqaIYaGmaeqaaOGaey4kaSIaemysaKKaemOuaiLaem4qam0aaSbaaSqaaiabd+eapjabdkfasjabikdaYiabcYcaSiabdoeadjabdkfasjabd+eapbqabaGccqGGPaqkcqGGSaalaeaaaeaacqGGOaakcqWGjbqscqWGdbWqcqWGsbGudaWgaaWcbaGaem4qamKaemOuaiLaem4ta8KaeiilaWIaem4ta8KaemOuaiLaeiilaWIaem4ta8KaemOuaiLaeG4mamdabeaakiabgUcaRiabdMeajjabdkfasjabdoeadnaaBaaaleaacqWGpbWtcqWGsbGucqaIZaWmcqGGSaalcqWGdbWqcqWGsbGucqWGpbWtaeqaaOGaeiykaKIaeiyFa0Naei4oaSdaaaaaaeaafaqaaeGabaaabaGaeiikaGIaem4qamKaemOuaiLaem4ta8KaeiilaWIaem4qamKaemysaKKaeiykaKIaeyypa0dabaqbaeaabmGaaaqaaaqaaiabcUha7jabcIcaOiabdMeajjabdoeadjabdkfasnaaBaaaleaacqWGdbWqcqWGsbGucqWGpbWtcqGGSaalcqWGpbWtcqWGsbGucqGGSaalcqWGpbWtcqWGsbGucqaIXaqmaeqaaOGaey4kaSIaemysaKKaemOuaiLaem4qam0aaSbaaSqaaiabd+eapjabdkfasjabigdaXiabcYcaSiabdoeadjabdMeajbqabaGccqGGPaqkcqGGSaalaeaaaeaacqGGOaakcqWGjbqscqWGdbWqcqWGsbGudaWgaaWcbaGaem4qamKaemOuaiLaem4ta8KaeiilaWIaem4ta8KaemOuaiLaeiilaWIaem4ta8KaemOuaiLaeGOmaidabeaakiabgUcaRiabdMeajjabdkfasjabdoeadnaaBaaaleaacqWGpbWtcqWGsbGucqaIYaGmcqGGSaalcqWGdbWqcqWGjbqsaeqaaOGaeiykaKIaeiilaWcabaaabaGaeiikaGIaemysaKKaem4qamKaemOuai1aaSbaaSqaaiabdoeadjabdkfasjabd+eapjabcYcaSiabd+eapjabdkfasjabcYcaSiabd+eapjabdkfasjabiodaZaqabaGccqGHRaWkcqWGjbqscqWGsbGucqWGdbWqdaWgaaWcbaGaem4ta8KaemOuaiLaeG4mamJaeiilaWIaem4qamKaemysaKeabeaakiabcMcaPiabc2ha9jabc6caUaaaaaaaaaaa@EF5B@

In order to obtain the labels of arcs of the MLM model, the corresponding ΨCi,Ck
 MathType@MTEF@5@5@+=feaafiart1ev1aaatCvAUfKttLearuWrP9MDH5MBPbIqV92AaeXatLxBI9gBaebbnrfifHhDYfgasaacPC6xNi=xH8viVGI8Gi=hEeeu0xXdbba9frFj0xb9qqpG0dXdb9aspeI8k8fiI+fsY=rqGqVepae9pg0db9vqaiVgFr0xfr=xfr=xc9adbaqaaeGacaGaaiaabeqaaeqabiWaaaGcbaGaeuiQdK1aaSbaaSqaaiabdoeadnaaBaaameaacqWGPbqAaeqaaSGaeiilaWIaem4qam0aaSbaaWqaaiabdUgaRbqabaaaleqaaaaa@33B6@ relations are calculated from the relation ℱ
 MathType@MTEF@5@5@+=feaafiart1ev1aaatCvAUfKttLearuWrP9MDH5MBPbIqV92AaeXatLxBI9gBaebbnrfifHhDYfgasaacPC6xNi=xH8viVGI8Gi=hEeeu0xXdbba9frFj0xb9qqpG0dXdb9aspeI8k8fiI+fsY=rqGqVepae9pg0db9vqaiVgFr0xfr=xfr=xc9adbaqaaeGacaGaaiaabeqaaeqabiWaaaGcbaWenfgDOvwBHrxAJfwnHbqeg0uy0HwzTfgDPnwy1aaceaGae8xmHyeaaa@36AD@, as shown in Table 1.

**Table 1:** ΨCi,Ck
 MathType@MTEF@5@5@+=feaafiart1ev1aaatCvAUfKttLearuWrP9MDH5MBPbIqV92AaeXatLxBI9gBaebbnrfifHhDYfgasaacPC6xNi=xH8viVGI8Gi=hEeeu0xXdbba9frFj0xb9qqpG0dXdb9aspeI8k8fiI+fsY=rqGqVepae9pg0db9vqaiVgFr0xfr=xfr=xc9adbaqaaeGacaGaaiaabeqaaeqabiWaaaGcbaGaeuiQdK1aaSbaaSqaaiabdoeadnaaBaaameaacqWGPbqAaeqaaSGaeiilaWIaem4qam0aaSbaaWqaaiabdUgaRbqabaaaleqaaaaa@33B6@ relations calculated from the relation ℱ
 MathType@MTEF@5@5@+=feaafiart1ev1aaatCvAUfKttLearuWrP9MDH5MBPbIqV92AaeXatLxBI9gBaebbnrfifHhDYfgasaacPC6xNi=xH8viVGI8Gi=hEeeu0xXdbba9frFj0xb9qqpG0dXdb9aspeI8k8fiI+fsY=rqGqVepae9pg0db9vqaiVgFr0xfr=xfr=xc9adbaqaaeGacaGaaiaabeqaaeqabiWaaaGcbaWenfgDOvwBHrxAJfwnHbqeg0uy0HwzTfgDPnwy1aaceaGae8xmHyeaaa@36AD@

Ψ_*CI*,*CI*_	Ψ_*CI*,*CRO*_	Ψ_*CRO*,*CI*_	Ψ_*CRO*,*CRO*_
(absent, absent)	(absent, absent)	(absent, absent)	(absent, absent)
(absent, low)	(absent, high)	(absent, low)	(absent, high)
(low, absent)	(low, absent)	(absent, present)	(high, absent)
(low, low)	(high, absent)	(absent, high)	
(low, high)		(low, absent)	
(high, absent)		(high, absent)	
(high, low)			
(high, high)			

Using the Definition 18 of the translated regulatory graph, we can obtain the subsets of ΨCi,Ck
 MathType@MTEF@5@5@+=feaafiart1ev1aaatCvAUfKttLearuWrP9MDH5MBPbIqV92AaeXatLxBI9gBaebbnrfifHhDYfgasaacPC6xNi=xH8viVGI8Gi=hEeeu0xXdbba9frFj0xb9qqpG0dXdb9aspeI8k8fiI+fsY=rqGqVepae9pg0db9vqaiVgFr0xfr=xfr=xc9adbaqaaeGacaGaaiaabeqaaeqabiWaaaGcbaGaeuiQdK1aaSbaaSqaaiabdoeadnaaBaaameaacqWGPbqAaeqaaSGaeiilaWIaem4qam0aaSbaaWqaaiabdUgaRbqabaaaleqaaaaa@33B6@ relations in which the values of *C*_*i *_are fully ordered.

For Ψ_*CI*,*CRO *_and Ψ_*CRO*,*CRO *_two fully ordered subsets can be constructed (see Table 2).

**Table 2:** Fully ordered subsets for Ψ_*CI*,*CRO *_and Ψ_*CRO*,*CRO*_. Here and after, positive threshold pairs are shown in bold, negative threshold pairs are shown in italic

ΨCI,CRO1 MathType@MTEF@5@5@+=feaafiart1ev1aaatCvAUfKttLearuWrP9MDH5MBPbIqV92AaeXatLxBI9gBaebbnrfifHhDYfgasaacPC6xNi=xH8viVGI8Gi=hEeeu0xXdbba9frFj0xb9qqpG0dXdb9aspeI8k8fiI+fsY=rqGqVepae9pg0db9vqaiVgFr0xfr=xfr=xc9adbaqaaeGacaGaaiaabeqaaeqabiWaaaGcbaGaeuiQdK1aa0baaSqaaiabdoeadjabdMeajjabcYcaSiabdoeadjabdkfasjabd+eapbqaaiabigdaXaaaaaa@34EC@	ΨCI,CRO2 MathType@MTEF@5@5@+=feaafiart1ev1aaatCvAUfKttLearuWrP9MDH5MBPbIqV92AaeXatLxBI9gBaebbnrfifHhDYfgasaacPC6xNi=xH8viVGI8Gi=hEeeu0xXdbba9frFj0xb9qqpG0dXdb9aspeI8k8fiI+fsY=rqGqVepae9pg0db9vqaiVgFr0xfr=xfr=xc9adbaqaaeGacaGaaiaabeqaaeqabiWaaaGcbaGaeuiQdK1aa0baaSqaaiabdoeadjabdMeajjabcYcaSiabdoeadjabdkfasjabd+eapbqaaiabikdaYaaaaaa@34EE@	ΨCRO,CRO1 MathType@MTEF@5@5@+=feaafiart1ev1aaatCvAUfKttLearuWrP9MDH5MBPbIqV92AaeXatLxBI9gBaebbnrfifHhDYfgasaacPC6xNi=xH8viVGI8Gi=hEeeu0xXdbba9frFj0xb9qqpG0dXdb9aspeI8k8fiI+fsY=rqGqVepae9pg0db9vqaiVgFr0xfr=xfr=xc9adbaqaaeGacaGaaiaabeqaaeqabiWaaaGcbaGaeuiQdK1aa0baaSqaaiabdoeadjabdkfasjabd+eapjabcYcaSiabdoeadjabdkfasjabd+eapbqaaiabigdaXaaaaaa@3625@	ΨCRO,CRO2 MathType@MTEF@5@5@+=feaafiart1ev1aaatCvAUfKttLearuWrP9MDH5MBPbIqV92AaeXatLxBI9gBaebbnrfifHhDYfgasaacPC6xNi=xH8viVGI8Gi=hEeeu0xXdbba9frFj0xb9qqpG0dXdb9aspeI8k8fiI+fsY=rqGqVepae9pg0db9vqaiVgFr0xfr=xfr=xc9adbaqaaeGacaGaaiaabeqaaeqabiWaaaGcbaGaeuiQdK1aa0baaSqaaiabdoeadjabdkfasjabd+eapjabcYcaSiabdoeadjabdkfasjabd+eapbqaaiabikdaYaaaaaa@3627@
(absent, absent)	(absent, high)	(absent, absent)	(absent, high)
(low, absent)	*(low, absent)*	(high, absent)	*(high, absent)*
(high, absent)	(high, absent)		

Thus, the corresponding arcs of the translated regulatory graph will be labeled with *θ*_*CI*,*CRO *_= *low*, *ε*_*CI*,*CRO *_= "-" and *θ*_*CRO*,*CRO *_= *high*, *ε*_*CRO*,*CRO *_= "-".

For the relation Ψ_*CRO*,*CI*_, four fully ordered subsets can be constructed, as presented in Table 3.

**Table 3:** Four fully ordered subsets for the relation Ψ_*CRO*,*CI*_

ΨCRO,CI1 MathType@MTEF@5@5@+=feaafiart1ev1aaatCvAUfKttLearuWrP9MDH5MBPbIqV92AaeXatLxBI9gBaebbnrfifHhDYfgasaacPC6xNi=xH8viVGI8Gi=hEeeu0xXdbba9frFj0xb9qqpG0dXdb9aspeI8k8fiI+fsY=rqGqVepae9pg0db9vqaiVgFr0xfr=xfr=xc9adbaqaaeGacaGaaiaabeqaaeqabiWaaaGcbaGaeuiQdK1aa0baaSqaaiabdoeadjabdkfasjabd+eapjabcYcaSiabdoeadjabdMeajbqaaiabigdaXaaaaaa@34EC@	ΨCRO,CI2 MathType@MTEF@5@5@+=feaafiart1ev1aaatCvAUfKttLearuWrP9MDH5MBPbIqV92AaeXatLxBI9gBaebbnrfifHhDYfgasaacPC6xNi=xH8viVGI8Gi=hEeeu0xXdbba9frFj0xb9qqpG0dXdb9aspeI8k8fiI+fsY=rqGqVepae9pg0db9vqaiVgFr0xfr=xfr=xc9adbaqaaeGacaGaaiaabeqaaeqabiWaaaGcbaGaeuiQdK1aa0baaSqaaiabdoeadjabdkfasjabd+eapjabcYcaSiabdoeadjabdMeajbqaaiabikdaYaaaaaa@34EE@	ΨCRO,CI3 MathType@MTEF@5@5@+=feaafiart1ev1aaatCvAUfKttLearuWrP9MDH5MBPbIqV92AaeXatLxBI9gBaebbnrfifHhDYfgasaacPC6xNi=xH8viVGI8Gi=hEeeu0xXdbba9frFj0xb9qqpG0dXdb9aspeI8k8fiI+fsY=rqGqVepae9pg0db9vqaiVgFr0xfr=xfr=xc9adbaqaaeGacaGaaiaabeqaaeqabiWaaaGcbaGaeuiQdK1aa0baaSqaaiabdoeadjabdkfasjabd+eapjabcYcaSiabdoeadjabdMeajbqaaiabiodaZaaaaaa@34F0@	ΨCRO,CI4 MathType@MTEF@5@5@+=feaafiart1ev1aaatCvAUfKttLearuWrP9MDH5MBPbIqV92AaeXatLxBI9gBaebbnrfifHhDYfgasaacPC6xNi=xH8viVGI8Gi=hEeeu0xXdbba9frFj0xb9qqpG0dXdb9aspeI8k8fiI+fsY=rqGqVepae9pg0db9vqaiVgFr0xfr=xfr=xc9adbaqaaeGacaGaaiaabeqaaeqabiWaaaGcbaGaeuiQdK1aa0baaSqaaiabdoeadjabdkfasjabd+eapjabcYcaSiabdoeadjabdMeajbqaaiabisda0aaaaaa@34F2@
(absent, absent)	(absent, present)	(absent, low)	(absent, high)
(low, absent)	*(low, absent)*	*(low, absent)*	*(low, absent)*
(high, absent)	(high, absent)	(high, absent)	(high, absent)

Three of four cases lead to the same threshold pair, and the fourth does not have one. So, the arc (*CRO*, *CI*) of the translated regulatory graph should be labeled with *θ*_*CRO*,*CI *_= *low *and *ε*_*CRO*,*CI *_= -.

For the relation Ψ_*CI*,*CI *_18 fully ordered subsets are possible, and they are presented in Figure 9, as well as four labels of the arc (*CI*, *CI*).

**Figure 11:**
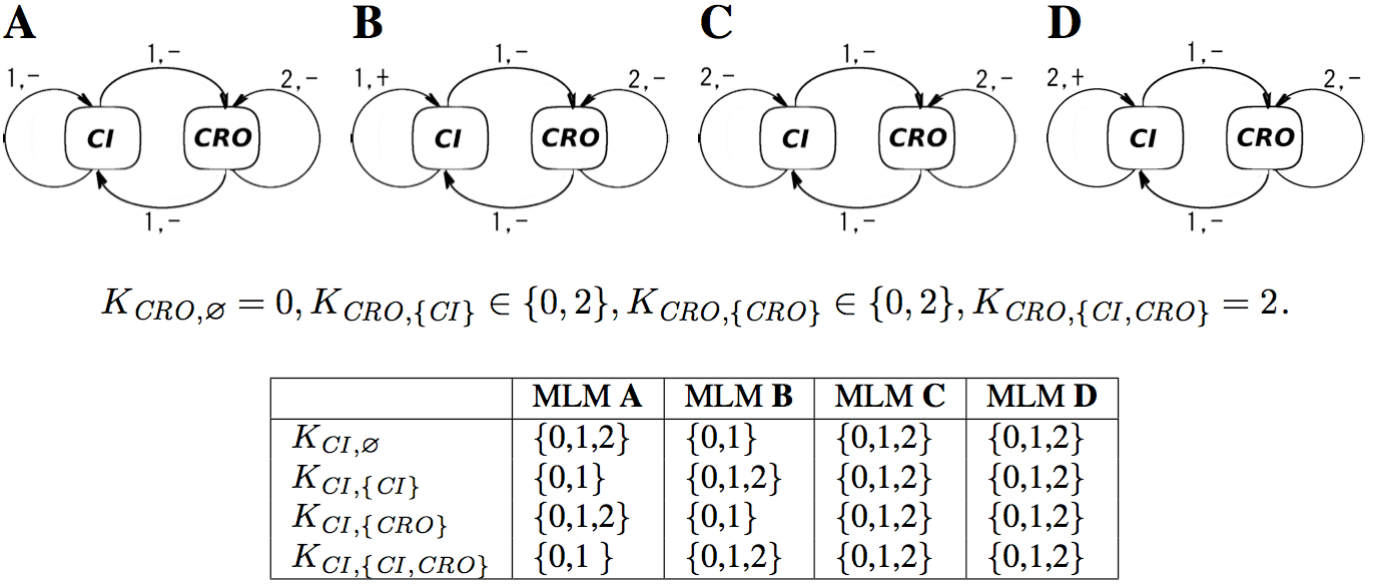
A translation of a MIN from Figure 10 into MLM. The variables *CI *and *CRO *of the MLM are obtained from the species *CI *and *CRO *of the MIN combined with the regulatory sites *OR*1, *OR*2 and *OR*3. The MLM interactions are obtained from pairs (*ICR *+ *IRC*) present in the MIN. For example, there is an arc (*CI*, *CRO*) in the MLM because there is a pair (*ICR *+ *IRC*) = (*CI*, *CRO*) in the MIN presented in Figure 10. The dynamic parameters and arc labels of the MLM are calculated from the relation ℱ
 MathType@MTEF@5@5@+=feaafiart1ev1aaatCvAUfKttLearuWrP9MDH5MBPbIqV92AaeXatLxBI9gBaebbnrfifHhDYfgasaacPC6xNi=xH8viVGI8Gi=hEeeu0xXdbba9frFj0xb9qqpG0dXdb9aspeI8k8fiI+fsY=rqGqVepae9pg0db9vqaiVgFr0xfr=xfr=xc9adbaqaaeGacaGaaiaabeqaaeqabiWaaaGcbaWenfgDOvwBHrxAJfwnHbqeg0uy0HwzTfgDPnwy1aaceaGae8xmHyeaaa@36AD@ of the MIN.

Here we can take an assumption that the MLM can not distinguish between the variable values "present" and "low" and we will attribute the same numerical values to them. Replacing the MIN value "*absent*" by MLM value 0 and thresholds "*low*"/"*present*" *and *"*high*" by numerical values {*1 and 2*}, the family of interaction graphs of the translated MLM of the *λ *switch is obtained (see the Figure 11).

**Dynamic parameters **for every instance of the obtained MLM can be derived from the relations Ψ according to definition of translated parameters.

Dynamic parameters for the variable *CRO *are the same in all three instances and are shown in Table 4.

**Table 4:** Dynamic parameters for the variable *CRO *in the MLM translation

*K*_*CRO*,Ø_	*K*_*CRO*,{*CI*}_	*K*_*CRO*,{*CRO*}_	*K*_*CRO*,{*CI*,*CRO*}_
Ψ_*CI*,*CRO*_(*CI *= high) ∪ Ψ_*CI*,*CRO*_(*CI *= low) ∪ Ψ_*CRO*,*CRO*_(*CRO *= high) = {absent} {0}	Ψ_*CI*,*CRO*_(*CI *= absent) ∪ Ψ_*CRO*,*CRO*_(*CRO *= high) = {high, absent} {0, 2}	Ψ_*CI*,*CRO*_(*CI *= high) ∪ Ψ_*CI*,*CRO*_(*CI *= low) ∪ Ψ_*CRO*,*CRO*_(*CRO *= absent) = {absent, high} {0, 2}	Ψ_*CI*,*CRO*_(*CI *= absent) ∪ Ψ_*CRO*,*CRO*_(*CRO *= absent) = {high} {2}

Dynamic parameters for the variable *CI *can have different values according to the chosen MLM instance. The sets of possible values are shown in Table 5.

**Table 5:** Dynamic parameters for the variable *CI *translated from MIN to MLM. *K*_*CI*,Ø_, *K*_*CI*,{*CI*}_, *K*_*CI*,{*CRO*}_, *K*_*CI*,{*CI*,*CRO*}_

	1, -	1, +	2, -	2, +
*K*_*CI*,Ø_	Ψ_*CI*,*CI*_(*CI *= low) ∪ Ψ_*CI*,*CI*_(*CI *= high) ∪ Ψ_*CRO*,*CI*_(*CRO *= low ) ∪ Ψ_*CRO*,*CI*_(*CRO *= high) = {absent, low, high} {0, 1, 2}	Ψ_*CI*,*CI*_(*CI *= absent) ∪ Ψ_*CRO*,*CI*_(*CRO *= low) ∪ Ψ_*CRO*,*CI*_(*CRO *= high) = {absent, low} {0, 1}	Ψ_*CI*,*CI*_(*CI *= high) ∪ Ψ_*CRO*,*CI*_(*CRO *= low ) ∪ Ψ_*CRO*,*CI*_(*CRO *= high) = {absent, low, high} {0, 1, 2}	Ψ_*CI*,*CI*_(*CI *= absent) ∪ Ψ_*CI*,*CI*_(*CI *= low) ∪ Ψ_*CRO*,*CI*_(*CRO *= low ) ∪ Ψ_*CRO*,*CI*_(*CRO *= high) = {absent, low, high} {0, 1, 2}
*K*_*CI*,{*CI*}_	Ψ_*CI*,*CI*_(*CI *= absent) ∪ Ψ_*CRO*,*CI*_(*CRO *= low ) ∪ Ψ_*CRO*,*CI*_(*CRO *= high) = {absent, low} {0, 1}	Ψ_*CI*,*CI*_(*CI *= low) ∪ Ψ_*CI*,*CI*_(*CI *= high) ∪ Ψ_*CRO*,*CI*_(*CRO *= low ) ∪ Ψ_*CRO*,*CI*_(*CRO *= high) = {absent, low, high} {0, 1, 2}	Ψ_*CI*,*CI*_(*CI *= absent) ∪ Ψ_*CI*,*CI*_(*CI *= low) ∪ Ψ_*CRO*,*CI*_(*CRO *= low ) ∪ Ψ_*CRO*,*CI*_(*CRO *= high) = {absent, low, high} {0, 1, 2}	Ψ_*CI*,*CI*_(*CI *= high) ∪ Ψ_*CRO*,*CI*_(*CRO *= low ) ∪ Ψ_*CRO*,*CI*_(*CRO *= high) = {absent, low, high} {0, 1, 2}
*K*_*CI*,{*CRO*}_	Ψ_*CI*,*CI*_(*CI *= low) ∪ Ψ_*CI*,*CI*_(*CI *= high) ∪ Ψ_*CRO*,*CI*_(*CRO *= absent) = {absent, low, high} {0, 1, 2}	Ψ_*CI*,*CI*_(*CI *= absent) ∪ Ψ_*CRO*,*CI*_(*CRO *= absent) = {absent, low} {0, 1}	Ψ_*CI*,*CI*_(*CI *= high) ∪ Ψ_*CRO*,*CI*_(*CRO *= absent) = {absent, low, high} {0, 1, 2}	Ψ_*CI*,*CI*_(*CI *= absent) ∪ Ψ_*CI*,*CI*_(*CI *= low) ∪ Ψ_*CRO*,*CI*_(*CRO *= absent) = {absent, low, high} {0, 1, 2}
*K*_*CI*,{*CI*,*CRO*}_	Ψ_*CI*,*CI*_(*CI *= low) ∪ Ψ_*CI*,*CI*_(*CI *= high) ∪ Ψ_*CRO*,*CI*_(*CRO *= low) ∪ Ψ_*CRO*,*CI*_(*CRO *= high) = {absent, low} {0, 1}	Ψ_*CI*,*CI*_(*CI *= low) ∪ Ψ_*CI*,*CI*_(*CI *= high) ∪ Ψ_*CRO*,*CI*_(*CRO *= absent) = {absent, low, high} {0, 1, 2}	Ψ_*CI*,*CI*_(*CI *= absent) ∪ Ψ_*CI*,*CI*_(*CI *= low) ∪ Ψ_*CRO*,*CI*_(*CRO *= absent) = {absent, low, high} {0, 1, 2}	Ψ_*CI*,*CI*_(*CI *= high) ∪ Ψ_*CRO*,*CI*_(*CRO *= absent) = {absent, low, high} {0, 1, 2}

This example illustrates the construction of the MIN model from the biological data and shows that this model can be automatically translated in the MLM formalism. In the worst case, the interaction graph of the MLM is constructed from the MIN representation, but no constraint is found on the dynamic parameters (as for parameters KCI,ωμ
 MathType@MTEF@5@5@+=feaafiart1ev1aaatCvAUfKttLearuWrP9MDH5MBPbIqV92AaeXatLxBI9gBaebbnrfifHhDYfgasaacPC6xNi=xH8viVGI8Gi=hEeeu0xXdbba9frFj0xb9qqpG0dXdb9aspeI8k8fiI+fsY=rqGqVepae9pg0db9vqaiVgFr0xfr=xfr=xc9adbaqaaeGacaGaaiaabeqaaeqabiWaaaGcbaGaem4saS0aaSbaaSqaaiabdoeadjabdMeajjabcYcaSGGaciab=L8a3naaBaaameaacqWF8oqBaeqaaaWcbeaaaaa@33E5@ in networks C and D, Figure 11). In the best case, only one value for each dynamic paramter will be produced (as for *K*_*CI*,{*CRO*}_).

### From MIN to ODEs

An important part of the biological knowledge comes from biochemistry. It covers information about the dynamics of *chemical reactions*, which are treated in the *in silico *models through the device of ordinary differential equations (ODEs).

Differential equations aim at expressing the concentration of a chemical species as a function of time, knowing its production and degradation rates:

[P˙]=d[P]dt=∑iki∏j[Sij]αij−∑lkl∏j[Slj]αlj
 MathType@MTEF@5@5@+=feaafiart1ev1aaatCvAUfKttLearuWrP9MDH5MBPbIqV92AaeXatLxBI9gBaebbnrfifHhDYfgasaacPC6xNi=xI8qiVKYPFjYdHaVhbbf9v8qqaqFr0xc9vqFj0dXdbba91qpepeI8k8fiI+fsY=rqGqVepae9pg0db9vqaiVgFr0xfr=xfr=xc9adbaqaaeGacaGaaiaabeqaaeqabiWaaaGcbaGaei4waSLafmiuaaLbaiaacqGGDbqxcqGH9aqpjuaGdaWcaaqaaiabdsgaKjabcUfaBjabdcfaqjabc2faDbqaaiabdsgaKjabdsha0baakiabg2da9maaqafabaGaem4AaS2aaSbaaSqaaiabdMgaPbqabaaabaGaemyAaKgabeqdcqGHris5aOWaaebuaeaacqGGBbWwcqWGtbWudaWgaaWcbaGaemyAaKMaemOAaOgabeaakiabc2faDnaaCaaaleqabaacciGae8xSde2aaSbaaWqaaiabdMgaPjabdQgaQbqabaaaaaWcbaGaemOAaOgabeqdcqGHpis1aOGaeyOeI0YaaabuaeaacqWGRbWAdaWgaaWcbaGaemiBaWgabeaaaeaacqWGSbaBaeqaniabggHiLdGcdaqeqbqaaiabcUfaBjabdofatnaaBaaaleaacqWGSbaBcqWGQbGAaeqaaOGaeiyxa01aaWbaaSqabeaacqWFXoqydaWgaaadbaGaemiBaWMaemOAaOgabeaaaaaaleaacqWGQbGAaeqaniabg+Givdaaaa@6594@

where *k*_*i *_is the reaction rate for the *i*-th *P*-production chemical reaction, *α*_*ij *_is the stoichiometric coefficient of the *j*-th substrate in this reaction, *S*_*ij *_is this substrate, [*S*_*ij*_] is the concentration of the latter, and *k*_*l*_, *α*_*lj*_, [*S*_*lj*_] denote the corresponding elements for the *l*-th *P*-degradation reaction and its co-substrates.

In order to translate the MIN model in ODEs, we need to write the set of chemical reactions in the biological system, and to deduce (if possible) the reaction rates from the parameters of the influences of the MIN model. In a case where the mechanism of the reaction is unknown, it may be written in Michaelis-Menten form: S→EP
 MathType@MTEF@5@5@+=feaafiart1ev1aaatCvAUfKttLearuWrP9MDH5MBPbIqV92AaeXatLxBI9gBaebbnrfifHhDYfgasaacPC6xNi=xH8viVGI8Gi=hEeeu0xXdbba9frFj0xb9qqpG0dXdb9aspeI8k8fiI+fsY=rqGqVepae9pg0db9vqaiVgFr0xfr=xfr=xc9adbaqaaeGacaGaaiaabeqaaeqabiWaaaGcbaGaem4uam1aa4ajaSqaaiabdweafbqabOGaayPKHaGaemiuaafaaa@30D6@, where *E *is an enzyme catalyzing the reaction but not consumed in it. The translation of this reaction into differential equations is a known issue.

**Figure 12:**
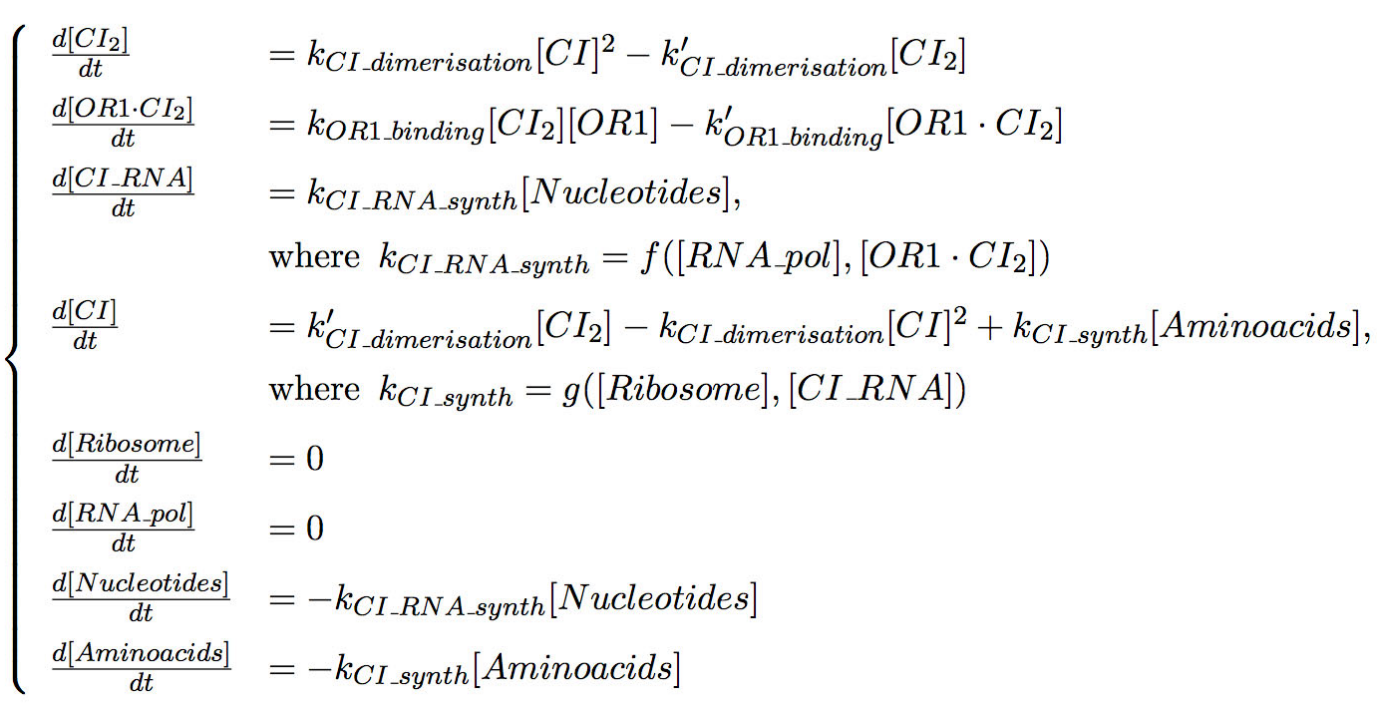
Differential equations obtained by an automatic translation of the MIN model in Figure 4. Functions *f *and *g *come, on one hand, from the MIN topology and the information on the stoichiometry of the reaction, and on the other hand, from the reaction attribute. At this stage, the coherence of both informations should be checked by an expert. In these equations *f *and *g *have a definite signature reflecting the impact of the catalyzers and inhibitors on the reactions.

A MIN model detailed enough to be directly translated to ODEs is presented in Figure 4. For each chemical species in Figure 4 we can write a differential equation summing its consumption and production in chemical reactions the species is participating (see Figure 12). If the additional information is available and encoded in MIN in attributes such as *k*_*i *_and *K*_*aff*_, they will be used in the translation to ODEs procedure. If this information is not available, a free constant denoted in a standard way will be generated. The stoichiometric coefficients give the *α*_*i *_power coefficients in the formula, and the *k*_*j *_reaction rates come form the corresponding reaction attributes.

For example, in the third equation describing the production of the CI RNA from nucleotides, *CI_RNA *corresponds to the quantities of each of the four nucleotides composing the CI RNA: A, U, C and G (the last one, T, being absent from the RNAs). The RNA polymerase (*RNA_pol *in Figure 4) is the enzyme which catalyzes the CI RNA synthesis without being consumed in this reaction, so its concentration influences the reaction rate *k*_*CI_RNA_synth *_and it is taken into account in the function *f*·*OR*1·*CI*_2 _stands for the DNA information source for the CI RNA synthesis, and it acts also as a catalyzer: without this species the CI RNA synthesis is impossible. One molecule of *CI_RNA *species is produced from all the necessary nucleotides on the matrix *OR*1·*CI*_2 _and under the action of the *RNA_pol*. The first equation describes the concentration of the CI protein dimer *CI*_2_. The right part represents the synthesis of one molecule of *CI*_2 _from 2 molecules of *CI *(first term) minus the dissociation of the *CI*_2 _species on 2 *CI *proteins (second term).

More generally, any MIN model can be translated into differential equations with an automated procedure, even if it was not explicitly constructed to represent a set of biochemical reactions. In some cases, it may be necessary to first demulti-ply MIN regulatory sites in order to translate the model directly as for the example in Figure 4.

While the states of a chemical species may characterize the degree of its activity, through a discrete indication like "absent", "low", "high", or through a quantitative information like the concentration, leading quite directly to a representation in ODEs, the states of a regulatory site may potentially be more difficult to interpret. In the simplest case a regulatory site represents a single chemical reaction. The regulatory sites modeling to single chemical reactions, like "CI RNA synthesis", "CI protein synthesis" or "CI dimerisation" in Figure 4, correspond to such a situation, and are easy to translate in ODEs.

However, in a more complex case, a regulatory site may encompass through its different states a family of biochemical reactions, making a direct translation difficult. Actually, the concentrations of participating species for a single chemical reaction are sufficient to find out its activity rate, thus represented by a function. For a family of reactions, the reaction rate is not always a function (but a relation) of the concentrations of each species, and this is precisely the difficulty of the translation to ODEs.

**Figure 13:**
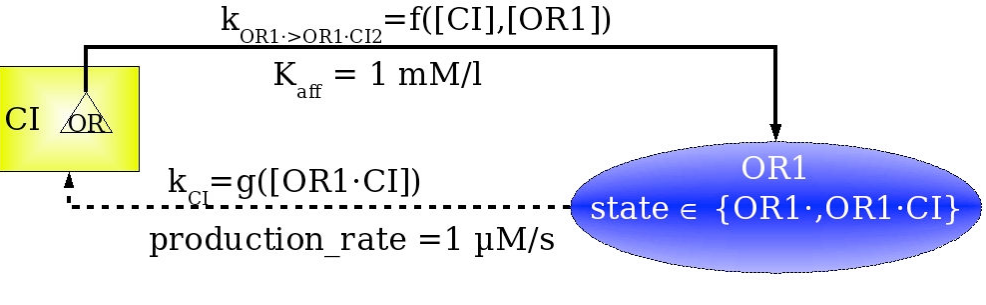
The same MIN model as the one used for genetic regulation modeling, enriched with complementary information allowing the translation into differential equations.

Let us consider the example in Figure 13. The MIN model looks very much like the one in Figure 3, but the IRC and ICR are provided with additional properties such as *k*_*i*_, *K*_*aff *_and *production_rate *which reflect the kinetic properties of the corresponding biochemical reactions. If the regulatory site "OR1" in Figure 13 is in the state *OR*1, it means that neither of the two reactions ("CI RNA synthesis" and "CI protein synthesis") take place in the cell. When the same site is in the state *OR*1·*CI*, it means that both "CI RNA synthesis" and "CI protein synthesis" take place. Thus, it is possible to reduce this complexity by *demultiplicating *the regulatory sites as a first step of the translation of a MIN model in ODEs. The *demultiplication *of a regulatory site *R *replaces it by a set of (new) species associated to the states of *R *and a set of (new) regulatory sites associated to the chemical reactions. In other words, every regulatory state of *R *will now give a chemical species participating in a defined set of chemical reactions, represented by newly generated regulatory sites. After the demultiplication, each regulatory site represents a single chemical reaction, which means that the species connected to it may potentially be produced or consumed, and may be automatically translated to ODEs. Some optimizations may be performed at this stage, for instance, if one knows if the species are consumed or produced, which may be indicated in the attributes (such as "stoichiometry", "production rate", "degradation rate" or "kinetic rate") of the corresponding influences ICRs and IRCs.

## Discussion

The MIN representation proposes a rich formal description of biological interaction networks. The methodology of modelling biological systems in an incremental MIN representation is illustrated by a case study on the *λ *switch system. The formalisation of biological data is independent of any given modeling or simulation approach. The main goal of MIN is to contain as many different data about interacting entities as possible in order to make them accessible to any particular modeling approach. A translation into R. Thomas' formalism allows the modeler to obtain an MLM model from the available data, and the MLM is consistent with other models of the same system [11]. While the translation from MIN into MLM is rather complicated, it can be easily automated using the algorithm presented in this paper. However, without the expert intervention, the number of MLM models can be high. The modeler can act on the data put into the MIN model, changing and refining it, and this change will have an impact on the produced MLM translated models. However, there is no need for an expert to deeply understand the algorithm itself. The translation of MLM instances can be further continued into Petri nets as studied in [2] and, thus, provides an access to the available Petri net tools for analysis. Each formalism has its advantages and fits the description of a certain data type, the complete and efficient description of biological systems is possible only by combining these tools. A formalism forces an interpretation of available data in order to fit them in its framework. Some data which are incompatible with the chosen framework will inevitably be lost. Sometimes the same model represented in different formalisms can hardly be recognized [13,1,5,4].

The situation where a MIN variable have a high number of observed (quantitative or qualitative) values may occur. However, this is not necessarily a problem, as the fact of having a lot of observations for the same variable means that the corresponding biological object plays an important role in the biological process being studied. In this case, every species regulated by this object through a regulatory site is supposed to generate a logical threshold of action. In addition, the fact that several quantitative values are not significantly different is the additional information, which, if available, may be encoded in the partial order for the variable values as a class of equivalence for several variable values.

The representation of regulatory sites and affinities separately from chemical species helps to represent in a "formal" way large proteins with many functional domains, or a complex set of regulatory sites in a protein or in a gene. The specificity of the *λ *phage genetic switch is that the promoter region of two different genes is represented by the same biological object (DNA region). This fact is represented in our formalism by having only one set of regulatory sites of the *λ *switch which influence two different species: *CI *and *CRO*.

MIN enables an incremental model construction through the composition of MINs and the storage (in the species affinities and regulatory site labels) of the information about possible interaction capabilities of biological entities. Thus, MIN can help in the model construction by a rational choice of new variables to be added to the model: with compatible regulatory sites or affinities.

Experimental techniques in biology collect massive amounts of information on the behavior and interaction of thousands of genes and proteins across diverse conditions. These techniques are used to question complex biological systems that use highly intricate regulatory mechanisms and control schemes. One cannot fully characterize such complex cellular systems by focusing on a single control mechanism, as measured by a single experimental technique. In MIN, the data coming from different experimental techniques are all stored in ℱ
 MathType@MTEF@5@5@+=feaafiart1ev1aaatCvAUfKttLearuWrP9MDH5MBPbIqV92AaeXatLxBI9gBaebbnrfifHhDYfgasaacPC6xNi=xH8viVGI8Gi=hEeeu0xXdbba9frFj0xb9qqpG0dXdb9aspeI8k8fiI+fsY=rqGqVepae9pg0db9vqaiVgFr0xfr=xfr=xc9adbaqaaeGacaGaaiaabeqaaeqabiWaaaGcbaWenfgDOvwBHrxAJfwnHbqeg0uy0HwzTfgDPnwy1aaceaGae8xmHyeaaa@36AD@. To gain a deeper understanding of the system, it is pertinent to analyze heterogeneous data sources in a truly integrated fashion and to shape the analysis results into one body of knowledge [14,15].

We proposed a new paradigm for the modeling of biological systems, in which all available experimental data are considered as a set of snapshots of the real system and stored in ℱ
 MathType@MTEF@5@5@+=feaafiart1ev1aaatCvAUfKttLearuWrP9MDH5MBPbIqV92AaeXatLxBI9gBaebbnrfifHhDYfgasaacPC6xNi=xH8viVGI8Gi=hEeeu0xXdbba9frFj0xb9qqpG0dXdb9aspeI8k8fiI+fsY=rqGqVepae9pg0db9vqaiVgFr0xfr=xfr=xc9adbaqaaeGacaGaaiaabeqaaeqabiWaaaGcbaWenfgDOvwBHrxAJfwnHbqeg0uy0HwzTfgDPnwy1aaceaGae8xmHyeaaa@36AD@ without any interpretation. The information about the system is added and refined incrementally. The current state of knowledge in MIN can be automatically translated into a given formalism framework for the analysis of the dynamics of the system; it could also be used in the future by an inference system applying artificial intelligence techniques [16] to solve complex biological problems.

**Figure 14:**
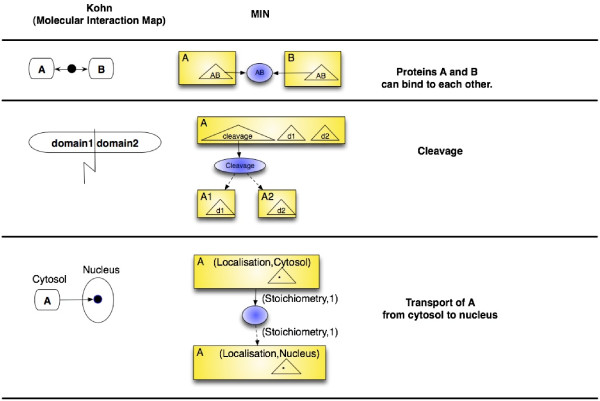
Examples of Kohn Maps building blocks and their MIN representations.

Over the last few years, some work has been carried out in the field of integration of biological and, in particular, biochemical data which includes rich but informal visualisation conventions [17,18]. Even if MIN is not designed as a graphical model, it provides a quite simple visualisation convention with two types of nodes and two types of links. However, combined with textual information encoded in the attributes of links and nodes, it can represent biological features encoded as Kohn Maps [17], as it is illustrated for three examples of Kohn Maps building blocks in Figure 14.

Recently, a method for representing and communicating biological networks in both human and machine readable form has been presented in [19]. The ambition of this work is obtaining a semantically and visually unambiguous diagram scheme, but this leads to a very low level representation of processes and the use of many kinds of nodes and links. Compared to this, MIN does not require an equivalent degree of details and enables to adjust the abstraction level of the model. Another approach, based on formal but not very expressive exchange formalisms, like SBML [20], attempts to standardize the expression of ODE based models of cellular systems, concentrating on chemical reactions. Obviously, existing SBML models can be wrapped in a MIN description. In the same standardisation effort more abstract and universal meta-modelling approaches [21-24] tend to create a general visual language for systems biology, similar to UML. For instance, BioUML [24] provides an abstract layer to present structure of any biological system as a clustered graph. MIN should be expressed in this language to use the infrastructure based on BioUML, to access to the biological databases and to automatically generate the executable models.

Thus, the proposed new formalism, MIN, can play the role of an intermediate level between insufficiently formalized "natural language" and too specialized "mathematical descriptions" of biological systems. The MIN construction is a process of inference of the biological interaction networks from the biological observations of microscopic and macroscopic levels. Its underlying structure provides a skeleton for the understanding of "first principles" of the organisation of biological systems. A computer analysis tool to study the properties of MIN models, to perform automatically their composition and translation into different formalisms, is currently under developed and should soon become available for download. The study of the relation between the information available in MIN and the best suited model is on of the perspectives of this project.

## Conclusion

The description of a biological system is often obtained by constructing an *interaction network*. Intuitively, as biological interactions are considered to always rely on so called *regulatory sites*, the network construction starts by their identification. Every regulatory site has a set of regulating and regulated *chemical species *and their role is expressed by *influences*. Sometimes, and in particular when the abstraction level is high, the choice of representing a set of biochemical reactions by a species or by a regulatory site is rather arbitrary. However, at the base level the chemical reactions are represented by regulatory sites and chemical species by species of MIN. Furthermore, both species and regulatory sites are fully characterized by their levels of activity indicated (as string value) in the modeler's description of the states of a biological system. For the translation into other formalisms the values of the level of activity may be interpreted, if allowed by the target formalism, or ignored. As a consequence, regulatory sites and chemical species form the set of *variables *of the interaction network (see Table 6 for some examples of variables). Thus, two main classes of abstract entities are chosen to be components of interaction networks: *variables *and *influences *between them. We consider two kinds of *influences *between the variables of the model: *Influences of Chemical species on Regulatory sites (ICR) *and *Influences of Regulatory sites on Chemical species (IRC)*. We also assume that there is no influence between variables of the same kind. The whole representation is called Modular Interaction Network (MIN).

**Table 6:** Examples of representations of biological objects in MIN according to their biological function, either of a catalytic or regulatory nature

**biological object**	**Role**	**Model entity**
Gene	information storage and propagation	species
regulatory sequence of DNA	regulation of gene activity	regulatory site
Protein	catalysis	species
phosphorylation or cleavage site	regulation of protein activity	regulatory site
metabolic pathway	transformation of molecules	regulatory site
receptor on a cell surface ...	detecting environmental state ...	regulatory site ...

Such models may be composed. The trivial case of a composition is the union of models having no common species or sites. The union of data contained in these models is the new, composed, model. In the case of models sharing common entities, the repeated nodes of the resulting network are collapsed.

MIN being an abstract formalism, its semantics is not intended to be defined directly, but rather as a translation into a target model. In this paper, we first define a translation of MIN into the Multivalued Logical modeling formalism (MLM) [7].

The multivalued logical representation of genetic regulatory networks [7] is one of the closest to the biological intuition. The major problem of this formalism is that it is not incremental, which means that updating an existing model (by adding or removing nodes or edges in the regulatory graph, for instance) leads to the situation where the set of dynamic parameters changes in an unpredictable way, as well as the dynamics of the system. In order to cope with this problem, the idea is to describe the biological system in MIN and translate it automatically, when needed, at any modeling step, into the multivalued logical formalism. This translation should preserve as many as possible of the biological properties already expressed in MIN. The dynamics of the translated MIN is then based on the information available in the attributes of its influences. The interaction graph can be obtained more or less directly from the MIN presentation of a biological regulatory network. The variables of the MLM (nodes of the graph) are obtained from the species of the MIN. The influences of MLM (edges of the graph) are obtained from pairs of (*ICR*, *IRC*) present in the MIN and having a common regulatory site. The dynamic parameters of MIN indicated as attributes of its influences will serve to constrain possible dynamic parameters in the obtained multivalued logical model.

In order to further illustrate the flexibility of the MIN approach, we have also shown how to extract the dynamics of the associated chemical reactions in terms of ordinary differential equations, either directly or through a demultiplication of the regulatory sites which may represent various different reactions.

## Competing interests

The author(s) declare that they have no competing interests.

## Authors' contributions

AY carried out the general idea of the formalism and the translations to MLM and ODE, and drafted the manuscript. HK and RD worked on the mathematical and logical aspects of definitions and their coherence. All authors participated in the design and scientific positioning. All authors read and approved the final manuscript.
